# Pyrazole Incorporated New Thiosemicarbazones: Design, Synthesis and Investigation of DPP-4 Inhibitory Effects

**DOI:** 10.3390/molecules25215003

**Published:** 2020-10-28

**Authors:** Belgin Sever, Hasan Soybir, Şennur Görgülü, Zerrin Cantürk, Mehlika Dilek Altıntop

**Affiliations:** 1 Department of Pharmaceutical Chemistry, Faculty of Pharmacy, Anadolu University, 26470 Eskişehir, Turkey; belginsever@anadolu.edu.tr (B.S.); hasan_soybir@hotmail.com (H.S.); 2 Medicinal Plant, Drug and Scientific Research and Application Center, Anadolu University, 26470 Eskişehir, Turkey; sennur@gmail.com; 3 Department of Pharmaceutical Microbiology, Faculty of Pharmacy, Anadolu University, 26470 Eskişehir, Turkey; zkcanturk@anadolu.edu.tr

**Keywords:** dipeptidyl peptidase-4, thiosemicarbazone, pyrazole, molecular docking

## Abstract

Dipeptidyl peptidase-4 (DPP-4) inhibition has been recognized as a promising approach to develop safe and potent antidiabetic agents for the management of type 2 diabetes. In this context, new thiosemicarbazones (**2a**–**o**) were prepared efficiently by the reaction of aromatic aldehydes with 4-[4-(1*H*-pyrazol-1-yl)phenyl]thiosemicarbazide (**1**), which was obtained via the reaction of 4-(1*H*-pyrazol-1-yl)phenyl isothiocyanate with hydrazine hydrate. Compounds **2a**–**o** were evaluated for their DPP-4 inhibitory effects based on a convenient fluorescence-based assay. 4-[4-(1*H*-pyrazol-1-yl)phenyl]-1-(4-bromobenzylidene)thiosemicarbazide (**2f**) was identified as the most effective DPP-4 inhibitor in this series with an IC_50_ value of 1.266 ± 0.264 nM when compared with sitagliptin (IC_50_ = 4.380 ± 0.319 nM). MTT test was carried out to assess the cytotoxic effects of compounds **2a**–**o** on NIH/3T3 mouse embryonic fibroblast (normal) cell line. According to cytotoxicity assay, compound **2f** showed cytotoxicity towards NIH/3T3 cell line with an IC_50_ value higher than 500 µM pointing out its favourable safety profile. Molecular docking studies indicated that compound **2f** presented π-π interactions with Arg358 and Tyr666 via pyrazole scaffold and 4-bromophenyl substituent, respectively. Overall, in vitro and in silico studies put emphasis on that compound **2f** attracts a great notice as a drug-like DPP-4 inhibitor for further antidiabetic research.

## 1. Introduction

Diabetes Mellitus (DM) is a complex, metabolic and chronic disorder and a serious threat to human health. DM is mainly divided into two types including type 1 or insulin-dependent DM (T1DM) and type 2 or non-insulin-dependent DM (T2DM). T2DM, which is generally attributed to insulin resistance in target cells and insufficient production of insulin from the β-cells, affects the majority of diabetic patients (around 90–95%) worldwide [1,2,3]. There are current therapy options for T2DM management such as sulphonylureas, glinides and insulin therapy, whereas these antidiabetic medications have an increased risk of hypoglycaemia, obesity and oedema. Therefore, there is an urgent need to discover new weight neutral and well-tolerated antidiabetic agents with a lower risk of hypoglycemia [4,5].

Incretin-based activity plays a pivotal role in the metabolic modulation of T2DM [6]. Glucose-dependent insulinotropic peptide (GIP) and glucagon-like peptide-1 (GLP-1) are incretin hormones secreted by intestinal endocrine cells in response to incoming nutrients and they are responsible for nearly 60% of the postprandial insulin release [7,8]. Based on the potent glucose-lowering actions of these hormones, GLP-1 receptor agonists and dipeptidyl peptidase-4 (DPP-4) inhibitors have been developed as incretin mimetics and incretin enhancers, respectively [9,10].

**Figure 1:**
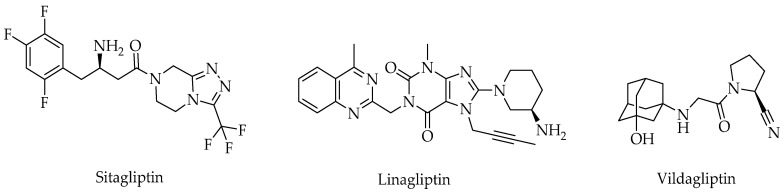
Some DPP-4 inhibitors.

DPP-4 is a serine exopeptidase, which is able to cleave dipeptides from polypeptides such as incretin hormones, some chemokines and cytokines with a proline or alanine amino acid in the penultimate position at the *N*-terminus of the peptide chain. This outcome indicates that DPP-4 is not only implicated in glucose homeostasis but also in neurogenic inflammation, blood pressure and the immune system [8,11,12,13,14]. DPP-4 inhibitors have been reported to stimulate insulin secretion and suppress glucagon secretion in a glucose-dependent way causing a low risk of hypoglycaemia and weight gain compared to other antidiabetic agents. “Gliptins” such as sitagliptin, linagliptin and vildagliptin are well-known DPP-4 inhibitors (Figure 1). Among them, sitagliptin and vildagliptin improve glycaemic control and reduce glycosylated haemoglobin (HbA1c) level in monotherapy or combined therapy with metformin and thiazolidinediones. Some gliptins also possess protective properties in kidney functions and cardiovascular system [15,16,17,18].

**Figure 2:**
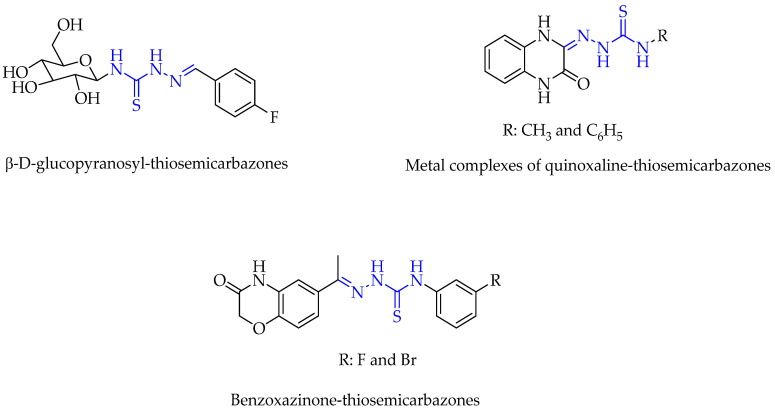
Some thiosemicarbazones endowed with antidiabetic activity.

Thiosemicarbazones have gained extensive interest, in particular with their capacity of forming metal ion coordination modes and metal complexes. They are one of the most important members of N,S donor ligands and have been reported to show a wide pharmacological utility including antidiabetic activity. There are also studies suggesting thiosemicarbazones as aldose reductase and glycogen phosphorylase inhibitors to be effective in T2DM treatment [19,20,21,22,23,24]. Among these studies, Alexacou et al. synthesized some new β-d-glucopyranosyl-thiosemicarbazone derivatives and investigated their glycogen phosphorylase inhibitory effects by kinetic experiments and crystallographic binding studies. *Para* fluorophenyl substituted compound was identified as the most potent glycogen phosphorylase inhibitor (IC_50_ = 5.7 µM) (Figure 2). In another study, Kulkarni et al. prepared new transition metal complexes of quinoxaline–thiosemicarbazone ligands and investigated their antidiabetic activity using blood-glucose and oral glucose tolerance tests. They found that some of them showed significant antidiabetic effects with low toxicity and a high safety profile (Figure 2). On the other hand, Shehzad et al. reported a new series of benzoxazinone-thiosemicarbazones as aldose reductase inhibitors. *Meta* floro and bromophenyl substituted compounds revealed the most significant aldose reductase inhibition (Figure 2) [20,21,24].

**Figure 3:**
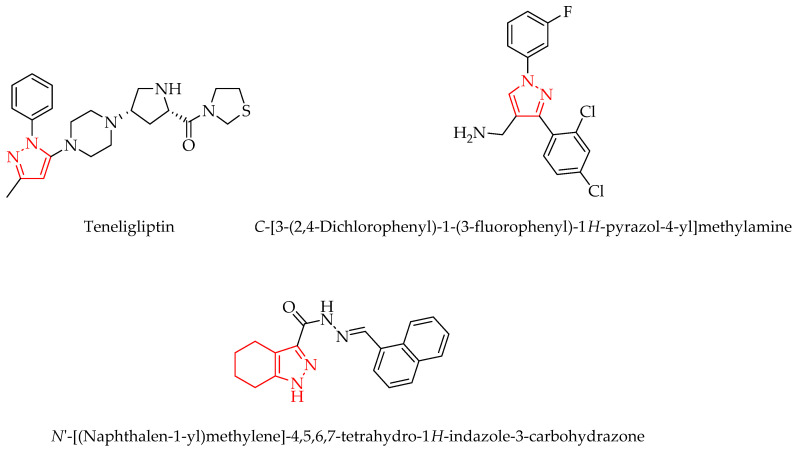
Some pyrazole derivatives as DPP-4 inhibitors.

Pyrazole is a five-membered, heterocyclic compound with two adjacent nitrogen atoms. Pyrazole nucleus can react with both acids and bases due to its distinct nitrogen atoms exhibiting “pyrrole-like” (N1) and “pyridine-like” (N2) behaviours. Pyrazole derivatives have been reported to exhibit a broad spectrum of pharmacological properties including antidiabetic activity and it is also found in a large variety of biologically active molecules. They are prominent competitive inhibitors or activators for antidiabetic drug design such as sodium-glucose co-transporter-1, sodium-glucose cotransporter-2, α-glucosidase, α-amylase and DPP-4 inhibitors, glucagon receptor antagonists and peroxisome proliferator-activated agonists [25,26,27,28,29,30,31,32]. Among these distinct types of antidiabetic activity, there are studies reporting pyrazole-based compounds as DPP-4 inhibitors. The extensive efforts of Yoshida et al. led to the discovery of tenegliptin, which has been approved for the treatment of type 2 diabetes in Japan (Figure 3). Besides, Hsu et al. also designed (1,3-diphenyl-1*H*-pyrazol-4-yl)methylamine analogues as DPP-4 inhibitors, and *meta* fluoro substituted compound was found to slightly increase DPP-4 inhibition according to in vitro studies (Figure 3). In another study, Wu et al. determined a lead compound after bioassay verification and they synthesized analogues of this lead compound. They showed that nine compounds exhibited DPP-4 inhibition. Naphthalen-1-yl bearing compound displayed the most potent inhibitory effect (Figure 3) [33,34,35].

Prompted by the aforementioned findings, pyrazole-based new thiosemicarbazones (**2a**–**o**) were designed and synthesized. The DPP-4 inhibitory effects of all these compounds were determined and they were further evaluated for their cytotoxicities. Moreover, in silico molecular docking and pharmacokinetic studies were carried out to shed light on their mechanistic effects and pharmacokinetic profiles, respectively.

## 2. Results and Discussion

### 2.1. Chemistry

**Scheme 1:**
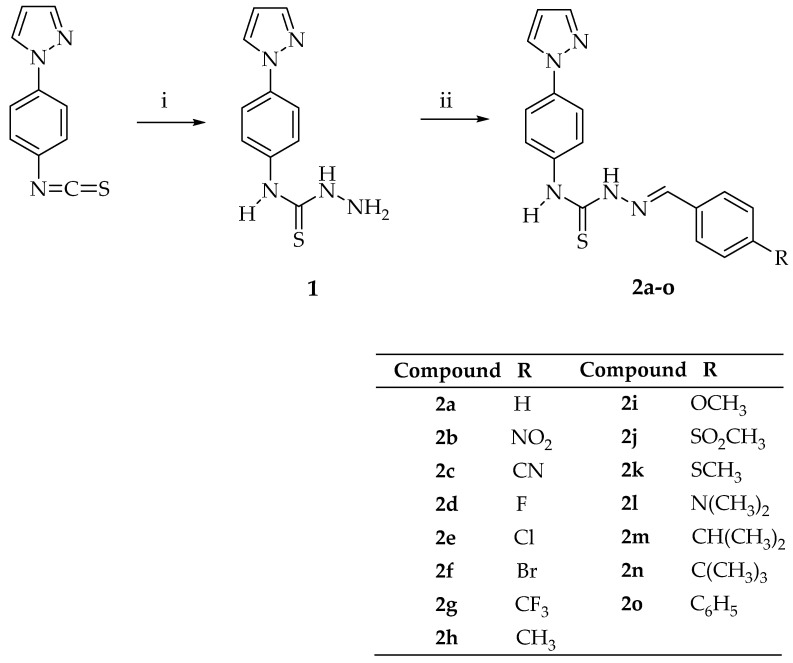
The synthetic route for the preparation of compounds **2a**–**o**. Reagents and conditions: (i) NH_2_NH_2_·H_2_O, ethanol, rt, 4 h; (ii) ArCHO, ethanol, reflux, 8 h.

The synthesis of a new series of thiosemicarbazones (**2a**–**o**) was performed as depicted in Scheme 1. The final compounds were obtained by the treatment of aromatic aldehydes with 4-[4-(1*H*-pyrazol-1-yl)phenyl]thiosemicarbazide (**1**), which was synthesized via the reaction of 4-(1*H*-pyrazol-1-yl)phenyl isothiocyanate with hydrazine hydrate.

The structures of compounds **2a**–**o** were confirmed by different spectroscopic techniques, namely Infrared (IR), ^1^H Nuclear Magnetic Resonance (NMR), ^13^C NMR, and High-Resolution Mass Spectrometry (HRMS).

In the IR spectra of compounds **2a**–**o**, the characteristic N-H stretching vibrations belonging to the thiosemicarbazone moiety were observed in the region 3346.50–3282.84 cm^−1^.

**Figure 4:**
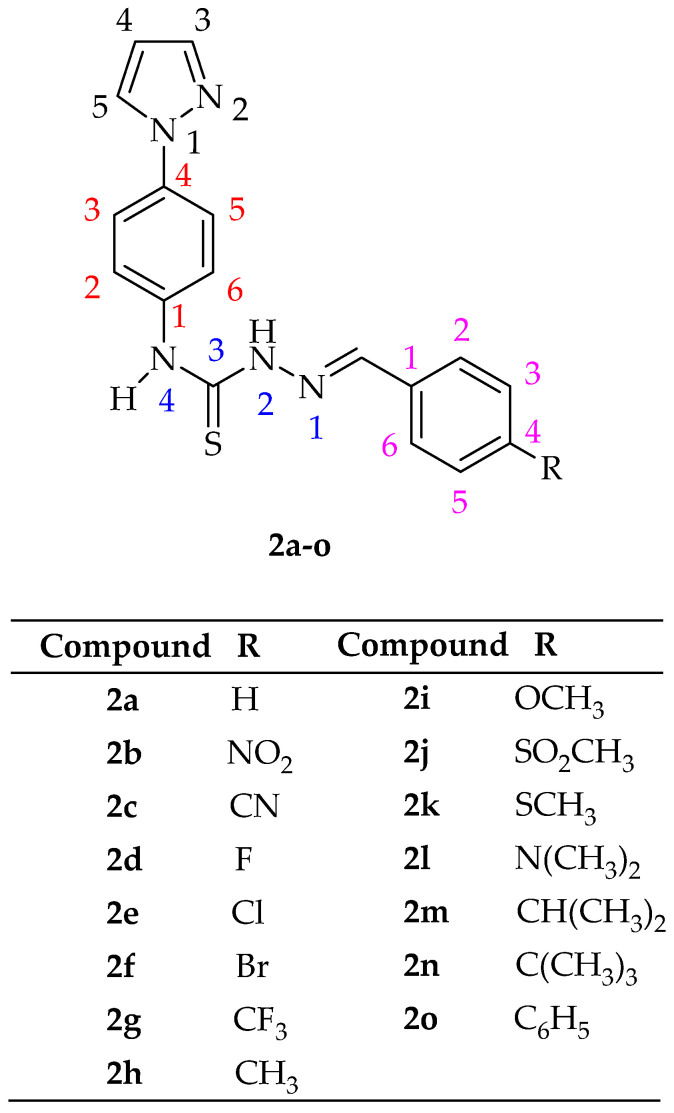
The diagram for NMR data of compounds **2a**–**o**.

In the ^1^H NMR spectra of compounds **2a**–**o**, both NH protons of thiosemicarbazone moiety were determined as singlet peaks at 10.03–10.36 ppm and 11.69–12.11 ppm. The characteristic azomethine (-CH=N-) protons appeared as a singlet at 8.22–8.51 ppm indicating, the formation of the thiosemicarbazone moiety of the final compounds. In their ^13^C NMR spectra, the characteristic carbons at 175.61–176.52 ppm and 140.80–144.65 ppm belonging to thiocarbonyl and azomethine groups, respectively, confirmed the formation of the thiosemicarbazone moiety of the final compounds. Moreover, the C_3_, C_4_ and C_5_ carbons of the pyrazole scaffold of the final compounds gave rise to the peaks at 140.40–141.36 ppm, 107.67–108.28 ppm and 121.60–131.24 ppm, respectively (Figure 4). Finally, the HRMS data of all compounds coincided with their molecular formulas.

### 2.2. Biological Activity

All synthesized compounds (**2a**–**o**) were screened for their in vitro DPP-4 inhibitory potencies at 100 µM concentration using a fluorescence-based assay (Table 1). Non-substituted compound **2a** caused the lowest DPP-4 inhibition (20.67 ± 1.82%), whilst bromo-substituted compound **2f** showed DPP-4 inhibition (98.20 ± 0.89%) even more than sitagliptin. Compounds **2f**, **2g**, **2i**, **2k**, and **2o**, which exhibited DPP-4 inhibition above 60%, were further analyzed for the determination of their half-maximal inhibitory concentration (IC_50_) values (Table 2).

Bromo-substituted compound **2f** was the most potent DPP-4 inhibitor in this series with an IC_50_ value of 1.266 ± 0.264 nM as indicated in Table 2. This compound was found to be more active than sitagliptin (IC_50_ = 4.380 ± 0.319 nM) indicating that the introduction of the bromo substituent into the *para* position of the benzylidene moiety gave rise to a substantial increase in DPP-4 inhibitory potency. The replacement of the bromo substituent of compound **2f** with the fluoro or the chloro substituent led to a significant drop in DPP-4 inhibitory activity.

Trifluoromethyl-substituted compound **2g** showed marked inhibitory effect on DPP-4 with an IC_50_ value of 4.775 ± 0.296 nM, similar to sitagliptin. It can be concluded that its DPP-4 inhibitory potency results from the presence of the trifluoromethyl group at the *para* position of the benzylidene moiety. Biphenyl-substituted compound **2o** and thiomethyl-substituted compound **2k** displayed moderate inhibitory effects on DPP-4 with IC_50_ values of 18.061 ± 0.311 nM and 22.671 ± 0.301 nM, respectively. Compound **2i** bearing a methoxy group showed DPP-4 inhibitory potency with an IC_50_ value of 43.312 ± 0.372 nM.

In an attempt to assess whether the compounds were toxic or non-toxic to normal cells, compounds **2a**–**o** were investigated for their cytotoxic effects on NIH/3T3 mouse embryonic fibroblast (normal) cells by means of MTT assay. All derivatives showed cytotoxicity towards the NIH/3T3 cell line with IC_50_ values higher than 500 µM. It can be concluded that they possess favourable safety profiles.

### 2.3. In Silico Studies

**Figure 5:**
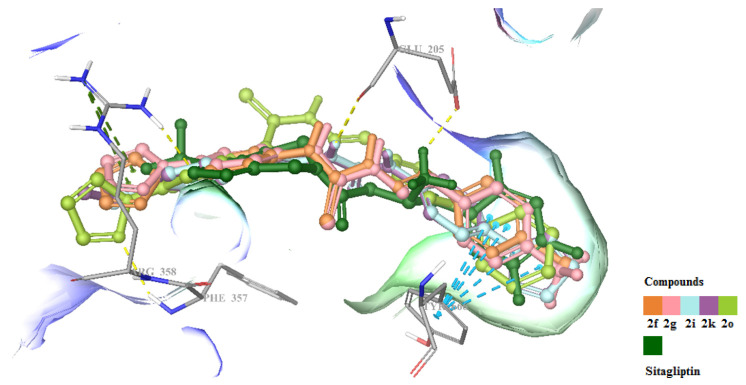
Docking poses of compounds **2f**, **2g**, **2i**, **2k** and **2o** along with sitagliptin in the active site of DPP-4 (Blue and green dashes: π-π interactions, yellow dashes: hydrogen bonding).

**Figure 6:**
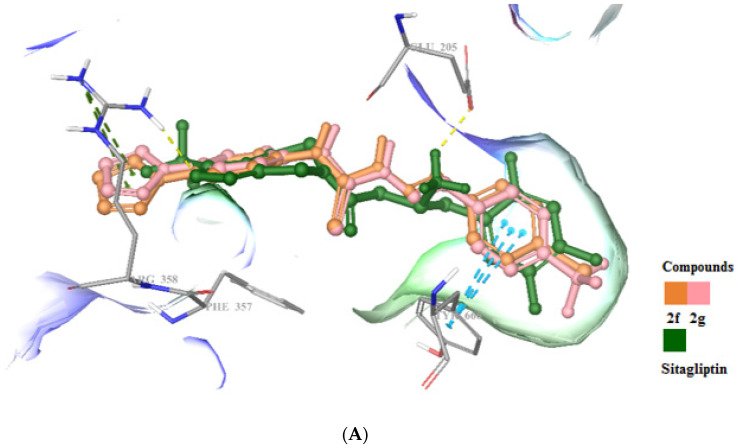

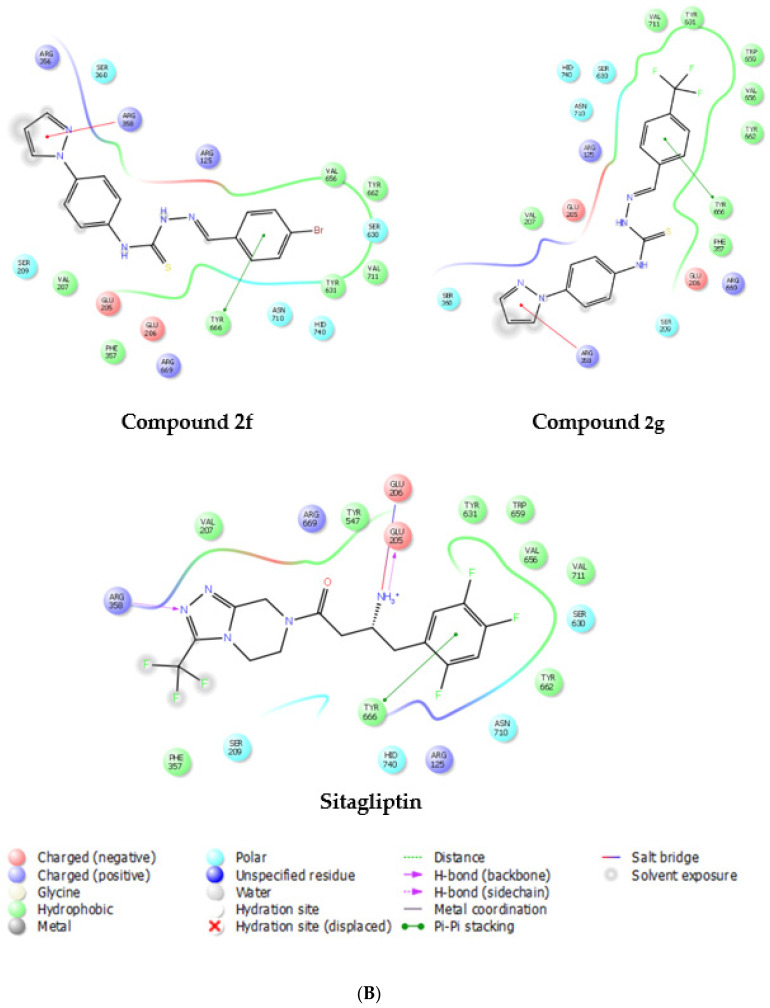
Docking poses of compounds **2f** and **2g** along with sitagliptin (**A**) and docking interactions of compounds **2f** and **2g** (**B**) in the active site of DPP-4 (Blue and green dashes: π-π interactions, yellow dashes: hydrogen bonding).

Molecular docking studies were conducted for compounds **2f**, **2g**, **2i**, **2k**, and **2o** in the active site of DPP-4 (PDB ID: 4FFW) compared to sitagliptin as they were defined as the most potent DPP-4 inhibitors based on the in vitro enzyme studies. The results of molecular docking indicated that substitutions on the thiosemicarbazone framework directly affected the proper interactions. In general, all these compounds formed cation-π interactions with Arg358 and Tyr666, which are key components in specific S1 and S2 pockets of DDP-4 [8] through pyrazole moiety and substituents at the *para* position of the phenyl ring (Figure 5). The bromo and the trifluoromethyl substitutions played major roles in the high activity of compounds **2f** and **2g** associated with their strong π-π interactions with Arg358 (Figure 6). Sitagliptin also presented the same strong interactions with Arg358 and Tyr666, whereas it also revealed hydrogen bonding and salt bridge formation with Glu205 and Glu206, which hampers its high DPP-4 inhibition. Compounds **2i** and **2k**, which were found less active DPP-4 inhibitors related to in vitro studies compared to compounds **2f**, **2g**, and **2o**, also formed hydrogen bonding with Glu205. These results were found totally coherent with in vitro studies.

Some Absorption, Distribution, Metabolism, and Excretion (ADME) properties including predicted octanol/water partition coefficient (QPlogPo/*w*), the conformation-independent predicted aqueous solubility (CIQPlogS), brain/blood partition coefficient (QPlogBB), human serum albumin binding (QPlogKhsa), and predicted human oral absorption were investigated in silico. The results given in Table 3 were found within the acceptable range based on the specified parameters including their predicted human oral absorption rates making these compounds appropriate drug candidates. Besides, compounds **2a**–**o** had no more than one violation of the certain criteria of Lipinski’s rule of five and Jorgensen’s rule of three.

## 3. Materials and Methods

### 3.1. Chemistry

All reagents purchased from commercial suppliers were used without further purification. The Electrothermal IA9200 digital melting point apparatus (Staffordshire, UK) was used to determine the melting points (M.p.) of the compounds. IR spectra were recorded on an IRPrestige-21 Fourier Transform Infrared spectrophotometer (Shimadzu, Tokyo, Japan). ^1^H NMR and ^13^C NMR spectra were recorded on Bruker 400 MHz and 500 MHz spectrometers (Bruker, Billerica, MA, USA). HRMS spectra were recorded on a Shimadzu LCMS-IT-TOF system (Shimadzu, Kyoto, Japan).

#### 3.1.1. General Procedure for the Synthesis of the Compounds

##### [4-(1*H*-Pyrazol-1-yl)phenyl]thiosemicarbazide (**1**)

A mixture of 4-(1*H*-pyrazol-1-yl)phenyl isothiocyanate (0.1 mol) and hydrazine hydrate (0.2 mol) in ethanol (30 mL) was stirred at room temperature for 4 h and then filtered. The residue was crystallized from ethanol [36].

##### [4-(1*H*-Pyrazol-1-yl)phenyl]-1-(arylidene)thiosemicarbazide (**2a**–**o**)

A mixture of 4-[4-(1*H*-pyrazol-1-yl)phenyl]thiosemicarbazide (**1**) (0.01 mol) and aromatic aldehyde (0.01 mol) was refluxed in ethanol for 8 h, filtered and crystallized from ethanol [36].

##### [4-(1*H*-Pyrazol-1-yl)phenyl]-1-(benzylidene)thiosemicarbazide (**2a**)

Off-white powder. M.p.: 218–220 °C. Yield: 75%.

IR ν_max_ (cm^−1^): 3298.28 (N-H stretching), 3149.76, 3116.97 (Aromatic C-H stretching), 2991.59, 2941.44 (Aliphatic C-H stretching), 1597.06, 1541.12, 1523.76, 1502.55 (N-H bending, C=N and C=C stretching), 1392.61, 1336.67, 1269.16, 1199.72, 1122.57, 1053.13, 1029.99 (C-H bending, C=S and C-N stretching, aromatic C-H in plane bending), 993.55, 829.39, 771.53, 758.02, 725.23, 688.59 (Aromatic C-H out of plane bending and C-S stretching) (Supplementary Material Figure S1).

^1^H NMR (400 MHz, DMSO-*d_6_*): 6.54 (s, 1H), 7.43–7.45 (m, 3H), 7.74 (d, *J* = 8.80 Hz, 3H), 7.85 (d, *J* = 8.40 Hz, 2H), 7.91–7.93 (m, 2H), 8.22 (s, 1H), 8.47 (s, 1H), 10.18 (s, 1H), 11.89 (s, 1H) (Supplementary Material Figures S2–S4).

^13^C NMR (100 MHz, DMSO-*d_6_*): 108.28 (CH), 117.96 (2CH), 126.60 (2CH), 127.54 (CH), 127.56 (2CH), 128.54 (2CH), 129.98 (CH), 133.91 (C), 136.84 (C), 137.06 (C), 140.76 (CH), 143.04 (CH), 176.04 (C) (Supplementary Material Figure S5).

HRMS (ESI) (*m*/*z*) [M + H]^+^ calcd. for C_17_H_15_N_5_S: 322.1121, found: 322.1122 (Supplementary Material Figure S6).

##### [4-(1*H*-Pyrazol-1-yl)phenyl]-1-(4-nitrobenzylidene)thiosemicarbazide (**2b**)

Yellow powder. M.p.: 244–246 °C. Yield: 90%.

IR ν_max_ (cm^−1^): 3313.71 (N-H stretching), 3149.76, 3099.61, 3049.46 (Aromatic C-H stretching), 2916.37, 2835.36 (Aliphatic C-H stretching), 1581.63, 1537.27, 1510.26, 1483.26 (N-H bending, C=N and C=C stretching), 1433.11, 1409.96, 1394.53, 1330.88, 1284.59, 1253.73, 1168.86, 1101.35, 1031.92 (C-H bending, NO_2_, C=S and C-N stretching, aromatic C-H in plane bending), 935.48, 873.75, 831.32, 746.45, 688.59, 659.66 (Aromatic C-H out of plane bending and C-S stretching) (Supplementary Material Figure S7).

^1^H NMR (400 MHz, DMSO-*d_6_*): 6.52 (s, 1H), 7.68 (d, *J* = 8.40 Hz, 2H), 7.73 (s, 1H), 7.83 (d, *J* = 8.40 Hz, 2H), 8.17 (d, *J* = 8.40 Hz, 3H), 8.23 (d, *J* = 8.40 Hz, 2H), 8.46 (s, 1H), 10.33 (s, 1H), 12.10 (s, 1H) (Supplementary Material Figures S8–S10).

^13^C NMR (100 MHz, DMSO-*d_6_*): 107.70 (CH), 117.99 (2CH), 123.64 (2CH), 126.89 (2CH), 127.57 (CH), 128.39 (2CH), 136.88 (C), 137.05 (C), 140.27 (C), 140.40 (CH), 140.80 (CH), 147.67 (C), 176.52 (C, C=S) (Supplementary Material Figure S11).

HRMS (ESI) (*m*/*z*) [M + H]^+^ calcd. for C_17_H_14_N_6_O_2_S: 367.0972, found: 367.0984) (Supplementary Material Figure S12).

##### [4-(1*H*-Pyrazol-1-yl)phenyl]-1-(4-cyanobenzylidene)thiosemicarbazide (**2c**)

Green powder. M.p.: 226–229 °C. Yield: 88%.

IR ν_max_ (cm^−1^): 3313.07 (N-H stretching), 3136.25 (Aromatic C-H stretching), 2981.95, 2848.86 (Aliphatic C-H stretching), 2218.14 (C≡N stretching), 1622.13, 1598.99, 1537.27, 1525.69, 1500.62 (N-H bending, C=N and C=C stretching), 1435.04, 1394.53, 1332.81, 1276.88, 1207.44, 1168.86, 1082.07, 1041.56, 1029.99 (C-H bending, C=S and C-N stretching, aromatic C-H in plane bending), 933.55, 867.97, 831.32, 777.31, 750.31, 671.23 (Aromatic C-H out of plane bending and C-S stretching) (Supplementary Material Figure S13).

^1^H NMR (400 MHz, DMSO-*d_6_*): 6.55 (s, 1H), 7.67 (d, *J* = 8.80 Hz, 3H), 7.75–7.90 (m, 4H), 8.14 (d, *J* = 8.80 Hz, 2H), 8.20 (s, 1H), 8.51 (s, 1H), 10.35 (s, 1H), 12.10 (s, 1H) (Supplementary Material Figures S14–S16).

^13^C NMR (100 MHz, DMSO-*d_6_*): 107.79 (CH), 111.70 (C), 117.99 (2CH), 118.74 (C), 127.05 (2CH), 127.66 (CH), 128.14 (2CH), 132.46 (2CH), 136.92 (C), 137.06 (C), 138.55 (C), 140.80 (CH), 140.88 (CH), 176.46 (C) (Supplementary Material Figure S17).

HRMS (ESI) (*m*/*z*) [M + H]^+^ calcd. for C_18_H_14_N_6_S: 347.1073, found: 347.1076 (Supplementary Material Figure S18).

##### [4-(1*H*-Pyrazol-1-yl)phenyl]-1-(4-fluorobenzylidene)thiosemicarbazide (**2d**)

Beige powder. M.p.: 200–204 °C. Yield: 80%.

IR ν_max_ (cm^−1^): 3282.84 (N-H stretching), 3142.04 (Aromatic C-H stretching), 2983.88, 2918.30 (Aliphatic C-H stretching), 1600.92, 1543.05, 1523.76, 1504.48 (N-H bending, C=N and C=C stretching), 1415.75, 1392.61, 1317.38, 1253.73, 1230.58, 1188.15, 1155.36, 1118.71, 1070.49, 1049.28, 1012.63 (C-H bending, C=S and C-N stretching, aromatic C-H in plane bending), 935.48, 871.82, 829.39, 796.60 (Aromatic C-H out of plane bending and C-S stretching) (Supplementary Material Figure S19).

^1^H NMR (400 MHz, DMSO-*d_6_*): 6.53 (s, 1H), 7.27 (t, *J* = 8.40 Hz, 8.80 Hz, 17.20 Hz, 2H), 7.73 (t, *J* = 8.80 Hz, 17.60 Hz, 3H), 7.85 (d, *J* = 8.80 Hz, 2H), 7.94–8.02 (m, 2H), 8.19 (s, 1H), 8.48 (s, 1H), 10.23 (s, 1H), 11.93 (s, 1H) (Supplementary Material Figures S20–S22).

^13^C NMR (100 MHz, DMSO-*d_6_*): 107.77 (CH), 115.66 (2CH, d, *J* = 21.80 Hz), 118.01 (2CH), 126.84 (2CH), 127.61 (CH), 129.87 (2CH, d, *J* = 8.40 Hz), 130.60 (C), 136.93 (C), 137.10 (C), 140.85 (CH), 141.89 (CH), 161.93 (C), 176.08 (C) (Supplementary Material Figure S23).

HRMS (ESI) (*m*/*z*) [M + H]^+^ calcd. for C_17_H_14_FN_5_S: 340.1027, found: 340.1031 (Supplementary Material Figure S24).

##### [4-(1*H*-Pyrazol-1-yl)phenyl]-1-(4-chlorobenzylidene)thiosemicarbazide (**2e**)

Off-white powder. M.p.: 194–198 °C. Yield: 86%.

IR ν_max_ (cm^−1^): 3313.71 (N-H stretching), 3180.62, 3107.32, 3064.89, 3047.53 (Aromatic C-H stretching), 2995.45, 2941.44, 2920.23, 2848.86 (Aliphatic C-H stretching), 1593.20, 1523.76, 1481.33 (N-H bending, C=N and C=C stretching), 1433.11, 1400.32, 1390.68, 1315.45, 1280.73, 1255.66, 1182.36, 1168.86, 1082.07, 1041.56, 1010.70 (C-H bending, C=S and C-N stretching, aromatic C-H in plane bending), 931.62, 835.18, 813.96, 767.67, 732.95 (Aromatic C-H out of plane bending and C-S stretching) (Supplementary Material Figure S25).

^1^H NMR (400 MHz, DMSO-*d_6_*): 6.54 (s, 1H), 7.49 (d, *J* = 8.40 Hz, 2H), 7.72 (t, *J* = 9.20 Hz, 9.60 Hz, 18.80 Hz, 3H), 7.84 (d, *J* = 8.40 Hz, 2H), 7.96 (d, *J* = 8.40 Hz, 2H), 8.18 (s, 1H), 8.48 (s, 1H), 10.22 (s, 1H), 11.92 (s, 1H) (Supplementary Material Figures S26–S28).

^13^C NMR (100 MHz, DMSO-*d_6_*): 107.67 (CH), 117.95 (2CH), 126.71 (2CH), 127.54 (CH), 128.60 (2CH), 129.20 (2CH), 132.90 (C), 134.45 (C), 136.89 (C), 137.01 (C), 140.76 (CH), 141.61 (CH), 176.13 (C) (Supplementary Material Figure S29).

HRMS (ESI) (*m*/*z*) [M + H]^+^ calcd. for C_17_H_14_ClN_5_S: 356.0731, found: 356.0740 (Supplementary Material Figure S30).

##### [4-(1*H*-Pyrazol-1-yl)phenyl]-1-(4-bromobenzylidene)thiosemicarbazide (**2f**)

Beige powder. M.p.: 196–199 °C. Yield: 83%.

IR ν_max_ (cm^−1^): 3315.63, 3288.63 (N-H stretching), 3140.11 (Aromatic C-H stretching), 2987.74, 2954.95, 2916.37, 2848.86 (Aliphatic C-H stretching), 1600.92, 1587.42, 1537.27, 1479.40, 1462.04 (N-H bending, C=N and C=C stretching), 1396.46, 1317.38, 1286.52, 1253.73, 1182.36, 1107.14, 1064.71, 1043.49, 1006.84 (C-H bending, C=S and C-N stretching, aromatic C-H in plane bending), 929.69, 835.18, 817.82, 767.67, 732.95 (Aromatic C-H out of plane bending and C-S stretching) (Supplementary Material Figure S31).

^1^H NMR (400 MHz, DMSO-*d_6_*): 6.55 (s, 1H), 7.57–7.78 (m, 5H), 7.81–7.90 (m, 4H), 8.07 and 8.14 (2s, 1H), 8.50 (s, 1H), 10.24 (s, 1H), 11.94 (s, 1H) (Supplementary Material Figures S32–S35).

^13^C NMR (100 MHz, DMSO-*d_6_*): 107.77 (CH), 117.96 (2CH), 123.30 (C), 126.89 (2CH), 127.64 (CH), 129.38 (CH), 129.51 (CH), 131.58 (CH), 131.61 (CH), 131.99 (C), 136.94 (C), 137.03 (C), 140.85 (CH), 141.73 (CH), 176.15 (C) (Supplementary Material Figure S36).

HRMS (ESI) (*m*/*z*) [M + H]^+^ calcd. for C_17_H_14_BrN_5_S: 400.0226, found: 400.0238 (Supplementary Material Figure S37).

##### [4-(1*H*-Pyrazol-1-yl)phenyl]-1-(4-trifluoromethylbenzylidene)thiosemicarbazide (**2g**)

White powder. M.p.: 222–225 °C. Yield: 79%.

IR ν_max_ (cm^−1^): 3315.63 (N-H stretching), 3182.55, 3113.11, 3082.25 (Aromatic C-H stretching), 2918.30, 2848.86 (Aliphatic C-H stretching), 1602.85, 1529.55, 1487.12, 1454.33 (N-H bending, C=N and C=C stretching), 1433.11, 1392.61, 1323.17, 1284.59, 1259.52, 1180.44, 1155.36, 1095.57, 1064.71, 1029.99, 1012.63 (C-H bending, C=S and C-N stretching, aromatic C-H in plane bending), 933.55, 831.32, 804.32, 736.81 (Aromatic C-H out of plane bending and C-S stretching) (Supplementary Material Figure S38).

^1^H NMR (400 MHz, DMSO-*d_6_*): 6.55 (s, 1H), 7.70 (d, *J* = 8.80 Hz, 2H), 7.75–7.79 (m, 3H), 7.86 (d, *J* = 8.80 Hz, 2H), 8.16 (d, *J* = 8.00 Hz, 2H), 8.25 (s, 1H), 8.50 (s, 1H), 10.34 (s, 1H), 12.09 (s, 1H) (Supplementary Material Figures S39–S41).

^13^C NMR (100 MHz, DMSO-*d_6_*): 107.77 (CH), 118.01 (2CH), 125.41 (C), 127.02 (2CH), 127.64 (2CH), 128.16 (2CH), 129.43 (CH), 129.74 (C), 136.99 (C), 137.06 (C), 138.04 (C), 140.87 (CH), 141.15 (CH), 176.46 (C) (Supplementary Material Figure S42).

HRMS (ESI) (*m*/*z*) [M + H]^+^ calcd. for C_18_H_14_F_3_N_5_S: 390.0995, found: 390.0997 (Supplementary Material Figure S43).

##### [4-(1*H*-Pyrazol-1-yl)phenyl]-1-(4-methylbenzylidene)thiosemicarbazide (**2h**)

White powder. M.p.: 192–194 °C. Yield: 76%.

IR ν_max_ (cm^−1^): 3302.13 (N-H stretching), 3157.47, 3026.31 (Aromatic C-H stretching), 2991.59, 2916.37 (Aliphatic C-H stretching), 1598.99, 1556.55, 1519.91, 1504.48 (N-H bending, C=N and C=C stretching), 1433.11, 1400.32, 1325.10, 1271.09, 1192.01, 1118.71, 1070.49, 1028.06 (C-H bending, C=S and C-N stretching, aromatic C-H in plane bending), 933.55, 871.82, 829.39, 808.17, 736.81, 686.66 (Aromatic C-H out of plane bending and C-S stretching) (Supplementary Material Figure S44).

^1^H NMR (400 MHz, DMSO-*d_6_*): 2.34 (s, 3H), 6.55 (s, 1H), 7.25 (d, *J* = 8.00 Hz, 2H), 7.72–7.85 (m, 7H), 8.16 (s, 1H), 8.50 (s, 1H), 10.16 (s, 1H), 11.85 (s, 1H) (Supplementary Material Figures S45–S47).

^13^C NMR (100 MHz, DMSO-*d_6_*): 21.06 (CH_3_), 107.75 (CH), 117.94 (2CH), 126.73 (2CH), 127.63 (2CH), 129.25 (2CH), 131.24 (CH), 136.84 (C), 137.12 (C), 139.92 (2C), 140.83 (CH), 143.18 (CH), 175.86 (C) (Supplementary Material Figure S48).

HRMS (ESI) (*m*/*z*) [M + H]^+^ calcd. for C_18_H_17_N_5_S: 336.1277, found: 336.1282 (Supplementary Material Figure S49).

##### [4-(1*H*-Pyrazol-1-yl)phenyl]-1-(4-methoxybenzylidene)thiosemicarbazide (**2i**)

Beige powder. M.p.: 190–192 °C. Yield: 78%.

IR ν_max_ (cm^−1^): 3304.06 (N-H stretching), 3182.55, 3124.68 (Aromatic C-H stretching), 2999.31, 2918.30, 2848.86 (Aliphatic C-H stretching), 1606.70, 1597.06, 1543.05, 1523.76, 1504.48, 1481.33 (N-H bending, C=N and C=C stretching), 1388.75, 1334.74, 1303.88, 1253.73, 1184.29, 1168.86, 1120.64, 1093.64, 1024.20 (C-H bending, C=S, C-O and C-N stretching, aromatic C-H in plane bending), 931.62, 833.25, 786.96, 758.02, 665.44 (Aromatic C-H out of plane bending and C-S stretching) (Supplementary Material Figure S50).

^1^H NMR (400 MHz, DMSO-*d_6_*): 3.81 (s, 3H), 6.54 (s, 1H), 6.99 (d, *J* = 8.40 Hz, 2H), 7.67–7.88 (m, 7H), 8.14 (s, 1H), 8.48 (s, 1H), 10.12 (s, 1H), 11.79 (s, 1H) (Supplementary Material Figures S51–S53).

^13^C NMR (100 MHz, DMSO-*d_6_*): 58.60 (CH_3_), 107.74 (CH), 114.13 (CH), 117.94 (2CH), 126.51 (CH), 126.64 (2CH), 127.57 (CH), 127.63 (CH), 129.12 (CH), 129.32 (C), 136.79 (C), 137.17 (C), 140.82 (CH), 143.04 (CH), 160.88 (C), 175.61 (C) (Supplementary Material Figure S54).

HRMS (ESI) (*m*/*z*) [M + H]^+^ calcd. for C_18_H_17_N_5_OS: 352.1227, found: 352.1229 (Supplementary Material Figure S55).

##### [4-(1*H*-Pyrazol-1-yl)phenyl]-1-(4-methylsulfonylbenzylidene)thiosemicarbazide (**2j**)

Buff powder. M.p.: 230–234 °C. Yield: 82%.

IR ν_max_ (cm^−1^): 3344.57 (N-H stretching), 3149.76, 3124.68 (Aromatic C-H stretching), 2995.45, 2916.37, 2848.86 (Aliphatic C-H stretching), 1598.99, 1525.69, 1502.55 (N-H bending, C=N and C=C stretching), 1433.11, 1390.68, 1332.81, 1303.88, 1288.45, 1269.16, 1193.94, 1138.00, 1080.14, 1043.49 (C-H bending, C=S and C-N stretching, aromatic C-H in plane bending), 968.27, 931.62, 869.90, 833.25, 823.60, 779.24 (Aromatic C-H out of plane bending and C-S stretching) (Supplementary Material Figure S56).

^1^H NMR (400 MHz, DMSO-*d_6_*): 3.27 (s, 3H), 6.55 (s, 1H), 7.69 (d, *J* = 9.20 Hz, 2H), 7.76 (s, 1H), 7.86 (d, *J* = 8.80 Hz, 2H), 7.97 (d, *J* = 8.80 Hz, 2H), 8.20 (d, *J* = 8.40 Hz, 2H), 8.25 (s, 1H), 8.50 (s, 1H), 10.36 (s, 1H), 12.11 (s, 1H) (Supplementary Material Figures S57–S59).

^13^C NMR (100 MHz, DMSO-*d_6_*): 43.42 (CH_3_), 107.82 (CH), 118.03 (2CH), 127.11 (2CH), 127.20 (CH), 127.66 (2CH), 128.20 (2CH), 129.16 (C), 136.95 (C), 137.10 (C), 138.90 (C), 140.90 (CH), 141.24 (CH), 176.50 (C) (Supplementary Material Figure S60).

HRMS (ESI) (*m*/*z*) [M + H]^+^ calcd. for C_18_H_17_N_5_O_2_S_2_: 400.0896, found: 400.0902 (Supplementary Material Figure S61).

##### [4-(1*H*-Pyrazol-1-yl)phenyl]-1-(4-methylthiobenzylidene)thiosemicarbazide (**2k**)

Pale yellow powder. M.p.: 196–198 °C. Yield: 84%.

IR ν_max_ (cm^−1^): 3346.50 (N-H stretching), 3140.11, 3041.74 (Aromatic C-H stretching), 2983.88, 2912.51 (Aliphatic C-H stretching), 1595.13, 1541.12, 1523.76, 1490.97 (N-H bending, C=N and C=C stretching), 1435.04, 1400.32, 1332.81, 1273.02, 1197.79, 1120.64, 1087.85, 1039.63 (C-H bending, C=S and C-N stretching, aromatic C-H in plane bending), 933.55, 866.04, 831.32, 804.32, 769.60, 723.31 (Aromatic C-H out of plane bending and C-S stretching) (Supplementary Material Figure S62).

^1^H NMR (500 MHz, DMSO-*d_6_*): 2.49 (s, 3H), 6.53 (s, 1H), 7.28 (d, *J* = 8.40 Hz, 2H), 7.69–7.74 (m, 3H), 7.82-7.85 (m, 4H), 8.13 (s, 1H), 8.48 (s, 1H), 10.16 (s, 1H), 11.85 (s, 1H) (Supplementary Material Figures S63–S65).

^13^C NMR (125 MHz, DMSO-*d_6_*): 14.74 (CH_3_), 108.27 (CH), 118.27 (2CH), 125.90 (CH), 127.27 (2CH), 128.14 (2CH), 128.56 (2CH), 130.89 (C), 137.35 (C), 137.61 (C), 141.34 (CH), 141.50 (CH), 143.17 (C), 176.30 (C) (Supplementary Material Figure S66).

HRMS (ESI) (*m*/*z*) [M + H]^+^ calcd. for C_18_H_17_N_5_S_2_: 368.0998, found: 368.1003 (Supplementary Material Figure S67).

##### [4-(1*H*-Pyrazol-1-yl)phenyl]-1-(4-dimethylaminobenzylidene)thiosemicarbazide (**2l**)

Pale brown powder. M.p.: 210–213 °C. Yield: 85%.

IR ν_max_ (cm^−1^): 3115.04, 3059.10 (Aromatic C-H stretching), 2974.23, 2895.15, 2806.43 (Aliphatic C-H stretching), 1606.70, 1543.05, 1521.84, 1510.26, 1444.68 (N-H bending, C=N and C=C stretching), 1394.53, 1357.89, 1330.88, 1267.23, 1215.15, 1170.79, 1118.71, 1070.49, 1049.28, 1033.85 (C-H bending, C=S and C-N stretching, aromatic C-H in plane bending), 939.33, 846.75, 819.75, 750.31, 734.88 (Aromatic C-H out of plane bending and C-S stretching) (Supplementary Material Figure S68).

^1^H NMR (500 MHz, DMSO-*d_6_*): 2.97 (s, 6H), 6.55 (s, 1H), 6.73 (d, *J* = 7.08 Hz, 2H), 7.72 (d, *J* = 7.00 Hz, 2H), 7.75 (d, *J* = 7.08 Hz, 2H), 7.84 (d, *J* = 7.08 Hz, 3H), 8.08 (s, 1H), 8.50 (s, 1H), 10.03 (s, 1H), 11.69 (s, 1H) (Supplementary Material Figures S69–72).

^13^C NMR (125 MHz, DMSO-*d_6_*): 40.33 (2CH_3_), 108.23 (CH), 112.09 (2CH), 118.43 (2CH), 121.60 (CH), 126.86 (2CH), 128.10 (2CH), 129.56 (C), 137.13 (C), 137.77 (C), 141.30 (CH), 144.65 (CH), 152.02 (C), 175.39 (C) (Supplementary Material Figure S73).

HRMS (ESI) (*m*/*z*) [M + H]^+^ calcd. for C_19_H_20_N_6_S: 365.1543, found: 365.1547 (Supplementary Material Figure S74).

##### [4-(1*H*-Pyrazol-1-yl)phenyl]-1-(4-isopropylbenzylidene)thiosemicarbazide (**2m**)

White powder. M.p.: 208–211 °C. Yield: 74%.

IR ν_max_ (cm^−1^): 3115.04, 3064.89 (Aromatic C-H stretching), 2970.38, 2954.95, 2868.15 (Aliphatic C-H stretching), 1606.70, 1525.69, 1504.48, 1462.04 (N-H bending, C=N and C=C stretching), 1394.53, 1332.81, 1265.30, 1209.37, 1197.79, 1078.21, 1049.28, 1012.63 (C-H bending, C=S and C-N stretching, aromatic C-H in plane bending), 937.40, 846.75, 833.25, 775.38, 754.17, 732.95, 659.66 (Aromatic C-H out of plane bending and C-S stretching) (Supplementary Material Figure S75).

^1^H NMR (500 MHz, DMSO-*d_6_*): 1.22 (d, *J* = 5.52 Hz, 6H), 2.89–2.95 (m, 1H), 6.56 (s, 1H), 7.31 (d, *J* = 6.56 Hz, 2H), 7.72 (d, *J* = 7.08 Hz, 2H), 7.76 (s, 1H), 7.83–7.86 (m, 4H), 8.17 (s, 1H), 8.51 (s, 1H), 10.17 (s, 1H), 11.88 (s, 1H) (Supplementary Material Figures S76–S79).

^13^C NMR (125 MHz, DMSO-*d_6_*): 24.14 (2CH_3_), 33.88 (CH), 108.26 (CH), 118.44 (2CH), 127.09 (CH), 127.21 (2CH), 128.13 (2CH), 128.25 (2CH), 132.15 (C), 137.34 (C), 137.60 (C), 141.33 (CH), 143.64 (CH), 151.23 (C), 176.35 (C) (Supplementary Material Figure S80).

HRMS (ESI) (*m*/*z*) [M + H]^+^ calcd. for C_20_H_21_N_5_S: 364.1590, found: 364.1593 (Supplementary Material Figure S81).

##### [4-(1*H*-Pyrazol-1-yl)phenyl]-1-(4-tert-butylbenzylidene)thiosemicarbazide (**2n**)

White powder. M.p.: 235–238 °C. Yield: 72%.

IR ν_max_ (cm^−1^): 3120.82 (Aromatic C-H stretching), 2960.73, 2954.95, 2866.22 (Aliphatic C-H stretching), 1606.70, 1537.27, 1525.69, 1506.41 (N-H bending, C=N and C=C stretching), 1394.53, 1334.74, 1265.30, 1213.23, 1122.57, 1080.14, 1049.28 (C-H bending, C=S and C-N stretching, aromatic C-H in plane bending), 937.40, 835.18, 759.95, 729.09, 661.58, 617.22 (Aromatic C-H out of plane bending and C-S stretching) (Supplementary Material Figure S82).

^1^H NMR (500 MHz, DMSO-*d_6_*): 1.31 (s, 9H), 6.56 (s, 1H), 7.46 (d, *J* = 6.68 Hz, 2H), 7.72 (d, *J* = 7.08 Hz, 2H), 7.76 (s, 1H), 7.84 (d, *J* = 6.94 Hz, 4H), 8.17 (s, 1H), 8.51 (s, 1H), 10.15 (s, 1H), 11.87 (s, 1H) (Supplementary Material Figures S83–S86).

^13^C NMR (125 MHz, DMSO-*d_6_*): 31.43 (3CH_3_), 35.08 (C), 108.26 (CH), 118.45 (2CH), 125.91 (CH), 127.15 (2CH), 127.97 (2CH), 128.14 (2CH), 131.76 (C), 137.33 (C), 137.60 (C), 141.33 (CH), 143.54 (CH), 153.40 (C), 176.34 (C) (Supplementary Material Figure S87).

HRMS (ESI) (*m*/*z*) [M + H]^+^ calcd. for C_21_H_23_N_5_S: 378.1747, found: 378.1749 (Supplementary Material Figure S88).

##### [4-(1*H*-Pyrazol-1-yl)phenyl]-1-(4-phenylbenzylidene)thiosemicarbazide (**2o**)

Yellow powder. M.p.: 210–212 °C. Yield: 83%.

IR ν_max_ (cm^−1^): 3311.78 (N-H stretching), 3132.40, 3030.17 (Aromatic C-H stretching), 2978.09 (Aliphatic C-H stretching), 1597.06, 1537.27, 1523.76, 1496.76 (N-H bending, C=N and C=C stretching), 1390.68, 1328.95, 1273.02, 1193.94, 1122.57, 1072.42, 1045.42 (C-H bending, C=S and C-N stretching, aromatic C-H in plane bending), 933.55, 867.97, 833.25, 761.88, 719.45, 688.59, 653.87 (Aromatic C-H out of plane bending and C-S stretching) (Supplementary Material Figure S89).

^1^H NMR (500 MHz, DMSO-*d_6_*): 6.56 (s, 1H), 7.39 (t, *J* = 7.10 Hz, 7.20 Hz, 14.30 Hz, 1H), 7.49 (t, *J* = 7.35 Hz, 7.45 Hz, 14.80 Hz, 2H), 7.75 (t, *J* = 8.75 Hz, 8.15 Hz, 16.90 Hz, 7H), 7.87 (d, *J* = 8.50 Hz, 2H), 8.03 (d, *J* = 7.95 Hz, 2H), 8.26 (s, 1H), 8.50 (s, 1H), 10.28 (s, 1H), 11.99 (s, 1H) (Supplementary Material Figures S90–S92).

^13^C NMR (125 MHz, DMSO-*d_6_*): 108.28 (CH), 118.49 (2CH), 127.19 (2CH), 127.31 (2CH), 127.33 (CH), 128.13 (2CH), 128.34 (CH), 128.78 (2CH), 129.49 (2CH), 133.62 (C), 137.41 (C), 137.63 (C), 139.85 (C), 141.36 (CH), 142.03 (C), 143.15 (CH), 176.51 (C) (Supplementary Material Figure S93).

HRMS (ESI) (*m*/*z*) [M + H]^+^ calcd. for C_23_H_19_N_5_S: 398.1434, found: 398.1737 (Supplementary Material Figure S94).

### 3.2. DPP-4 Inhibitor Screening Assay

DPP-4 Inhibitor Screening Assay Kit (Cayman Chemicals, Ann Arbor, MI, USA), a fluorescence-based method, is used to screen the DPP-4 inhibitory effects of compounds **2a**–**o** and sitagliptin (Cayman Chemicals, Ann Arbor, MI, USA) according to the manufacturer’s instructions. This assay kit allows DPP-4 measurement using Gly-Pro-Aminomethylcoumarin (AMC) as a fluorogenic substrate. The free AMC group is released through the cleavage of the peptide bond by DPP leading to fluorescence that can be examined using appropriate excitation and emission wavelengths. Briefly, the experiment was performed triplicate in a white flat bottom 96 well plate. Thirty microlitres of assay buffer, 10 µL of DPP-4 enzyme, 10 µL of dimethyl sulfoxide (DMSO), and 50 µL of DPP substrate were added to 100% initial activity wells. Forty microlitres of assay buffer, 10 µL of DMSO, and 50 µL of DPP substrate were added to background wells. Thirty microlitres of assay buffer, 10 µL of DPP-4 enzyme, 10 µL of sample, and 50 µL of DPP substrate were added to inhibitor wells. Sitagliptin was used as a positive control inhibitor. The plate was incubated for 30 min at 37 °C, and fluorescence measurements were analyzed using an excitation wavelength of 360/40 nm and an emission wavelength of 460/40 nm in a multi-mode plate reader (Synergy HTX S1LFA, BioTek Instruments, Winooski, VT, USA). All assays were performed in triplicate. The fluorescence of the background wells was subtracted from all other wells before the calculations. The per cent inhibition was determined by the following formula in equation 1:

Inhibition% = [(Initial activity − Inhibitor)/Initial Activity] × 100(1)

First, all compounds were studied at the same concentration (100 µM) with DPP-4 Inhibitor Screening Assay Kit for activity pre-screening results. After the elimination of the lower potency compounds, the effective compounds were tested at five different concentrations with 1:10 dilutions (10^−1^ to 10^−5^ µM) to determine the IC_50_ values.

### 3.3. Cytotoxicity

#### 3.3.1. Cell Culture

NIH/3T3 mouse embryonic fibroblast (normal) cells (ATCC^®^ CRL-1658™) were grown in RPMI 1640 medium (Sigma-Aldrich, St. Louis, MO, USA) supplemented with 2 mM L-glutamine (Gibco, Paisley, UK), 10% fetal bovine serum (Gibco, Paisley, UK) and 1% penicillin/streptomycin (Gibco, Paisley, UK) at 37 °C in a humidified incubator with a 5% CO_2_ atmosphere. Compounds **2a**–**o** were dissolved in DMSO (Sigma-Aldrich, St. Louis, MO, USA) and diluted to required concentrations with a fresh medium. The control group (solvent control) was prepared with a medium containing 0.1% DMSO.

#### 3.3.2. MTT Assay

MTT [(3-(4,5-dimethylthiazol-2-yl)-2,5-diphenyltetrazolium bromide] (Sigma-Aldrich, St. Louis, MO, USA) assay is based on the reduction of MTT by the mitochondrial dehydrogenase of intact cells to a purple formazan product [37]. Yellow MTT is reduced to purple formazan in the mitochondria of viable cells. This reduction takes place only when mitochondrial reductase enzymes are active, and therefore, the conversion can be directly related to the number of viable cells [38].

In order to determine IC_50_ concentrations of compounds **2a**–**o**, cell viability was determined by MTT assay. Briefly, cells were grown in 96-well plates at a density of 5 × 10^3^ cells per well and subjected to different concentrations (500, 250, 125, 62.5 and 31.25 µM) of the compounds. After 24 h incubation, the MTT solution was added to wells to reach a final concentration of 0.5 mg/mL. The cells were incubated for a further 4 h and the then-current medium was removed and 100 µL of DMSO solution was added. The absorbance values were measured at 540 nm using a Cytation 3 Cell Imaging Multi-Mode Reader (BioTek, Winooski, VT, USA). Cell survival rates were expressed as the percentage of the DMSO (0.1%) solvent control and IC_50_ concentrations were calculated according to the analysis result.

### 3.4. Statistical Analyses

All descriptive data were expressed as the mean±standard deviation. All statistical analysis was performed by using GraphPad Prism Software version 8.4.2. A multiple comparisons test was applied to compare the mean of each column with the mean of control column with the Dunnet test. Mean standard deviation was calculated for quantitative data. *p* values less than 0.05 (≤0.05) were considered to be statistically significant.

### 3.5. Molecular Docking Studies

The most active DPP-4 inhibitors in this series and sitagliptin were docked to the active site of DPP-4. Ligands were set to the physiological pH (pH = 7.4) at the protonation step and the crystal structure of DPP-4 was retrieved from Protein Data Bank server (PDB code: 4FFW). The structures of the compounds were submitted in the protein preparation module of Schrodinger’s Maestro molecular modelling package (Schrödinger Release 2016-2: Schrödinger, LLC, New York, NY, USA). In molecular docking simulations, Glide/XP docking protocols were applied for the prediction of topologies of the compounds in the active site of the target enzyme [36].

### 3.6. In Silico ADME Studies

The pharmacokinetic profiles of compounds **2a**–**o** were predicted using the QikProp module of Schrödinger’s Molecular modelling package (Schrödinger Release 2016-2: QikProp, Schrödinger, LLC., New York, NY, USA, 2016). According to Lipinski’s rule of five and Jorgensen’s rule of three, all compounds were evaluated for their drug-likeness and oral bioavailability.

## 4. Conclusions

In conclusion, new 4-[4-(1*H*-pyrazol-1-yl)phenyl]-1-arylidenethiosemicarbazides (**2a**–**o**) were designed as DPP-4 inhibitors and synthesized efficiently through a two-step procedure. Compounds **2a**–**o** were investigated for their in vitro DPP-4 inhibitory potencies using a fluorescence-based assay. According to the results, compound **2f** bearing the bromo substituent at the *para* position of the benzylidene moiety was found to possess the most pronounced inhibitory effect on DPP-4 in this series (IC_50_ = 1.266 ± 0.264 nM) when compared to sitagliptin (IC_50_ = 4.380 ± 0.319 nM). The cytotoxic effects of the compounds on NIH/3T3 mouse embryonic fibroblast (normal) cells were examined. The MTT assay pointed out the safety of compound **2f** as a promising DPP-4 inhibitor. Molecular docking studies pointed out that compound **2f** revealed high affinity to the active site of DPP-4 and established strong hydrophobic interactions with key residues very similar to sitagliptin. Based on the safety profile and good tolerance of current available DPP-4 inhibitors, according to both in vitro and in silico studies compound **2f** stands out as a potential antidiabetic agent showing DPP-4 inhibition.

## Figures and Tables

**Table 1:** The percentage inhibition values of compounds **2a**–**o** and sitagliptin for DPP-4.

Compound (100 µM)	R	DPP-4 Inhibition%
**2a**	H	20.67 ± 1.82
**2b**	NO_2_	35.54 ± 1.75
**2c**	CN	28.04 ± 1.63
**2d**	F	27.28 ± 2.03
**2e**	Cl	38.44 ± 2.14
2f *	Br	98.20 ± 0.89
2g *	CF_3_	62.82 ± 1.19
**2h**	CH_3_	53.16 ± 3.10
2i *	OCH_3_	77.18 ± 2.21
**2j**	SO_2_CH_3_	59.84 ± 1.13
2k *	SCH_3_	70.44 ± 1.03
**2l**	N(CH_3_)_2_	57.34 ± 1.04
**2m**	CH(CH_3_)_2_	47.43 ± 1.29
**2n**	C(CH_3_)_3_	28.64 ± 1.76
2o *	C_6_H_5_	75.72 ± 1.48
Sitagliptin *	-	90.38 ± 2.17

* These compounds were selected for further analysis to calculate the IC_50_ values. IC_50_ calculation was not performed for the concentrations higher than 100 µM for the results below 40% inhibition, and the results above 60% inhibition were analyzed for concentrations less than 100 µM indicated by an asterisk.

**Table 2:** IC_50_ values of compounds **2f**, **2g**, **2i**, **2k**, and **2o** for DPP-4 inhibition.

Compound	IC_50_ (nM)
**2f**	1.266 ± 0.264
**2g**	4.775 ± 0.296
**2i**	43.312 ± 0.372
**2k**	22.671 ± 0.301
**2o**	18.061 ± 0.311
**Sitagliptin**	4.380 ± 0.319

**Table 3:** Predicted Absorption, Distribution, Metabolism, and Excretion (ADME) properties of compounds **2a**–**o**.

Compound	QPlogPo/*w* *	QPlogBB *	CIQPlogS *	QPlogKhsa *	Human Oral Absorption% *
**2a**	4.07	−0.25	−4.73	0.33	100.00
**2b**	3.38	−1.40	−5.25	0.30	90.34
**2c**	3.34	−1.14	−5.67	0.19	94.39
**2d**	4.31	−0.14	−5.08	0.37	100.00
**2e**	4.56	−0.09	−5.41	0.44	100.00
**2f**	4.64	−0.08	−6.30	0.46	100.00
**2g**	5.04	0.07	−6.08	0.58	100.00
**2h**	4.38	−0.27	−5.00	0.48	100.00
**2i**	4.17	−0.33	−5.04	0.35	100.00
**2j**	2.86	−1.25	−4.77	−0.02	90.38
**2k**	4.69	−0.24	−5.45	0.50	100.00
**2l**	4.50	−0.37	−5.21	0.49	100.00
**2m**	5.03	−0.36	−5.56	0.72	100.00
**2n**	5.30	−0.35	−5.84	0.85	100.00
**2o**	5.67	−0.37	−6.50	0.89	100.00

* QPlogPo/*w*: Predicted octanol/water partition coefficient (−2.00 to 6.50), QPlogBB: brain/blood partition coefficient (−3.00 to 1.20), CIQPlogS: The conformation-independent predicted aqueous solubility (−6.50 to 0.50), QPlogKhsa: binding to human serum albumin (−1.50 to 1.50), Percent Human-Oral Absorption: human oral absorption on 0–100% scale (>80% is high, <25% is poor).

## Supplementary Materials

The following are available online. Figure S1: IR spectrum of compound **2a**, Figure S2: ^1^H NMR spectrum of compound **2a**, Figure S3: ^1^H NMR spectrum of compound **2a** with integral values, Figure S4: ^1^H NMR spectrum of compound **2a** (6–12 ppm), Figure S5: ^13^C NMR spectrum of compound **2a**, Figure S6: HRMS spectrum of compound **2a**, Figure S7: IR spectrum of compound **2b**, Figure S8: ^1^H NMR spectrum of compound **2b**, Figure S9: ^1^H NMR spectrum of compound **2b** with integral values, Figure S10: ^1^H NMR spectrum of compound **2b** (6–12 ppm), Figure S11: ^13^C NMR spectrum of compound **2b**, Figure S12: HRMS spectrum of compound **2b**, Figure S13: IR spectrum of compound **2c**, Figure S14: ^1^H NMR spectrum of compound **2c**, Figure S15: ^1^H NMR spectrum of compound **2c** with integral values, Figure S16: ^1^H NMR spectrum of compound **2c** (6–12 ppm), Figure S17: ^13^C NMR spectrum of compound **2c**, Figure S18: HRMS spectrum of compound **2c**, Figure S19: IR spectrum of compound **2d**, Figure S20: ^1^H NMR spectrum of compound **2d**, Figure S21: ^1^H NMR spectrum of compound **2d** with integral values, Figure S22: ^1^H NMR spectrum of compound **2d** (6–12 ppm), Figure S23: ^13^C NMR spectrum of compound **2d**, Figure S24: HRMS spectrum of compound **2d**, Figure S25: IR spectrum of compound **2e**, Figure S26: ^1^H NMR spectrum of compound **2e**, Figure S27: ^1^H NMR spectrum of compound **2e** with integral values, Figure S28: ^1^H NMR spectrum of compound **2e** (6–12 ppm), Figure S29: ^13^C NMR spectrum of compound **2e**, Figure S30: HRMS spectrum of compound **2e**, Figure S31: IR spectrum of compound **2f**, Figure S32: ^1^H NMR spectrum of compound **2f**, Figure S33: ^1^H NMR spectrum of compound **2f** with integral values, Figure S34: ^1^H NMR spectrum of compound **2f** (6.6–8.8 ppm), Figure S35: ^1^H NMR spectrum of compound **2f** (10.2–12.2 ppm), Figure S36: ^13^C NMR spectrum of compound **2f**, Figure S37: HRMS spectrum of compound **2f**, Figure S38: IR spectrum of compound **2g**, Figure S39: ^1^H NMR spectrum of compound **2g**, Figure S40: ^1^H NMR spectrum of compound **2g** with integral values, Figure S41: ^1^H NMR spectrum of compound **2g** (6–12 ppm), Figure S42: ^13^C NMR spectrum of compound **2g**, Figure S43: HRMS spectrum of compound **2g**, Figure S44: IR spectrum of compound **2h**, Figure S45: ^1^H NMR spectrum of compound **2h**, Figure S46: ^1^H NMR spectrum of compound **2h** with integral values, Figure S47: ^1^H NMR spectrum of compound **2h** (2.3–12 ppm), Figure S48: ^13^C NMR spectrum of compound **2h**, Figure S49: HRMS spectrum of compound **2h**, Figure S50: IR spectrum of compound **2i**, Figure S51: ^1^H NMR spectrum of compound **2i**, Figure S52: ^1^H NMR spectrum of compound **2i** with integral values, Figure S53: ^1^H NMR spectrum of compound **2i** (3.8–12 ppm), Figure S54: ^13^C NMR spectrum of compound **2i**, Figure S55: HRMS spectrum of compound **2i**, Figure S56: IR spectrum of compound **2j**, Figure S57: ^1^H NMR spectrum of compound **2j**, Figure S58: ^1^H NMR spectrum of compound **2j** with integral values, Figure S59: ^1^H NMR spectrum of compound **2j** (3.2–12 ppm), Figure S60: ^13^C NMR spectrum of compound **2j**, Figure S61: HRMS spectrum of compound **2j**, Figure S62: IR spectrum of compound **2k**, Figure S63: ^1^H NMR spectrum of compound **2k**, Figure S64: ^1^H NMR spectrum of compound **2k** with integral values, Figure S65: ^1^H NMR spectrum of compound **2k** (2.5–12 ppm), Figure S66: ^13^C NMR spectrum of compound **2k**, Figure S67: HRMS spectrum of compound **2k**, Figure S68: IR spectrum of compound **2l**, Figure S69: ^1^H NMR spectrum of compound **2l**, Figure S70: ^1^H NMR spectrum of compound **2l** with integral values, Figure S71: ^1^H NMR spectrum of compound **2l** (2.5–3.5 ppm), Figure S72: ^1^H NMR spectrum of compound **2l** (6.5–12 ppm), Figure S73: ^13^C NMR spectrum of compound **2l**, Figure S74: HRMS spectrum of compound **2l**, Figure S75: IR spectrum of compound **2m**, Figure S76: ^1^H NMR spectrum of compound **2m**, Figure S77: ^1^H NMR spectrum of compound **2m** with integral values, Figure S78: ^1^H NMR spectrum of compound **2m** (1–3.3 ppm), Figure S79: ^1^H NMR spectrum of compound **2m** (6.5–12 ppm), Figure S80: ^13^C NMR spectrum of compound **2m**, Figure S81: HRMS spectrum of compound **2m**, Figure S82: IR spectrum of compound **2n**, Figure S83: ^1^H NMR spectrum of compound **2n**, Figure S84: ^1^H NMR spectrum of compound **2n** with integral values, Figure S85: ^1^H NMR spectrum of compound **2n** (1–3.3 ppm), Figure S86: ^1^H NMR spectrum of compound **2n** (6.5–12 ppm), Figure S87: ^13^C NMR spectrum of compound **2n**, Figure S88: HRMS spectrum of compound **2n**, Figure S89: IR spectrum of compound **2o**, Figure S90: ^1^H NMR spectrum of compound **2o**, Figure S91: ^1^H NMR spectrum of compound **2o** with integral values, Figure S92: ^1^H NMR spectrum of compound **2o** (6.5–12 ppm), Figure S93: ^13^C NMR spectrum of compound **2o**, Figure S94: HRMS spectrum of compound **2o**.
