# Supplementary material for: Pyrazole Incorporated New Thiosemicarbazones: Design, Synthesis and Investigation of DPP-4 Inhibitory Effects
Source: Molecules. 2020 Oct 28;25(21):5003. doi: 10.3390/molecules25215003 (PMC7662656; doi:10.3390/molecules25215003)
Supplement: Supplementary file 1 [file molecules-25-05003-s001.pdf]

Supplementary Material

Article

Pyrazole Incorporated New Thiosemicarbazones: Design, Synthesis and Investigation of DPP-4 Inhibitory Effects

Belgin Sever [1], Hasan Soybir [1], Şennur Görgülü [2], Zerrin Cantürk [3] and Mehlika Dilek Altıntop [1,*]

[1] Department of Pharmaceutical Chemistry, Faculty of Pharmacy, Anadolu University, 26470 Eskişehir, Turkey; belginsever@anadolu.edu.tr (B.S.); hasan_soybir@hotmail.com (H.S.)
[2] Medicinal Plant, Drug and Scientific Research and Application Center, Anadolu University, 26470 Eskişehir, Turkey; sennur@gmail.com (Ş.G.)
[3] Department of Pharmaceutical Microbiology, Faculty of Pharmacy, Anadolu University, 26470 Eskişehir, Turkey; zkcanturk@anadolu.edu.tr (Z.C.)

* Correspondence: mdaltintop@anadolu.edu.tr (M.D.A.); Tel.: +90-222-335-0580.

Contents

Figure S1: IR spectrum of compound **2a**

Figure S2: ^{1}H NMR spectrum of compound **2a**

Figure S3: ^{1}H NMR spectrum of compound **2a** with integral values

Figure S4: ^{1}H NMR spectrum of compound **2a** (6-12 ppm)

Figure S5: ^{13}C NMR spectrum of compound **2a**

Figure S6: HRMS spectrum of compound **2a**

Figure S7: IR spectrum of compound **2b**

Figure S8: ^{1}H NMR spectrum of compound **2b**

Figure S9: ^{1}H NMR spectrum of compound **2b** with integral values

Figure S10: ^{1}H NMR spectrum of compound **2b** (6-12 ppm)

Figure S11: ^{13}C NMR spectrum of compound **2b**

Figure S12: HRMS spectrum of compound **2b**

Figure S13: IR spectrum of compound **2c**

Figure S14: ^{1}H NMR spectrum of compound **2c**

Figure S15: ^{1}H NMR spectrum of compound **2c** with integral values

Figure S16: ^{1}H NMR spectrum of compound **2c** (6-12 ppm)

Figure S17: ^{13}C NMR spectrum of compound **2c**

Figure S18: HRMS spectrum of compound **2c**

Figure S19: IR spectrum of compound **2d**

Figure S20: ^{1}H NMR spectrum of compound **2d**

Figure S21: ^{1}H NMR spectrum of compound **2d** with integral values

Figure S22: ^{1}H NMR spectrum of compound **2d** (6-12 ppm)

Figure S23: ^{13}C NMR spectrum of compound **2d**

Figure S24: HRMS spectrum of compound **2d**

Figure S25: IR spectrum of compound **2e**

Figure S26: ^{1}H NMR spectrum of compound **2e**

Figure S27: ^{1}H NMR spectrum of compound **2e** with integral values

Figure S28: ^{1}H NMR spectrum of compound **2e** (6-12 ppm)

Figure S29: ^{13}C NMR spectrum of compound **2e**

Figure S30: HRMS spectrum of compound **2e**

Figure S31: IR spectrum of compound **2f**

Figure S32: ^{1}H NMR spectrum of compound **2f**

Figure S33: ^{1}H NMR spectrum of compound **2f** with integral values

Figure S34: ^{1}H NMR spectrum of compound **2f** (6.6-8.8 ppm)

Figure S35: ^{1}H NMR spectrum of compound **2f** (10.2-12.2 ppm)

Figure S36: ^{13}C NMR spectrum of compound **2f**

Figure S37: HRMS spectrum of compound **2f**

Figure S38: IR spectrum of compound **2g**

Figure S39: ^{1}H NMR spectrum of compound **2g**

Figure S40: ^{1}H NMR spectrum of compound **2g** with integral values

Figure S41: ^{1}H NMR spectrum of compound **2g** (6-12 ppm)

Figure S42: ^{13}C NMR spectrum of compound **2g**

Figure S43: HRMS spectrum of compound **2g**

Figure S44: IR spectrum of compound **2h**

Figure S45: ^{1}H NMR spectrum of compound **2h**

Figure S46: ^{1}H NMR spectrum of compound **2h** with integral values

Figure S47: ^{1}H NMR spectrum of compound **2h** (2.3-12 ppm)

Figure S48: ^{13}C NMR spectrum of compound **2h**

Figure S49: HRMS spectrum of compound **2h**

Figure S50: IR spectrum of compound **2i**

Figure S51: ^{1}H NMR spectrum of compound **2i**

Figure S52: ^{1}H NMR spectrum of compound **2i** with integral values

Figure S53: ^{1}H NMR spectrum of compound **2i** (3.8-12 ppm)

Figure S54: ^{13}C NMR spectrum of compound **2i**

Figure S55: HRMS spectrum of compound **2i**

Figure S56: IR spectrum of compound **2j**

Figure S57: ^{1}H NMR spectrum of compound **2j**

Figure S58: ^{1}H NMR spectrum of compound **2j** with integral values

Figure S59: ^{1}H NMR spectrum of compound **2j** (3.2-12 ppm)

Figure S60: ^{13}C NMR spectrum of compound **2j**

Figure S61: HRMS spectrum of compound **2j**

Figure S62: IR spectrum of compound **2k**

Figure S63: ^{1}H NMR spectrum of compound **2k**

Figure S64: ^{1}H NMR spectrum of compound **2k** with integral values

Figure S65: ^{1}H NMR spectrum of compound **2k** (2.5-12 ppm)

Figure S66: ^{13}C NMR spectrum of compound **2k**

Figure S67: HRMS spectrum of compound **2k**

Figure S68: IR spectrum of compound **2l**

Figure S69: ^{1}H NMR spectrum of compound **2l**

Figure S70: ^{1}H NMR spectrum of compound **2l** with integral values

Figure S71: ^{1}H NMR spectrum of compound **2l** (2.5-3.5 ppm)

Figure S72: ^{1}H NMR spectrum of compound **2l** (6.5-12 ppm)

Figure S73: ^{13}C NMR spectrum of compound **2l**

Figure S74: HRMS spectrum of compound **2l**

Figure S75: IR spectrum of compound **2m**

Figure S76: ^{1}H NMR spectrum of compound **2m**

Figure S77: ^{1}H NMR spectrum of compound **2m** with integral values

Figure S78: ^{1}H NMR spectrum of compound **2m** (1-3.3 ppm)

Figure S79: ^{1}H NMR spectrum of compound **2m** (6.5-12 ppm)

Figure S80: ^{13}C NMR spectrum of compound **2m**

Figure S81: HRMS spectrum of compound **2m**

Figure S82: IR spectrum of compound **2n**

Figure S83: ^{1}H NMR spectrum of compound **2n**

Figure S84: ^{1}H NMR spectrum of compound **2n** with integral values

Figure S85: ^{1}H NMR spectrum of compound **2n** (1-3.3 ppm)

Figure S86: ^{1}H NMR spectrum of compound **2n** (6.5-12 ppm)

Figure S87: ^{13}C NMR spectrum of compound **2n**

Figure S88: HRMS spectrum of compound **2n**

Figure S89: IR spectrum of compound **2o**

Figure S90: ^{1}H NMR spectrum of compound **2o**

Figure S91: ^{1}H NMR spectrum of compound **2o** with integral values

Figure S92: ^{1}H NMR spectrum of compound **2o** (6.5-12 ppm)

Figure S93: ^{13}C NMR spectrum of compound **2o**

Figure S94: HRMS spectrum of compound **2o**

Figure S1: IR spectrum of compound **2a**

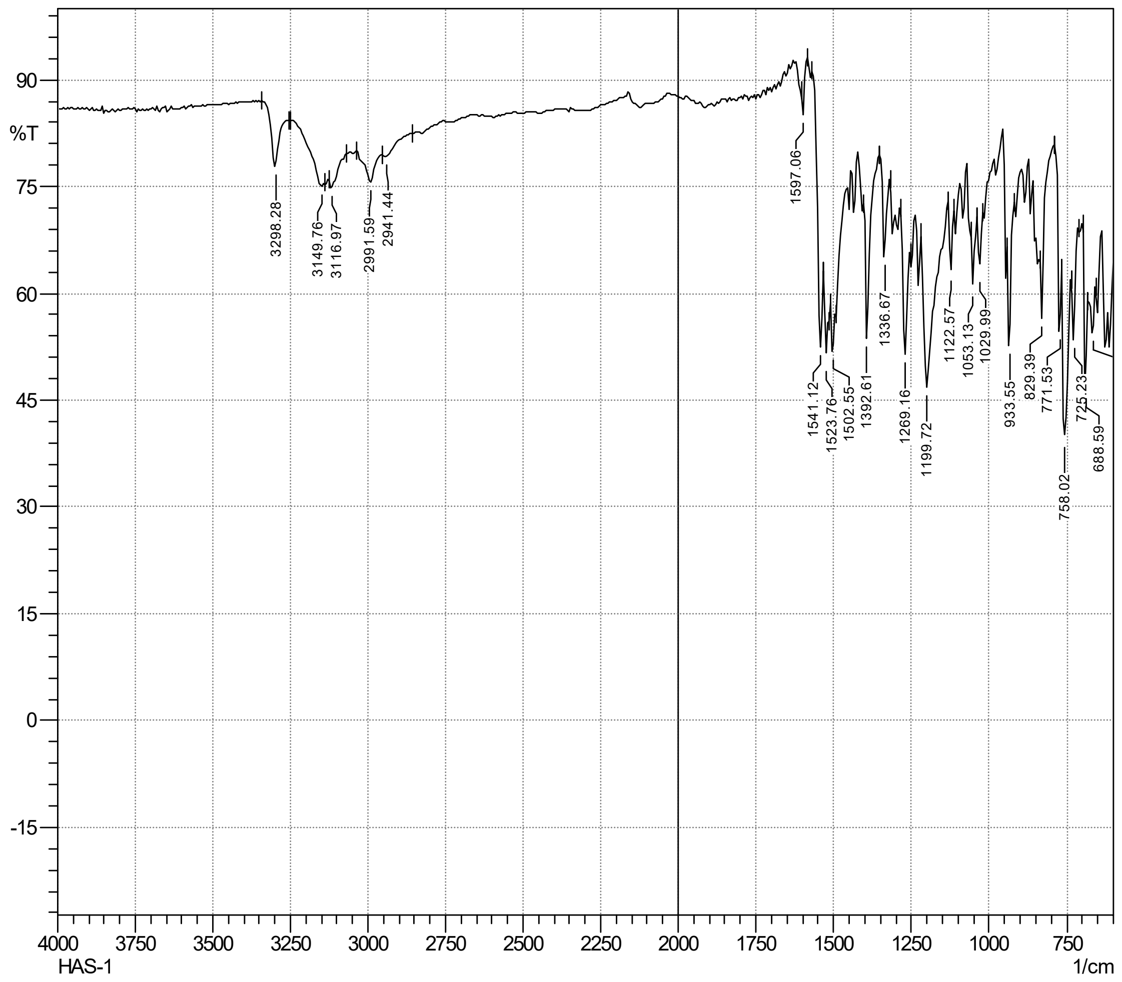

Figure S2: ^{1}H NMR spectrum of compound **2a**

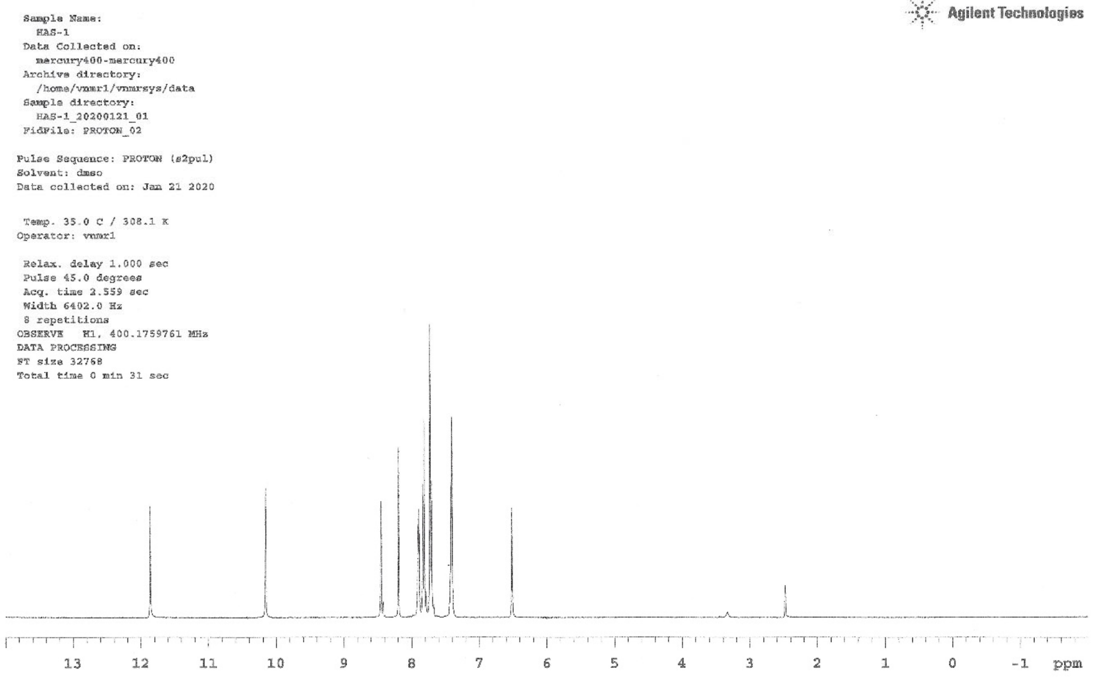

Figure S3: ^{1}H NMR spectrum of compound **2a** with integral values

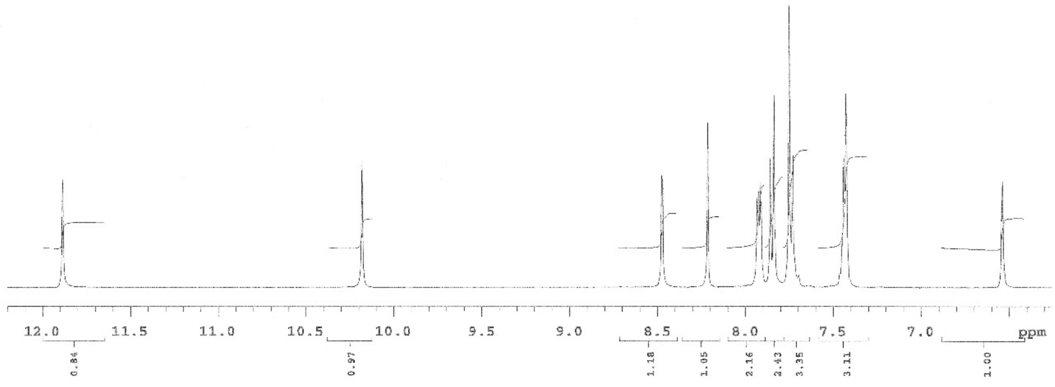

Figure S4: ^{1}H NMR spectrum of compound **2a** (6-12 ppm)

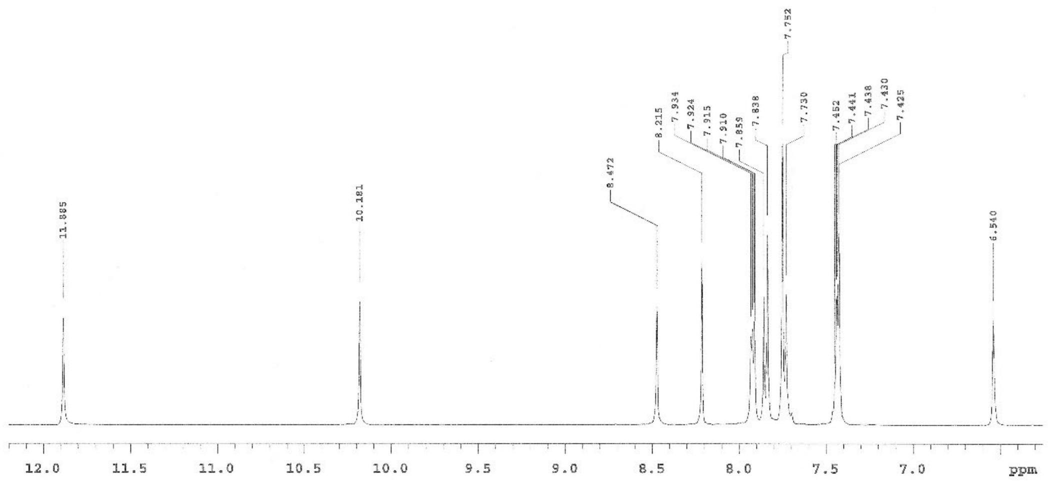

Figure S5: ^{13}C NMR spectrum of compound **2a**

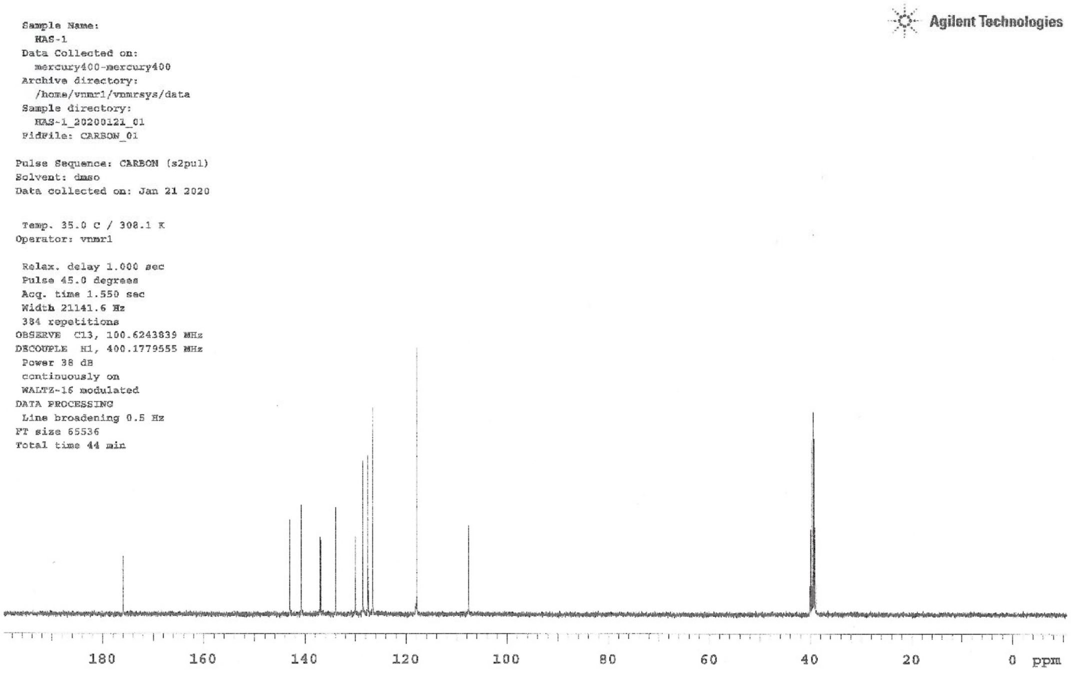

Figure S6: HRMS spectrum of compound **2a**

Formula Predictor Report - has-1_41.lcd

Data File: C:\LabSolutions\Data\Analiz\mdaltintop\has-1_41.lcd

Elmt	Val.	Min	Max	Elmt	Val.	Min	Max	Elmt	Val.	Min	Max	Elmt	Val.	Min	Max	Use Adduct
H	1	6	40	O	2	0	5	S	2	0	4	Ru	2	0	0	H
C	4	7	33	F	1	0	0	Cl	1	0	0	Pd	2	0	0	
N	3	3	7	P	3	0	0	Br	1	0	0	I	3	0	0	

Error Margin (ppm):	5	DBE Range:	5.0 - 20.0	Electron Ions:	both
HC Ratio:	unlimited	Apply N Rule:	yes	Use MSn Info:	yes
Max Isotopes:	3	Isotope RI (%):	1.00	Isotope Res:	9000
MSn Iso RI (%):	10.00	MSn Logic Mode:	AND	Max Results:	100

Event#: 1 MS(E+) Ret. Time : 4.120 Scan# : 619

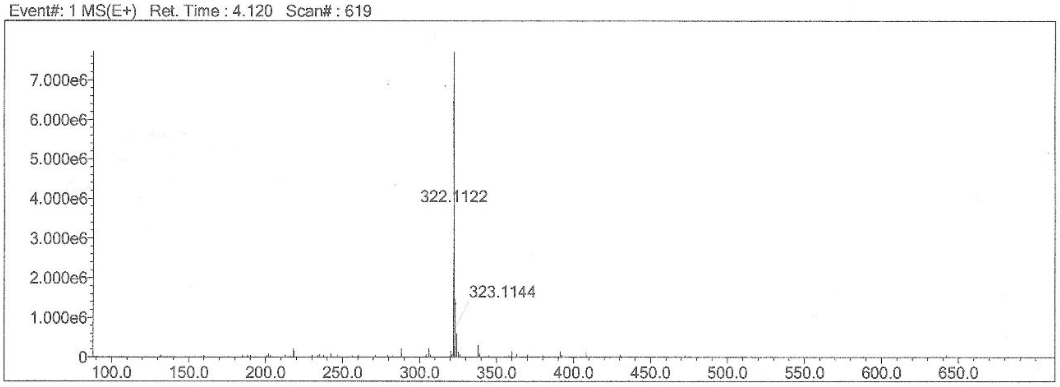

Measured region for 322.1122 m/z

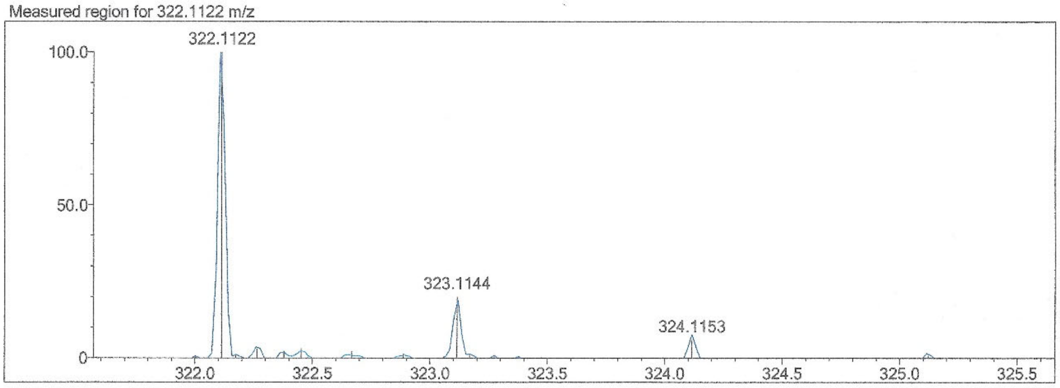

C17 H15 N5 S [M+H]+ : Predicted region for 322.1121 m/z

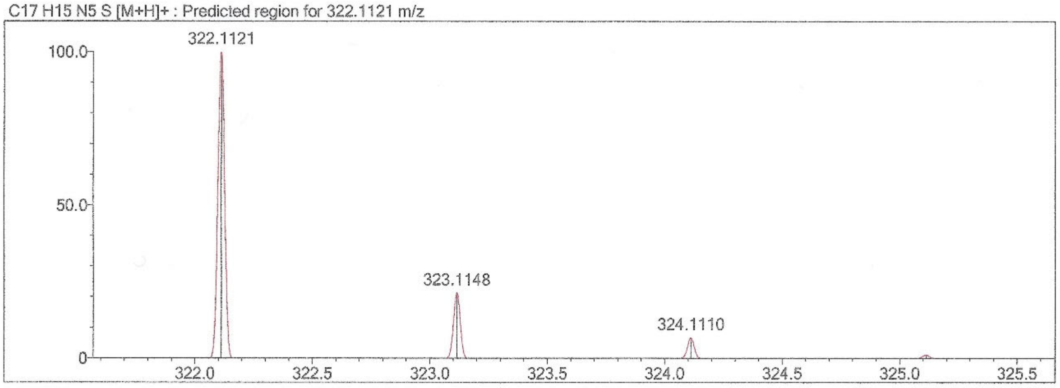

Rank	Score	Formula (M)	Ion	Meas. m/z	Pred. m/z	Df. (mDa)	Df. (ppm)	Iso	DBE
1	60.07	C17 H15 N5 S	[M+H]+	322.1122	322.1121	0.1	0.31	60.07	13.0

Figure S7: IR spectrum of compound **2b**

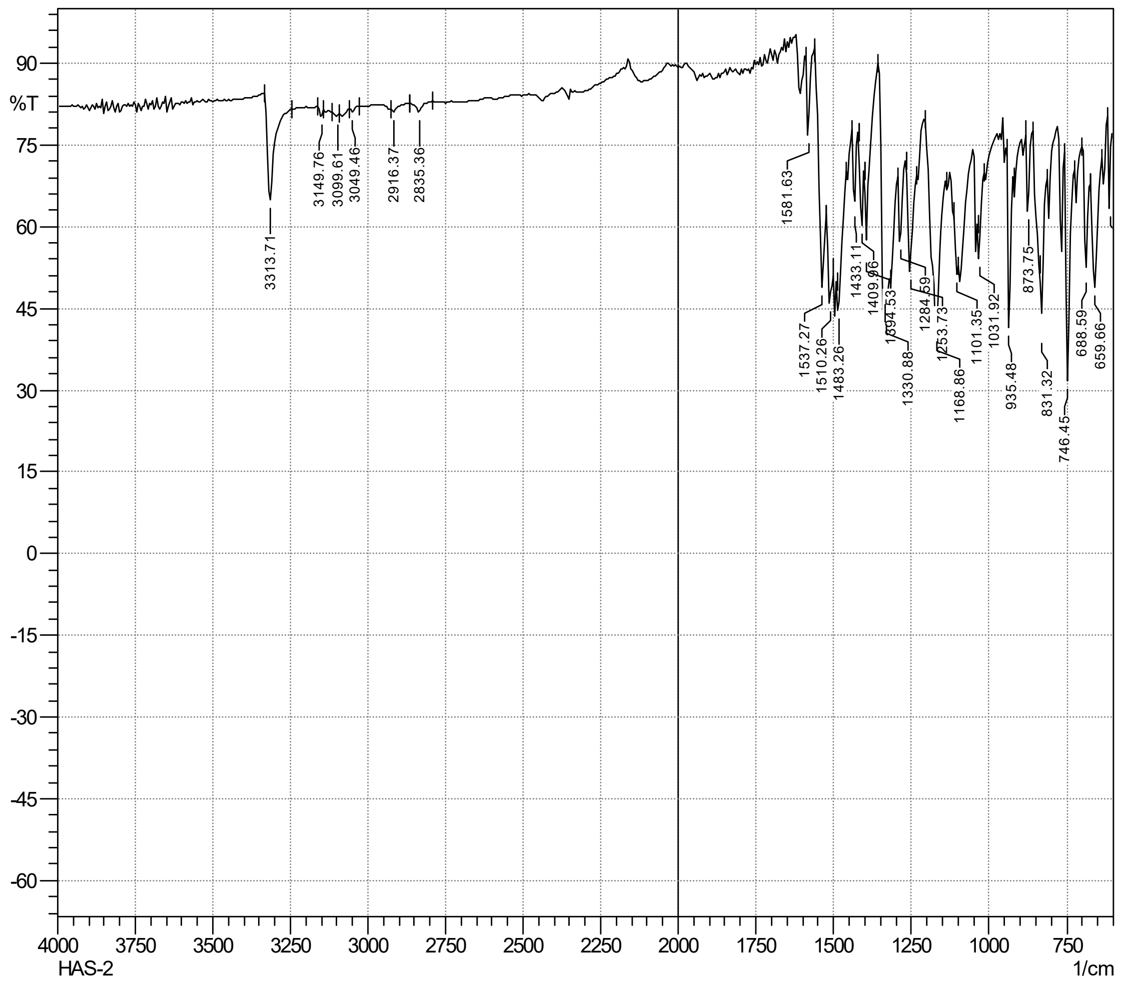

Figure S8: ^{1}H NMR spectrum of compound **2b**

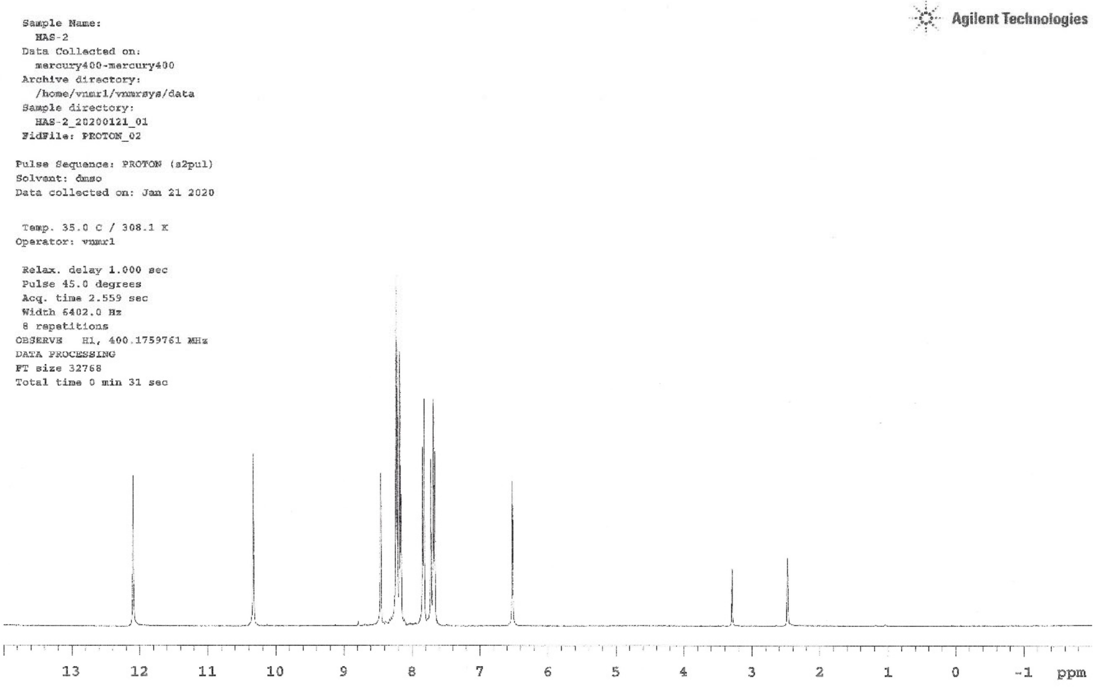

Figure S9: ^{1}H NMR spectrum of compound **2b** with integral values

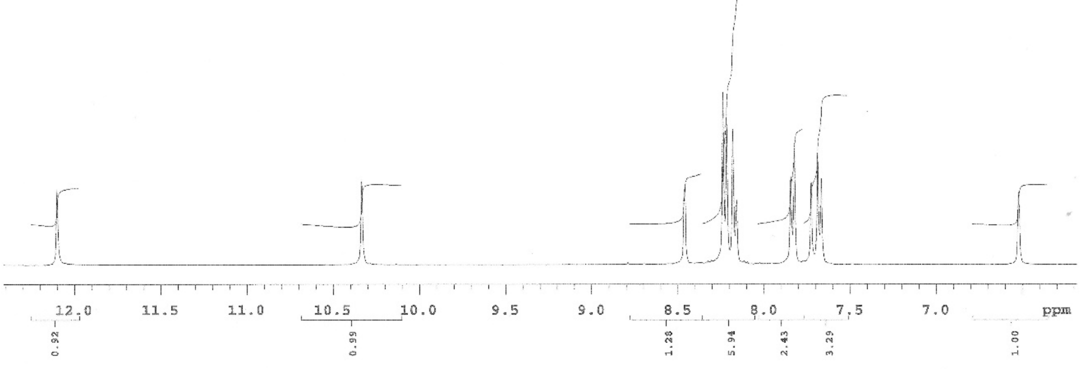

Figure S10: ^{1}H NMR spectrum of compound **2b** (6-12 ppm)

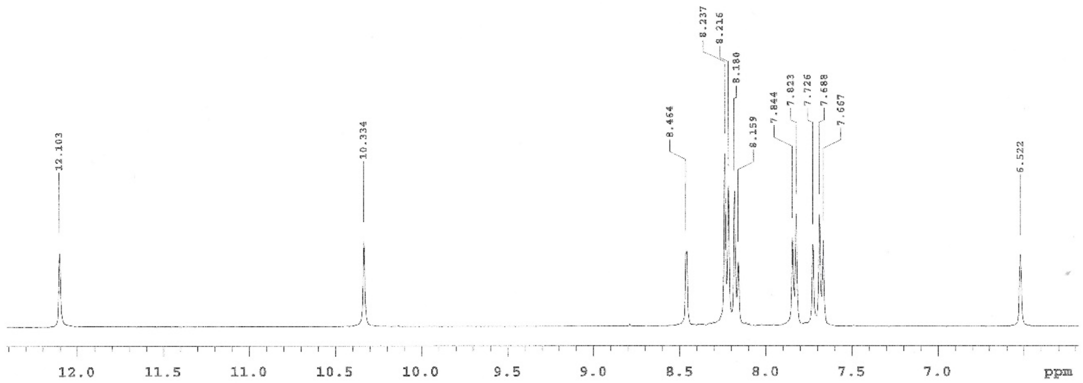

Figure S11: ^{13}C NMR spectrum of compound **2b**

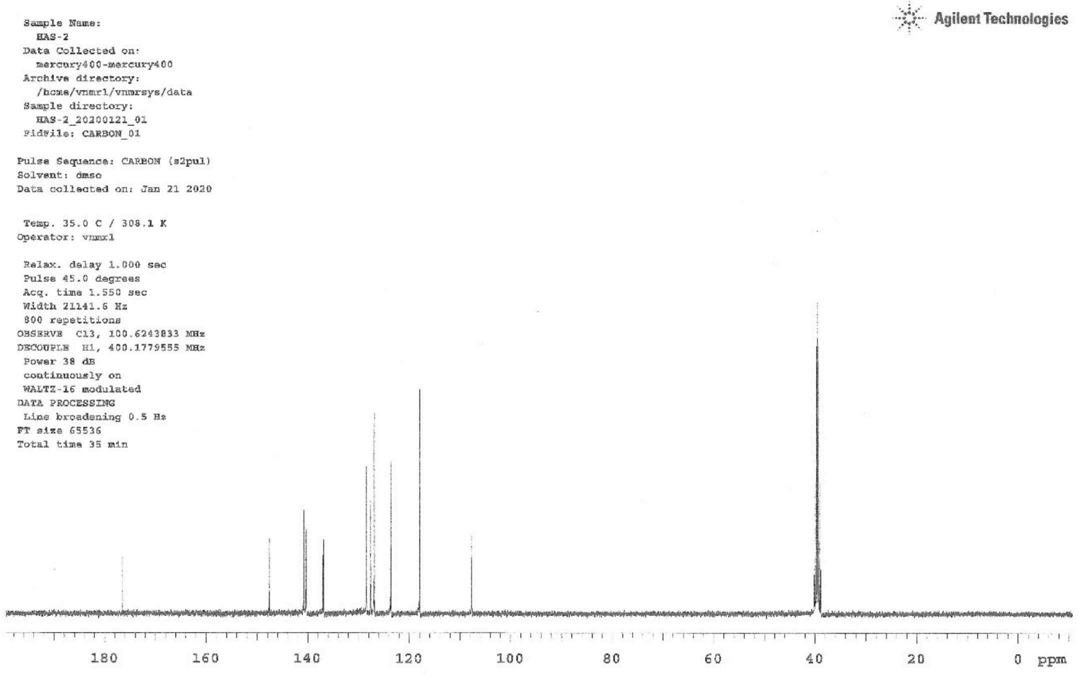

Figure S12: HRMS spectrum of compound **2b**

Data File: C:\LabSolutions\Data\Analiz\mdaltintop\has-2_42.lcd

Elmt	Val.	Min	Max	Elmt	Val.	Min	Max	Elmt	Val.	Min	Max	Elmt	Val.	Min	Max	Use Adduct
H	1	6	40	O	2	0	5	S	2	0	4	Ru	2	0	0	H
C	4	7	33	F	1	0	0	Cl	1	0	0	Pd	2	0	0	
N	3	3	7	P	3	0	0	Br	1	0	0	I	3	0	0	

Error Margin (ppm):	5	DBE Range:	5.0 - 20.0	Electron Ions:	both
HC Ratio:	unlimited	Apply N Rule:	yes	Use MSn Info:	yes
Max Isotopes:	3	Isotope RI (%):	1.00	Isotope Res:	9000
MSn Iso RI (%):	10.00	MSn Logic Mode:	AND	Max Results:	100

Event#: 1 MS(E+) Ret. Time : 4.227 Scan# : 635

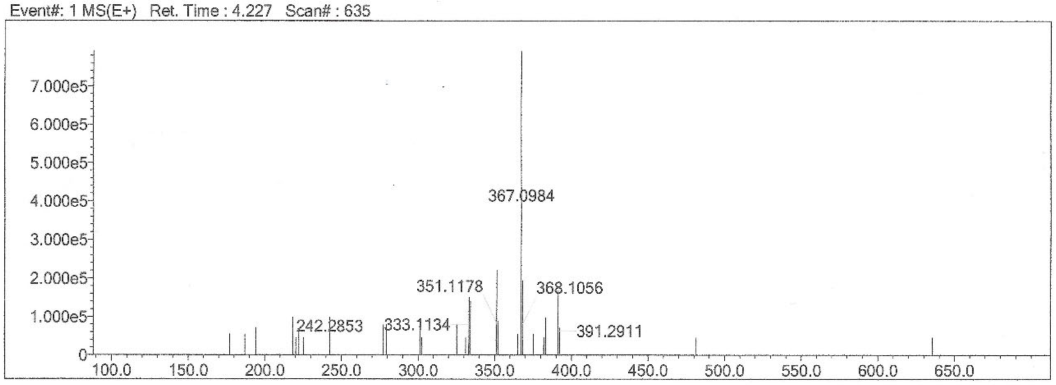

Measured region for 367.0984 m/z

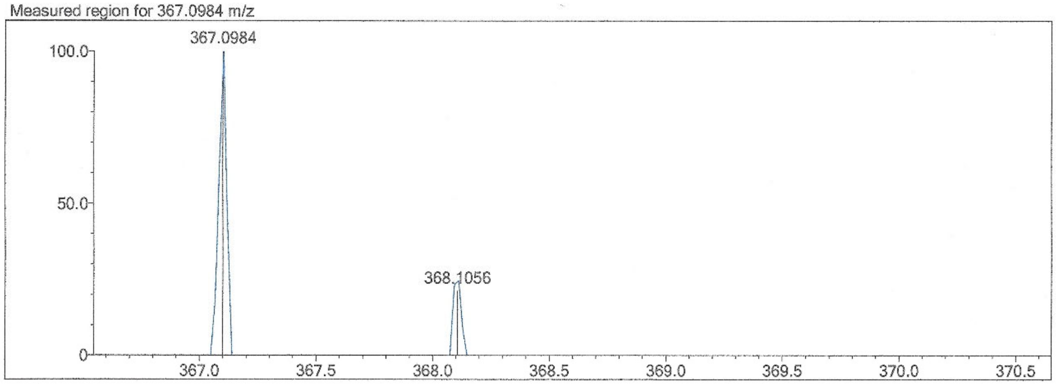

C17 H14 N6 O2 S [M+H]+ : Predicted region for 367.0972 m/z

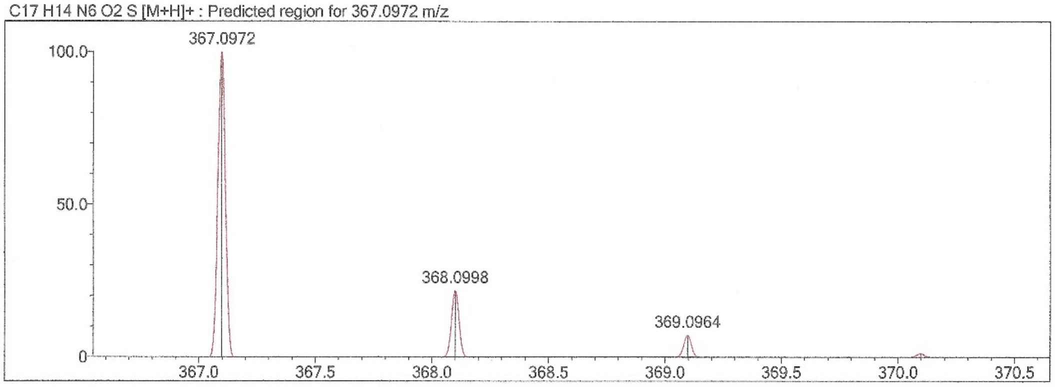

Rank	Score	Formula (M)	Ion	Meas. m/z	Pred. m/z	Df. (mDa)	Df. (ppm)	Iso	DBE
1	0.00	C17 H14 N6 O2 S	[M+H]+	367.0984	367.0972	1.2	3.27	0.00	14.0

Figure S13: IR spectrum of compound **2c**

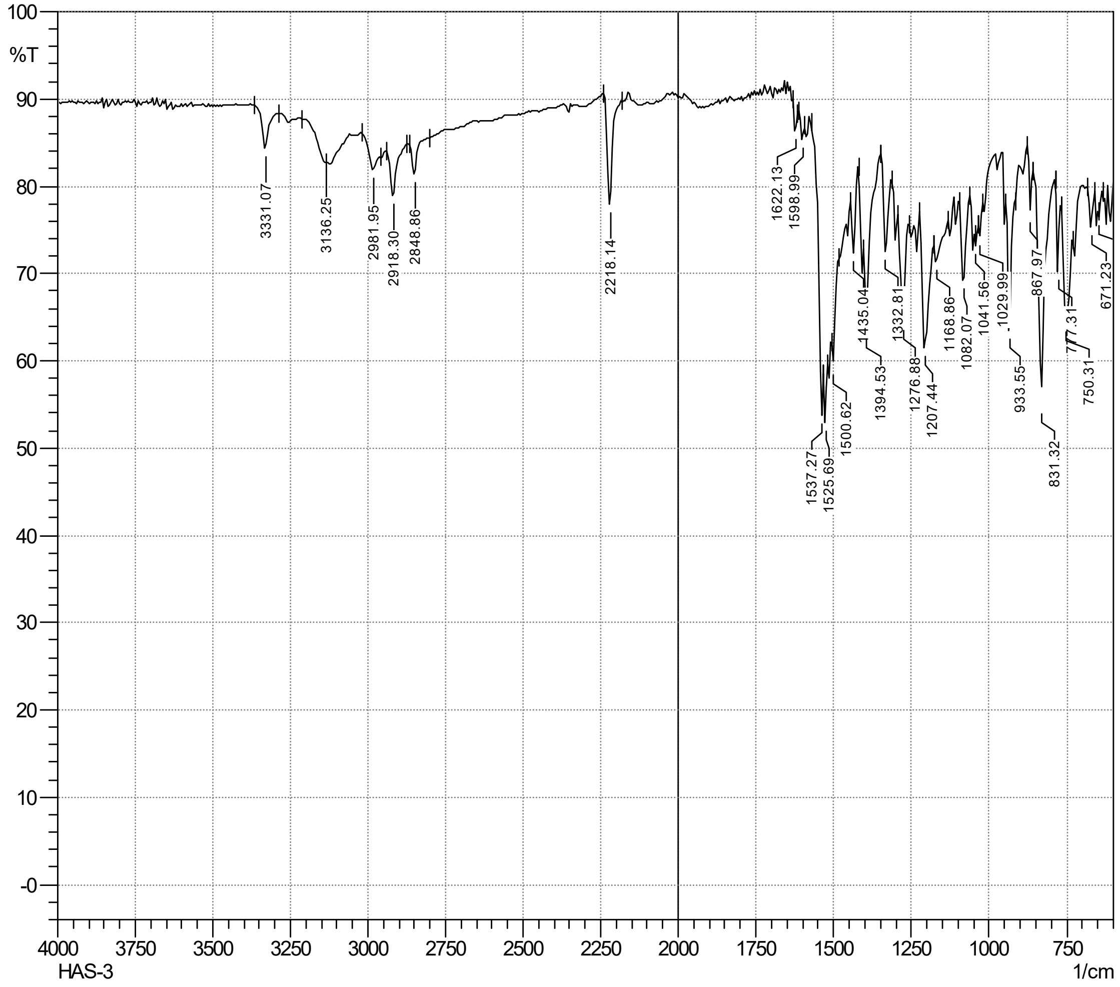

Figure S14: ^{1}H NMR spectrum of compound **2c**

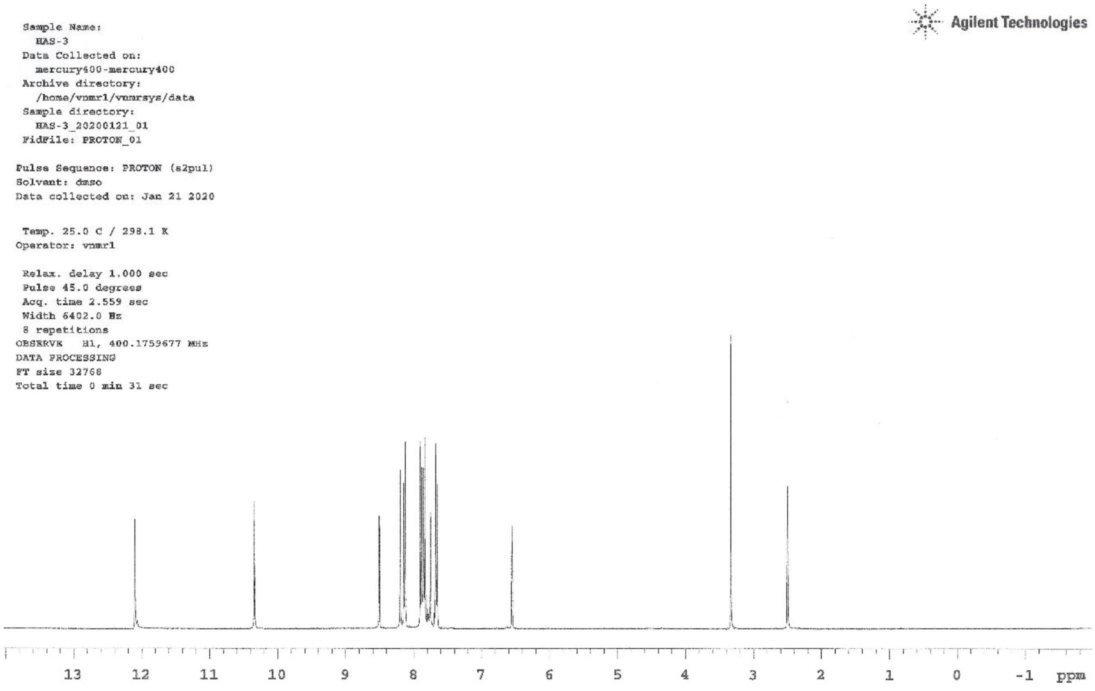

Figure S15: ^{1}H NMR spectrum of compound **2c** with integral values

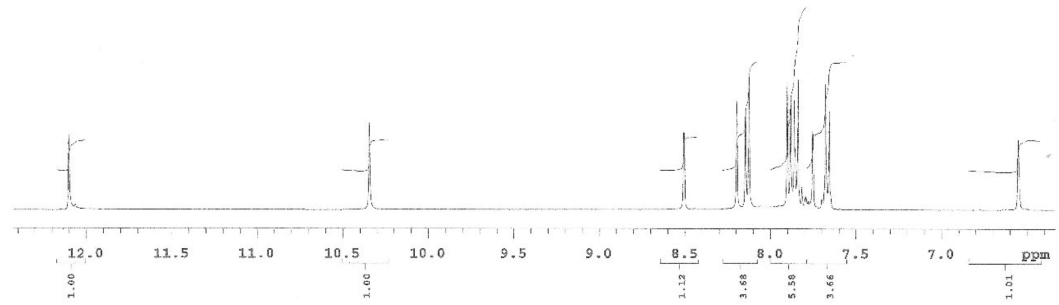

Figure S16: ^{1}H NMR spectrum of compound **2c** (6-12 ppm)

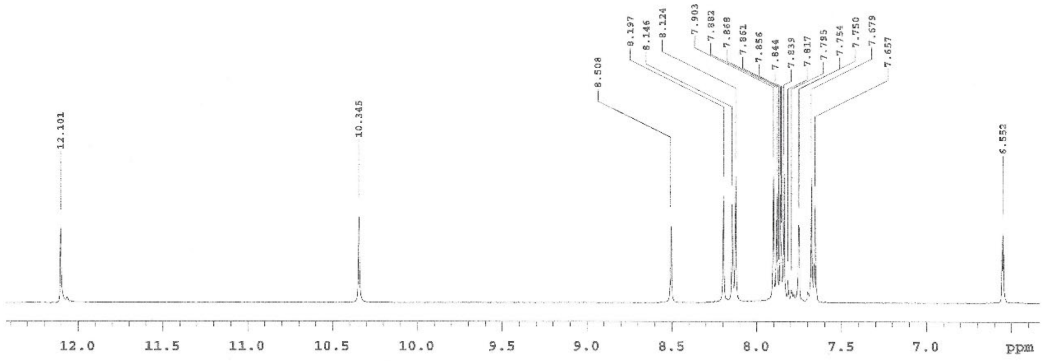

Figure S17: ^{13}C NMR spectrum of compound **2c**

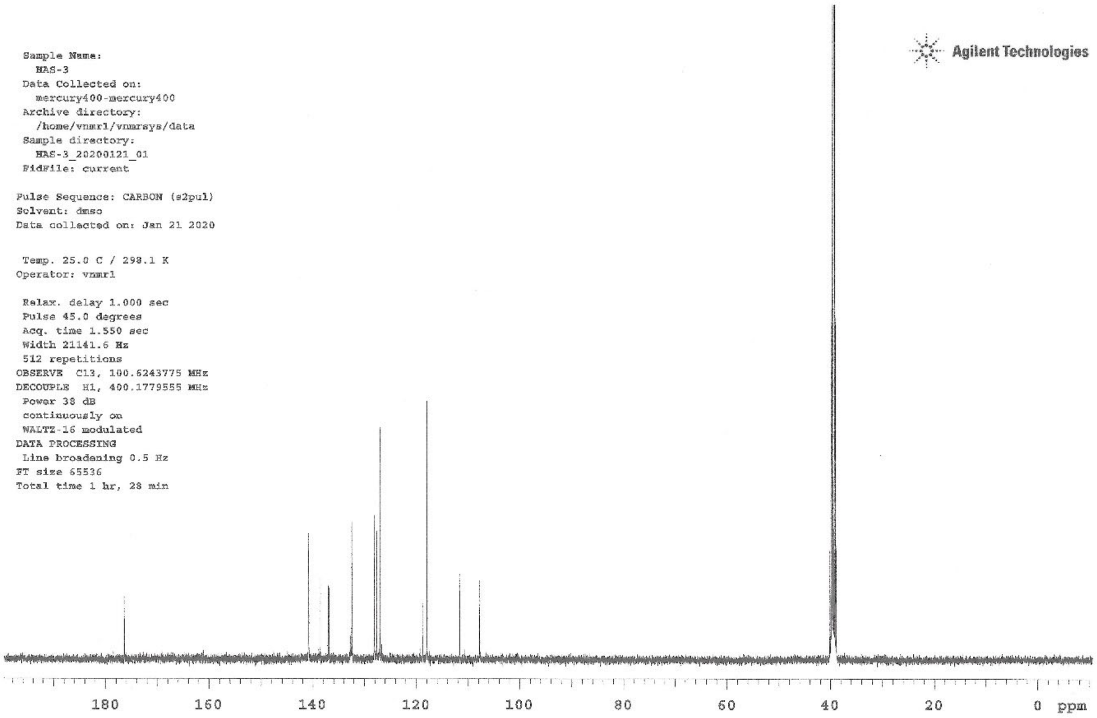

Figure S18: HRMS spectrum of compound **2c**

Formula Predictor Report - has-3_43.lcd

Page 1 of 1

Data File: C:\LabSolutions\Data\Analiz\mdaltintop\has-3_43.lcd

Elmt	Val.	Min	Max	Elmt	Val.	Min	Max	Elmt	Val.	Min	Max	Elmt	Val.	Min	Max	Use Adduct
H	1	6	40	O	2	0	5	S	2	0	4	Ru	2	0	0	H
C	4	7	33	F	1	0	0	Cl	1	0	0	Pd	2	0	0	
N	3	3	7	P	3	0	0	Br	1	0	0	I	3	0	0	

Error Margin (ppm):	5	DBE Range:	5.0 - 20.0	Electron Ions:	both
HC Ratio:	unlimited	Apply N Rule:	yes	Use MSn Info:	yes
Max Isotopes:	3	Isotope RI (%):	1.00	Isotope Res:	9000
MSn Iso RI (%):	10.00	MSn Logic Mode:	AND	Max Results:	100

Event#: 1 MS(E+) Ret. Time : 3.813 Scan# : 573

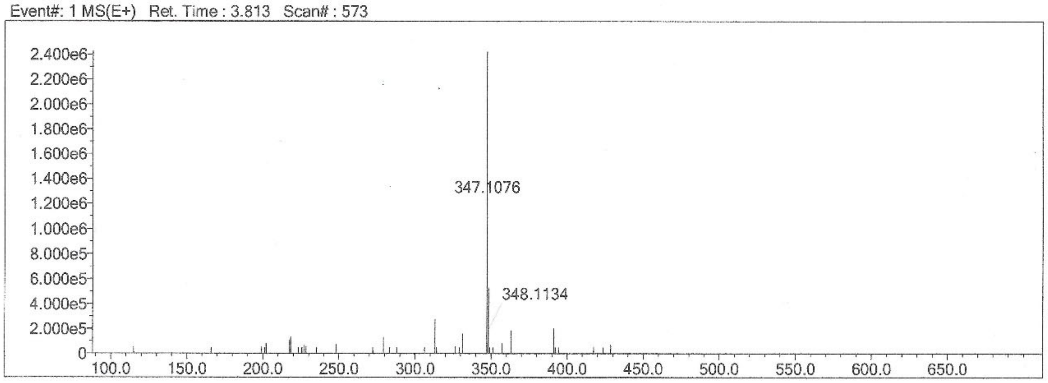

Measured region for 347.1076 m/z

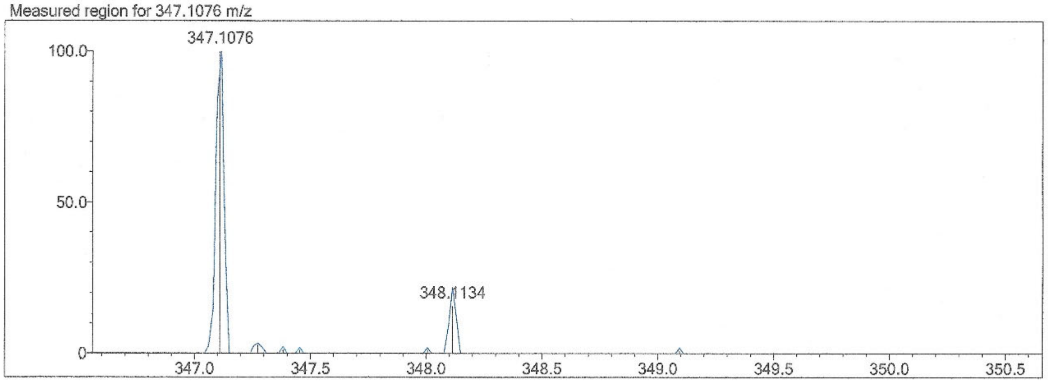

C18 H14 N6 S [M+H]+ : Predicted region for 347.1073 m/z

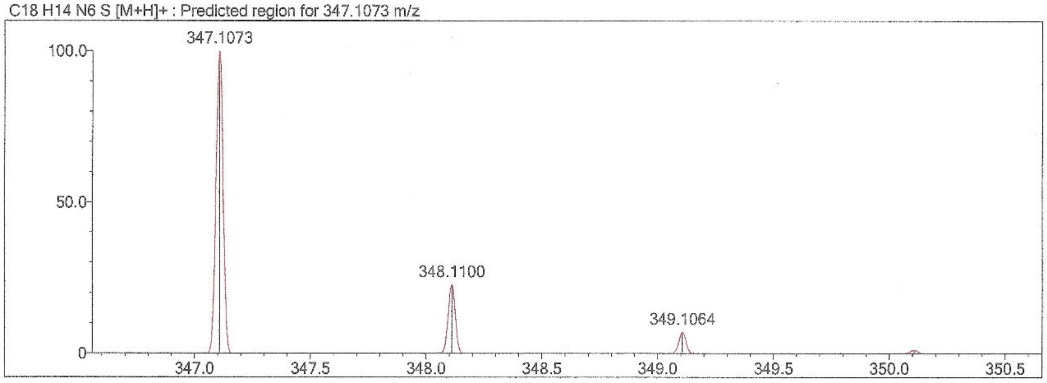

Rank	Score	Formula (M)	Ion	Meas. m/z	Pred. m/z	Df. (mDa)	Df. (ppm)	Iso	DBE
1	41.29	C18 H14 N6 S	[M+H]+	347.1076	347.1073	0.3	0.86	41.29	15.0

Figure S19: IR spectrum of compound **2d**

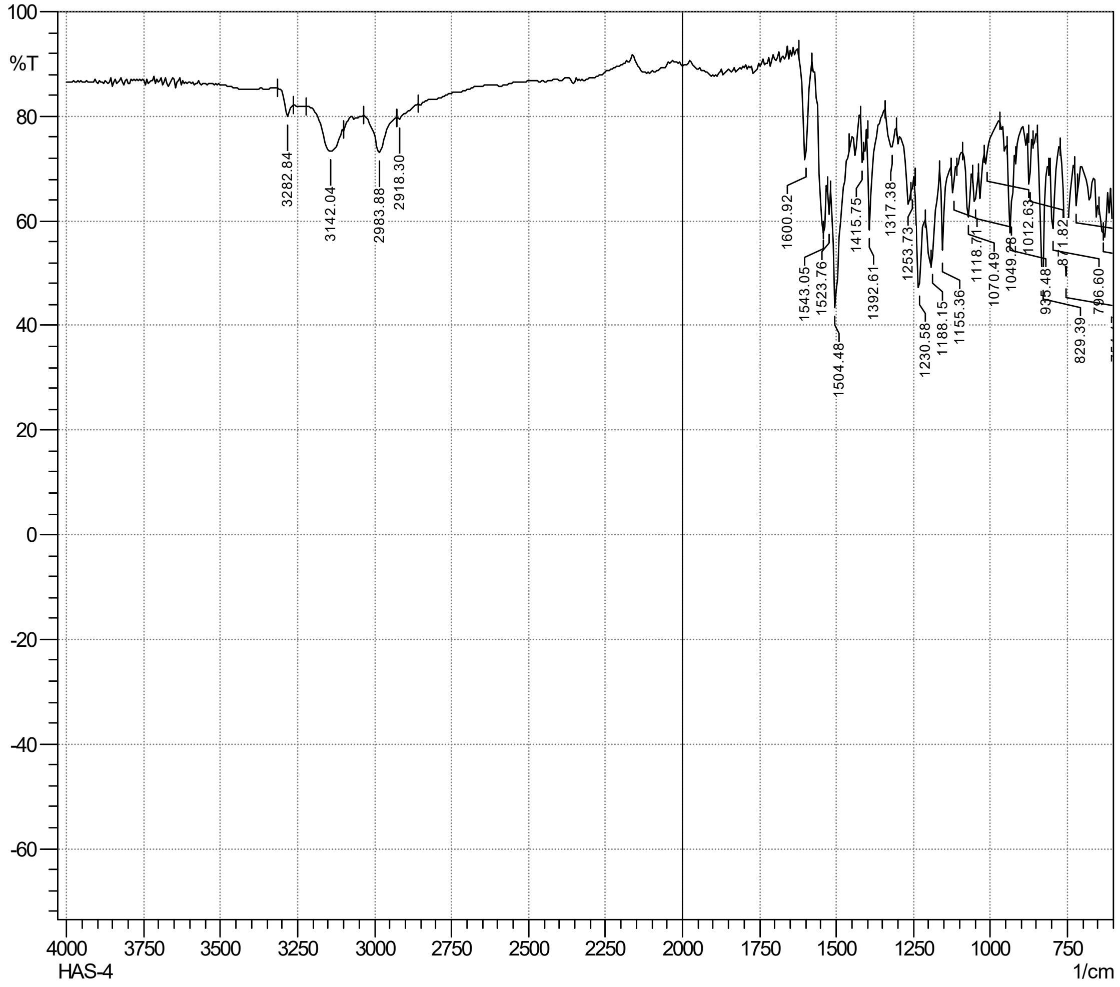

Figure S20: ^{1}H NMR spectrum of compound **2d**

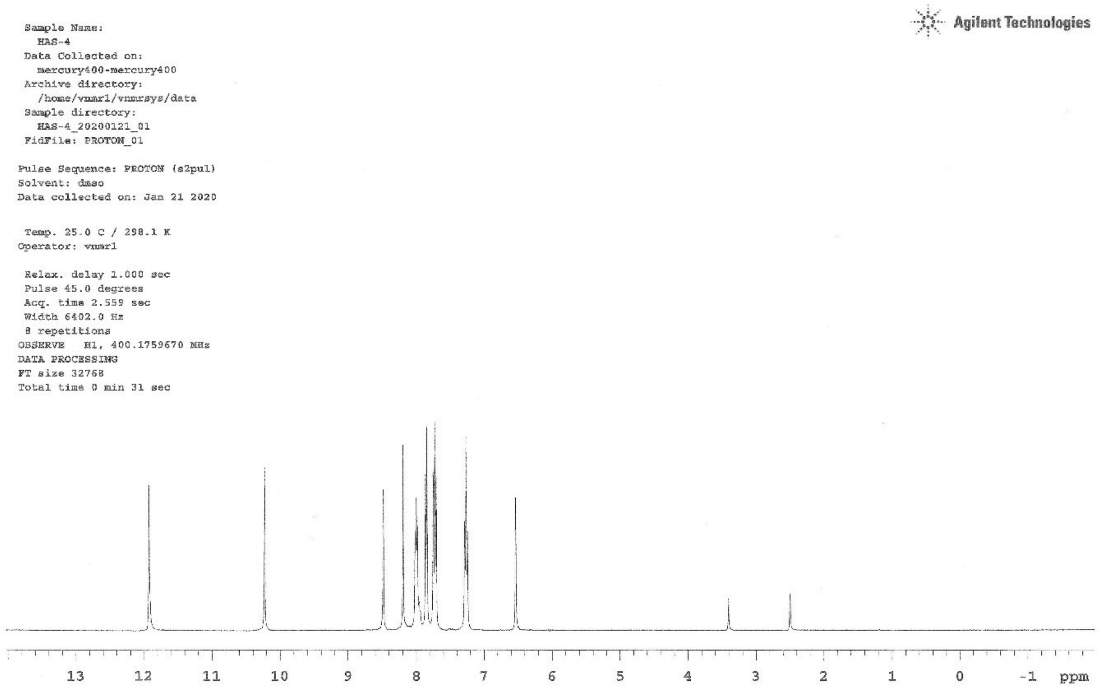

Figure S21: ^{1}H NMR spectrum of compound **2d** with integral values

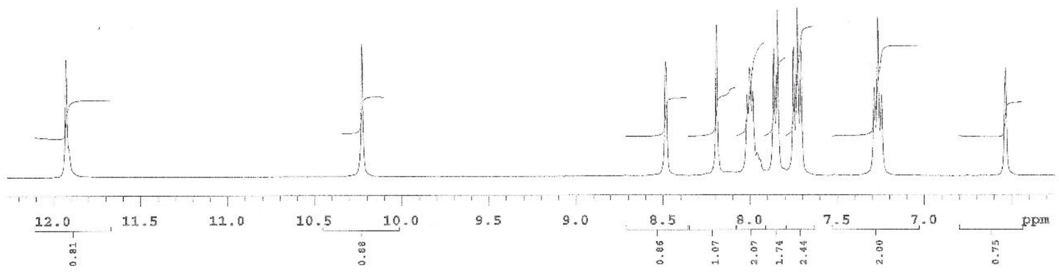

Figure S22: ^{1}H NMR spectrum of compound **2d** (6-12 ppm)

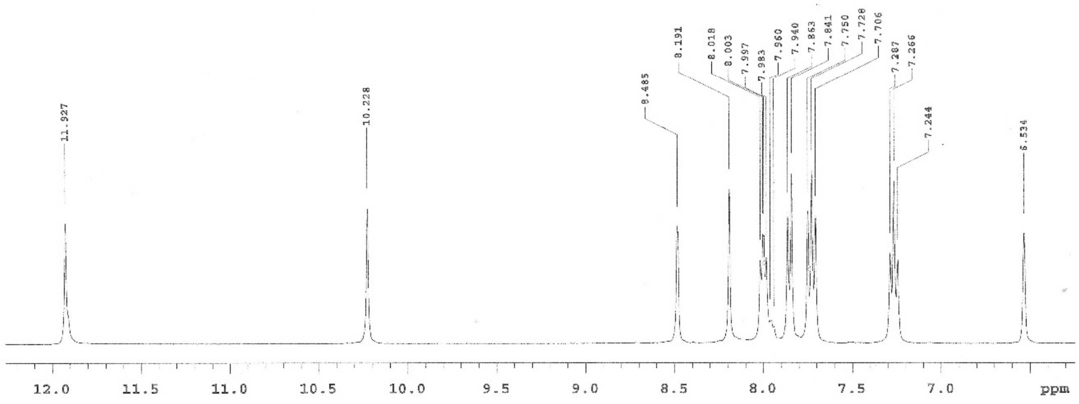

Figure S23: ^{13}C NMR spectrum of compound **2d**

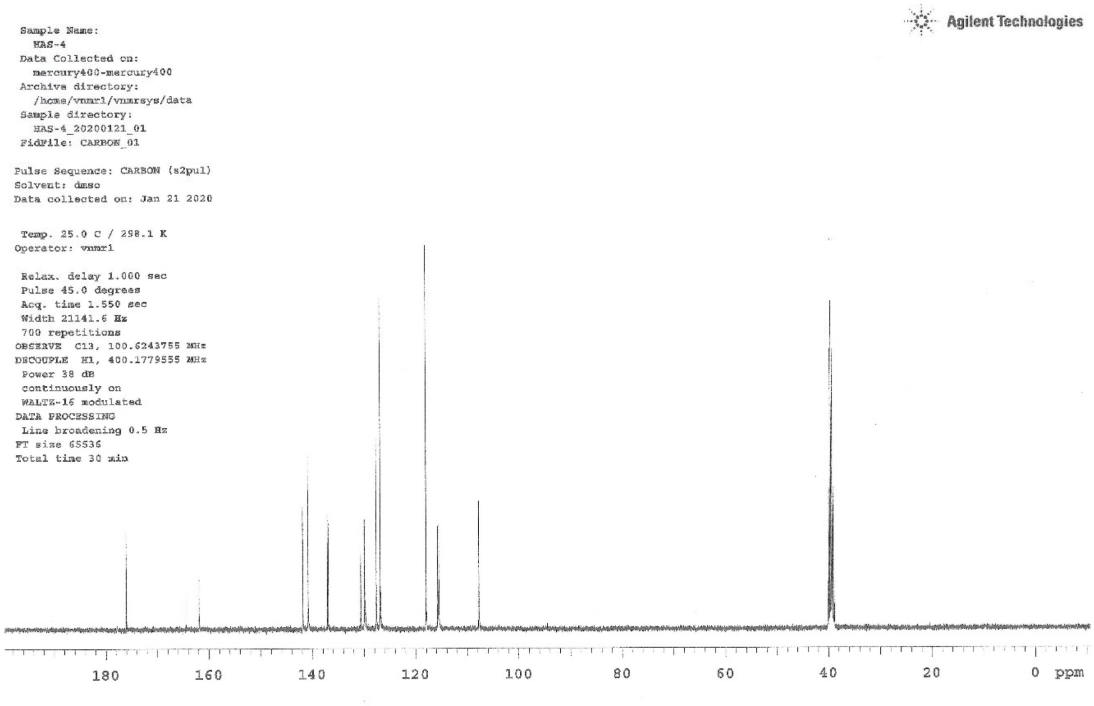

Figure S24: HRMS spectrum of compound **2d**

Formula Predictor Report - has-4_44.lcd

Data File: C:\LabSolutions\Data\Analiz\mdaltintop\has-4_44.lcd

Elmt	Val.	Min	Max	Elmt	Val.	Min	Max	Elmt	Val.	Min	Max	Elmt	Val.	Min	Max	Use Adduct
H	1	6	40	O	2	0	5	S	2	0	4	Ru	2	0	0	H
C	4	7	33	F	1	1	1	Cl	1	0	0	Pd	2	0	0	
N	3	3	7	P	3	0	0	Br	1	0	0	I	3	0	0	

Error Margin (ppm): 5
HC Ratio: unlimited
Max Isotopes: 3
MSn Iso RI (%): 10.00

DBE Range: 5.0 - 20.0
Apply N Rule: yes
Isotope RI (%): 1.00
MSn Logic Mode: AND

Electron Ions: both
Use MSn Info: yes
Isotope Res: 9000
Max Results: 100

Event#: 1 MS(E+) Ret. Time : 4.040 -> 4.427 Scan# : 607 -> 665

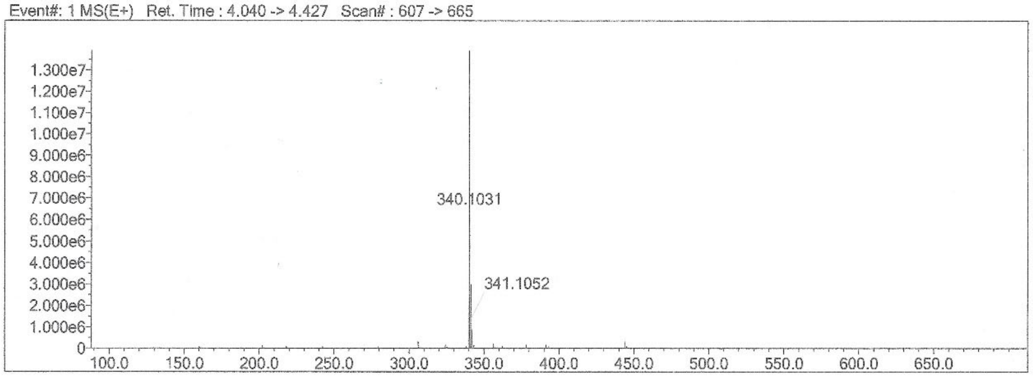

Measured region for 340.1031 m/z

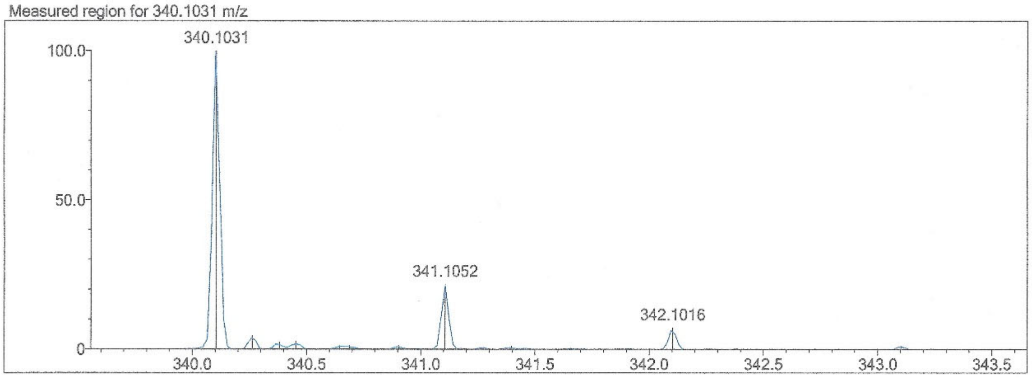

C17 H14 N5 F S [M+H]+ : Predicted region for 340.1027 m/z

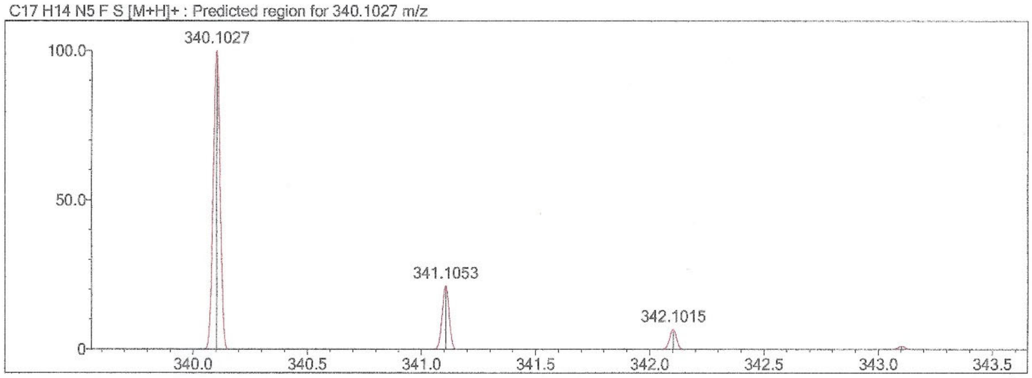

Rank	Score	Formula (M)	Ion	Meas. m/z	Pred. m/z	Df. (mDa)	Df. (ppm)	Iso	DBE
1	92.27	C17 H14 N5 F S	[M+H]+	340.1031	340.1027	0.4	1.18	92.68	13.0

Figure S25: IR spectrum of compound **2e**

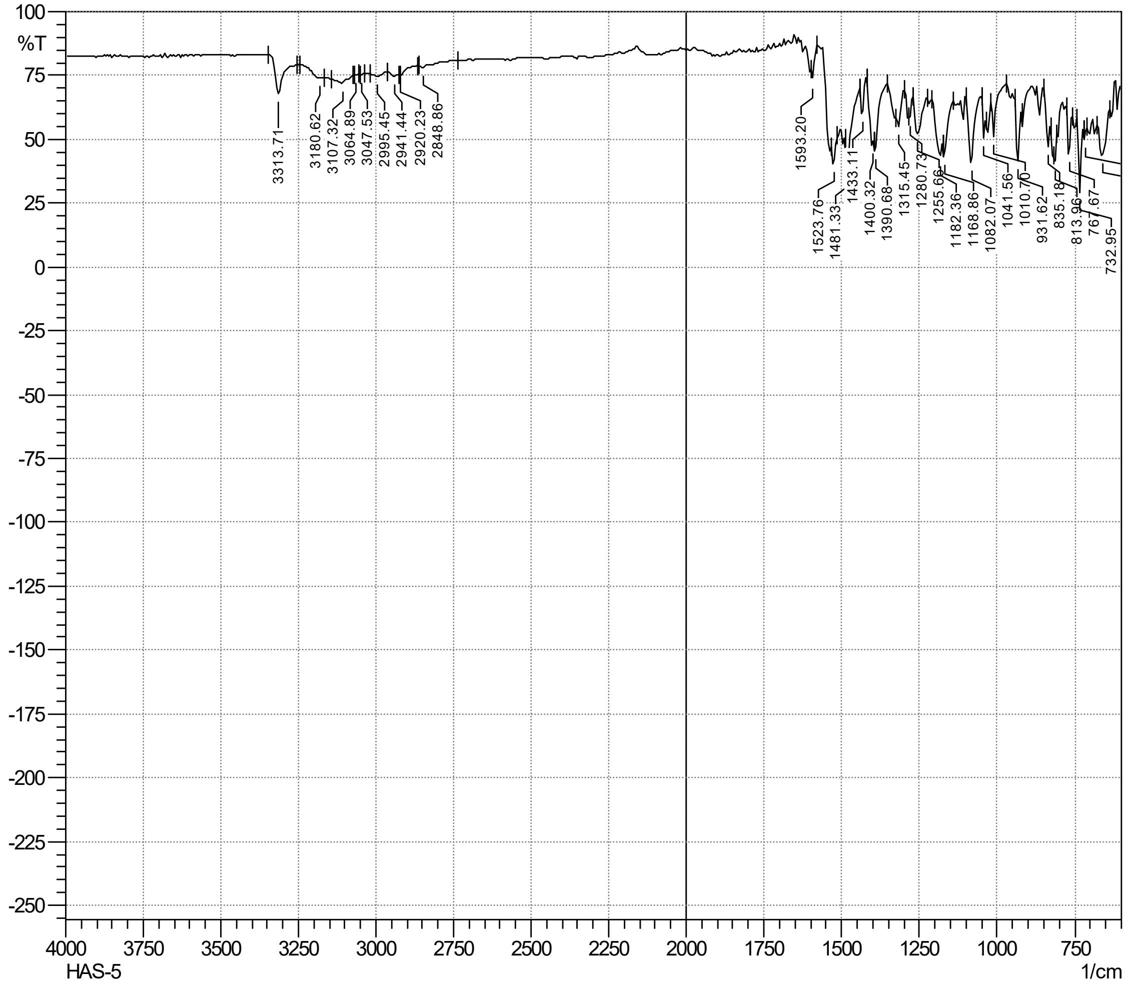

Figure S26: ^{1}H NMR spectrum of compound **2e**

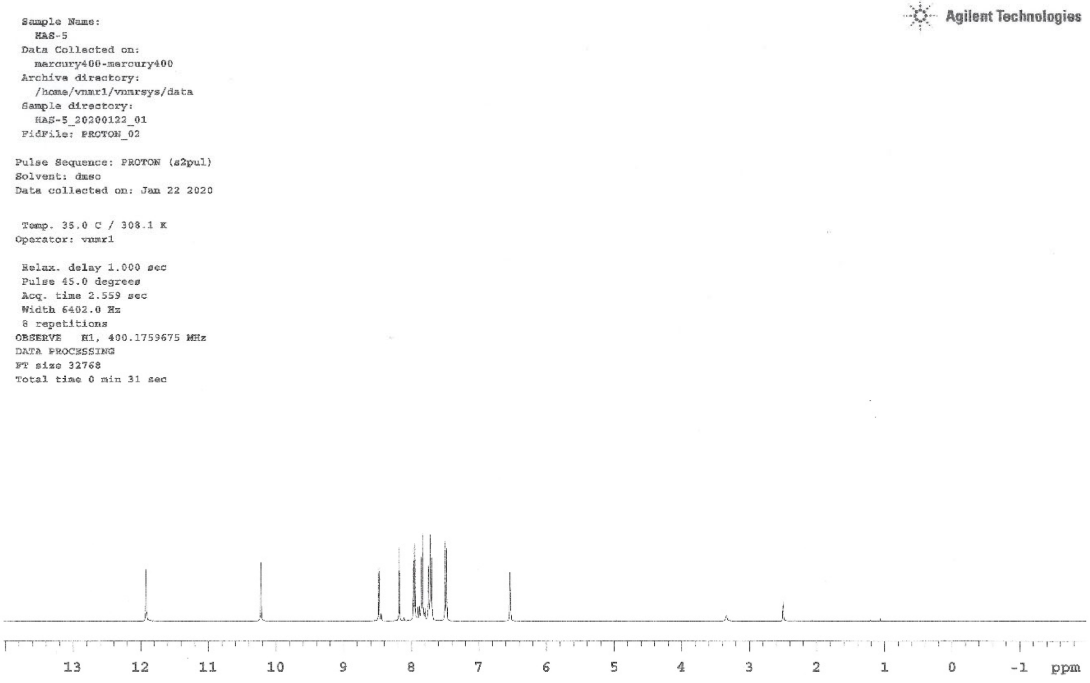

Figure S27: ^{1}H NMR spectrum of compound **2e** with integral values

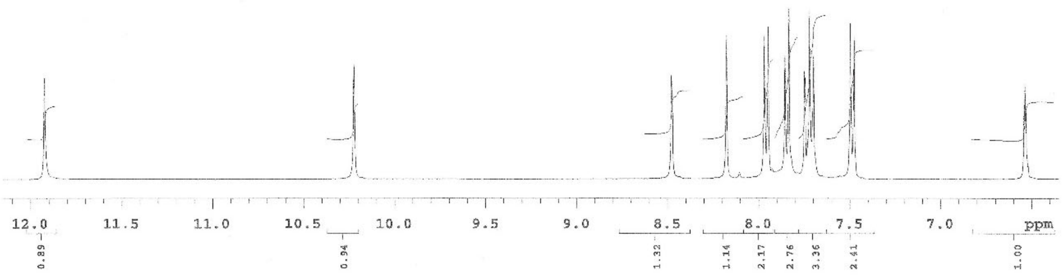

Figure S28: ^{1}H NMR spectrum of compound **2e** (6-12 ppm)

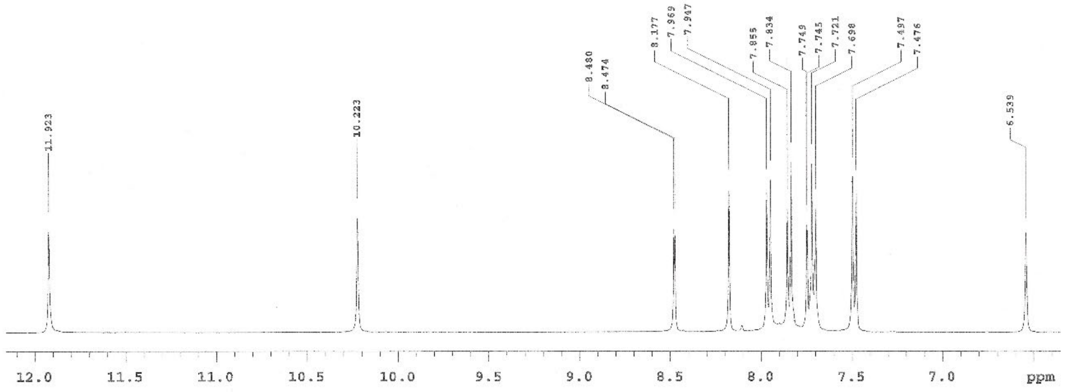

Figure S29: ^{13}C NMR spectrum of compound **2e**

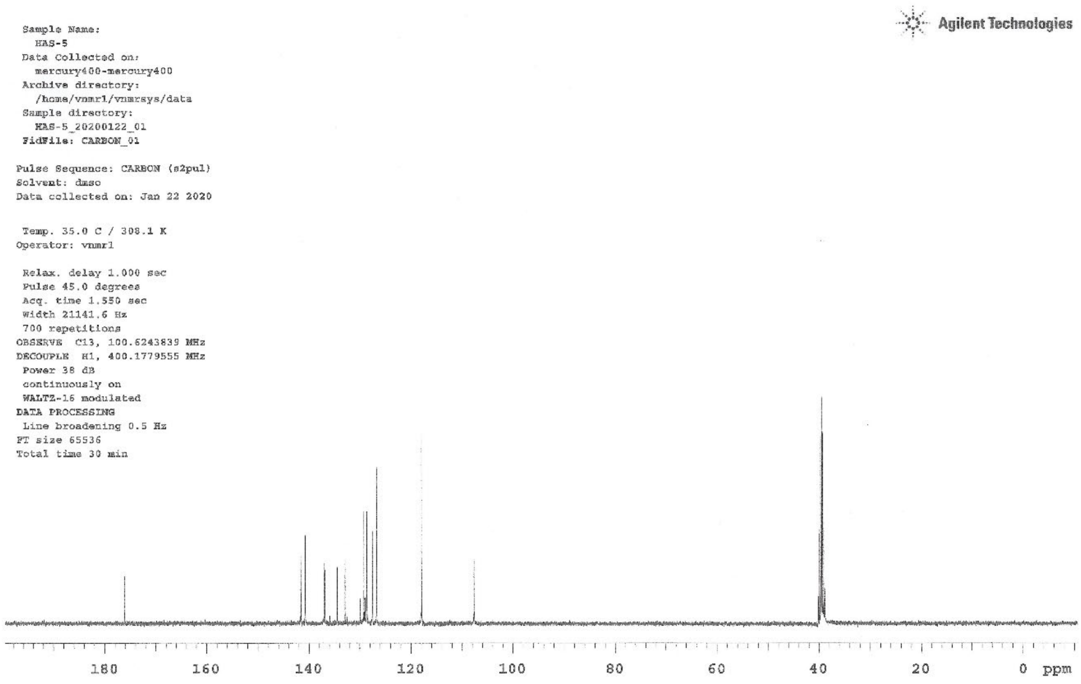

Figure S30: HRMS spectrum of compound **2e**

Formula Predictor Report - has-5_45.lcd

Page 1 of 1

Data File: C:\LabSolutions\Data\Analiz\mdaltintop\has-5_45.lcd

Elmt	Val.	Min	Max	Elmt	Val.	Min	Max	Elmt	Val.	Min	Max	Elmt	Val.	Min	Max	Use Adduct
H	1	6	40	O	2	0	0	S	2	0	4	Ru	2	0	0	H
C	4	7	33	F	1	0	0	Cl	1	1	1	Pd	2	0	0	
N	3	3	7	P	3	0	0	Br	1	0	0	I	3	0	0	

Error Margin (ppm): 5
HC Ratio: unlimited
Max Isotopes: 3
MSn Iso RI (%): 10.00

DBE Range: 5.0 - 20.0
Apply N Rule: yes
Isotope RI (%): 1.00
MSn Logic Mode: AND

Electron Ions: both
Use MSn Info: yes
Isotope Res: 9000
Max Results: 100

Event#: 1 MS(E+) Ret. Time : 4.760 Scan# : 715

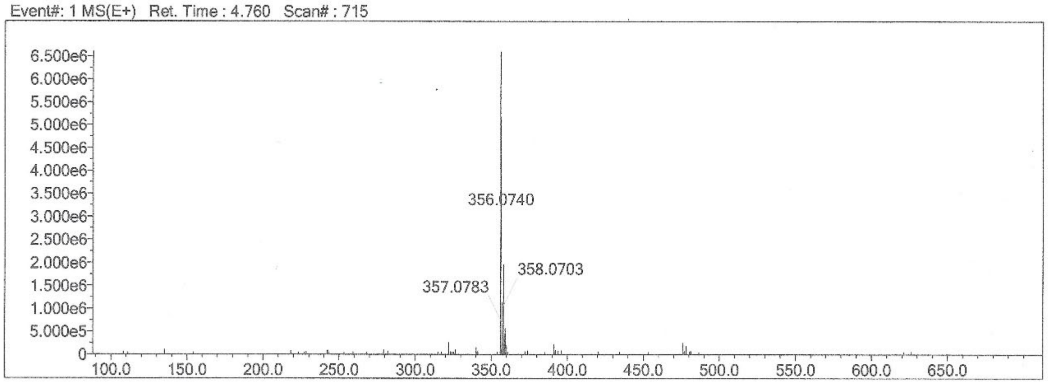

Measured region for 356.0740 m/z

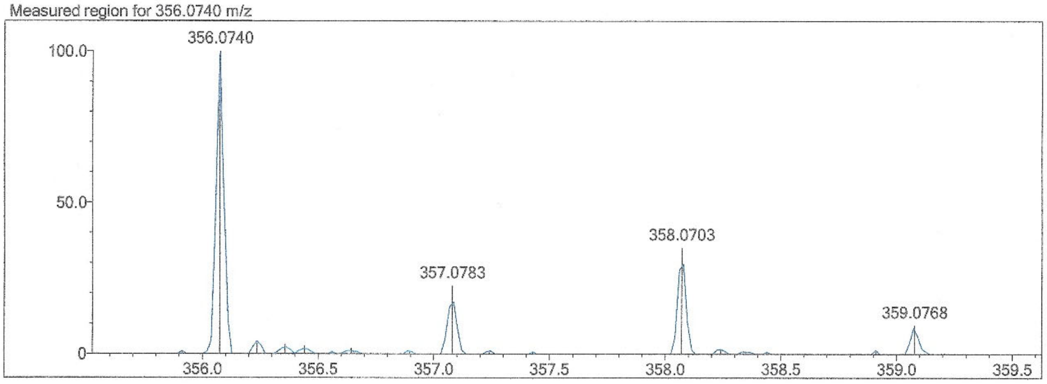

C17 H14 N5 S Cl [M+H]+ : Predicted region for 356.0731 m/z

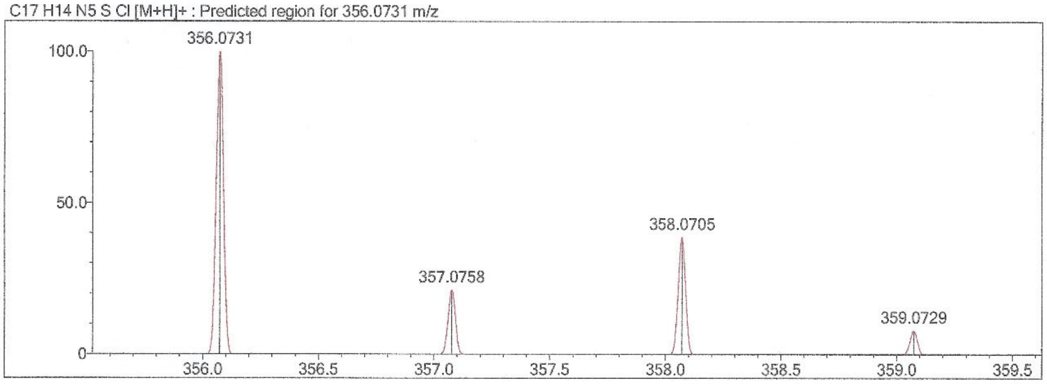

Rank	Score	Formula (M)	Ion	Meas. m/z	Pred. m/z	Df. (mDa)	Df. (ppm)	Iso	DBE
1	76.05	C17 H14 N5 S Cl	[M+H]+	356.0740	356.0731	0.9	2.53	79.07	13.0

Figure S31: IR spectrum of compound **2f**

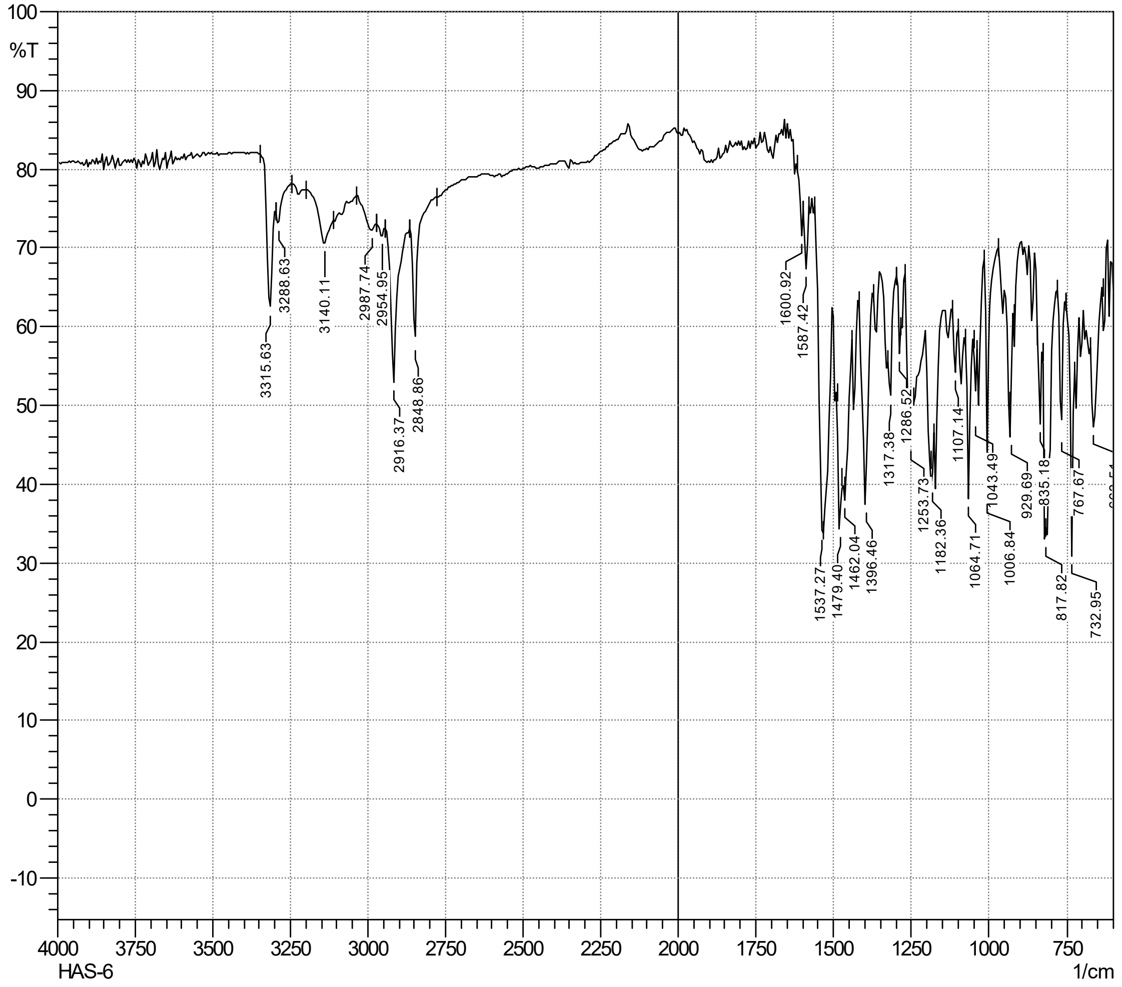

Figure S32: ^{1}H NMR spectrum of compound **2f**

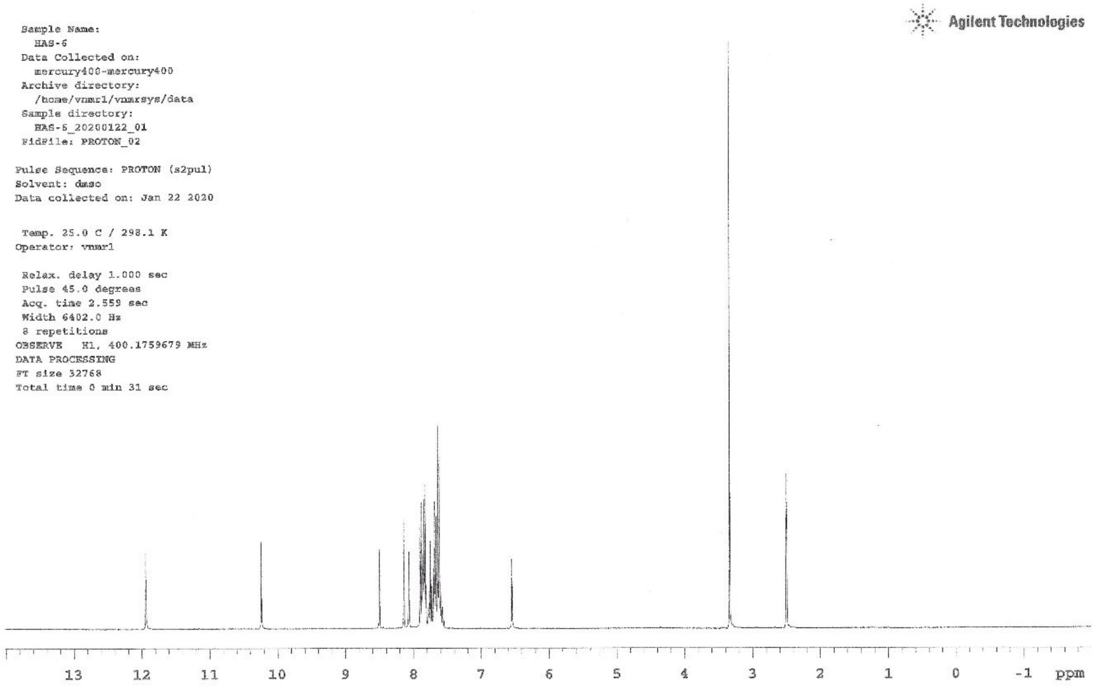

Figure S33: ^{1}H NMR spectrum of compound **2f** with integral values

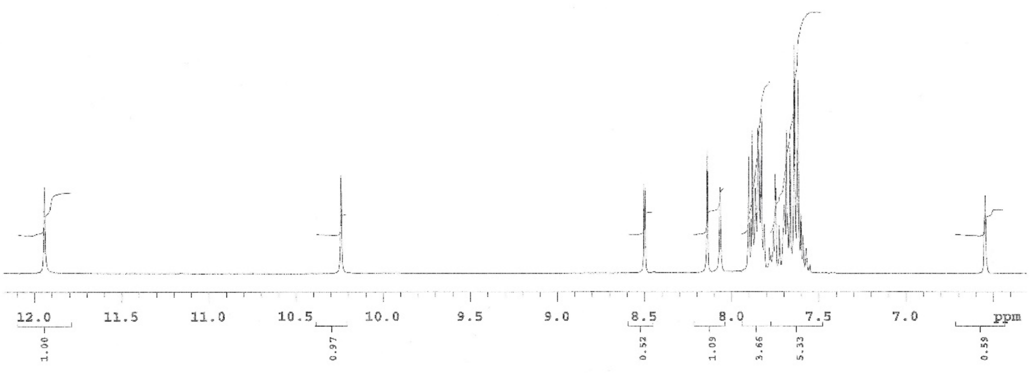

Figure S34: ^{1}H NMR spectrum of compound **2f** (6.6-8.8 ppm)

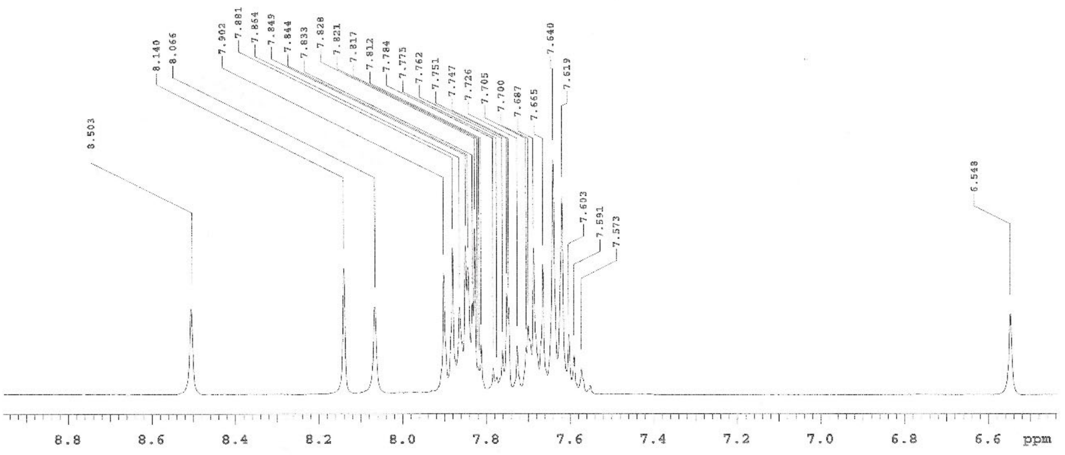

Figure S35: ^{1}H NMR spectrum of compound **2f** (10.2-12.2 ppm)

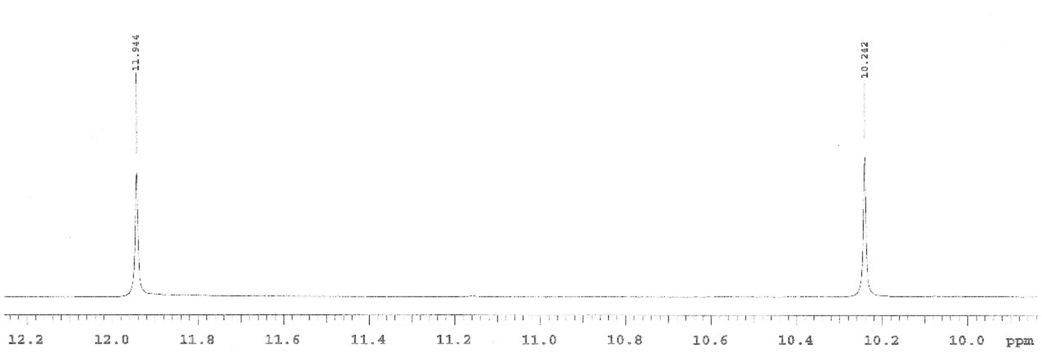

Figure S36: ^{13}C NMR spectrum of compound **2f**

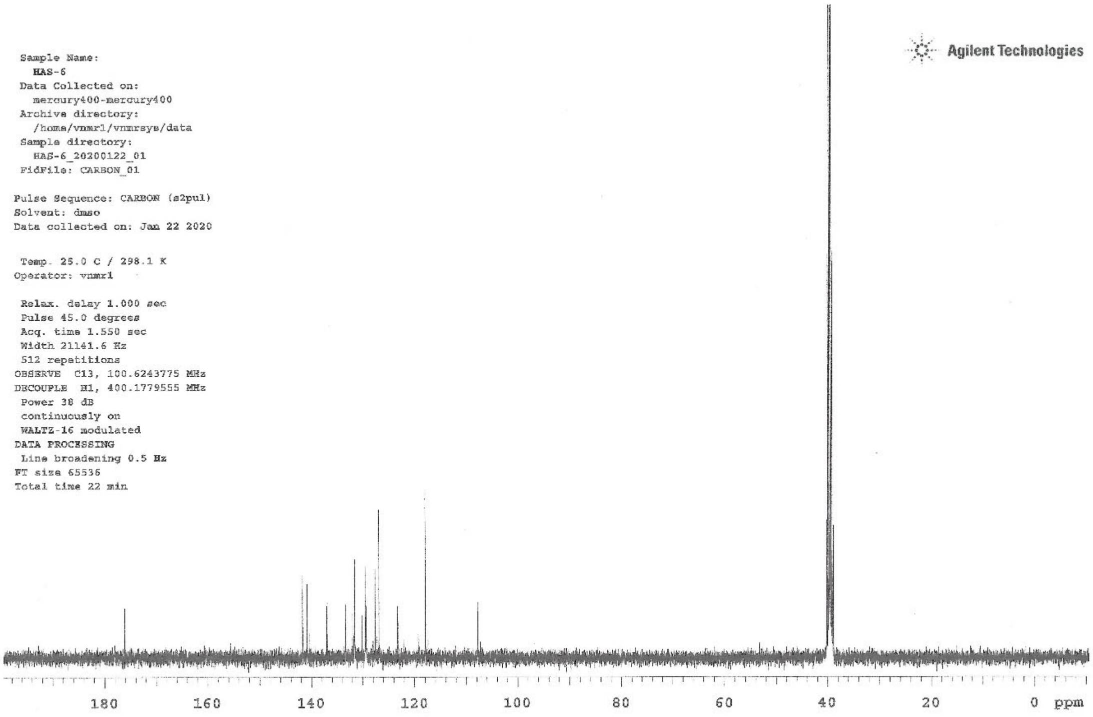

Figure S37: HRMS spectrum of compound **2f**

Formula Predictor Report - has-6_46.lcd　　　　Page 1 of 1

Data File: C:\LabSolutions\Data\Analiz\mdaltintop\has-6_46.lcd

Elmt	Val.	Min	Max	Elmt	Val.	Min	Max	Elmt	Val.	Min	Max	Elmt	Val.	Min	Max	Use Adduct
H	1	6	40	O	2	0	0	S	2	0	4	Ru	2	0	0	H
C	4	7	33	F	1	0	0	Cl	1	0	0	Pd	2	0	0	
N	3	3	7	P	3	0	0	Br	1	1	1	I	3	0	0	

Error Margin (ppm): 5
HC Ratio: unlimited
Max Isotopes: 3
MSn Iso RI (%): 10.00

DBE Range: 5.0 - 20.0
Apply N Rule: yes
Isotope RI (%): 1.00
MSn Logic Mode: AND

Electron Ions: both
Use MSn Info: yes
Isotope Res: 9000
Max Results: 100

Event#: 1 MS(E+) Ret. Time : 4.667 -> 5.067 Scan# : 701 -> 761

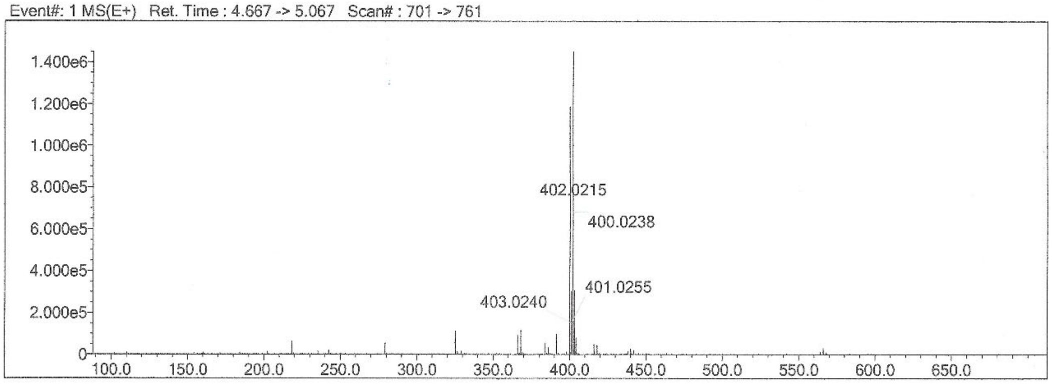

Measured region for 400.0238 m/z

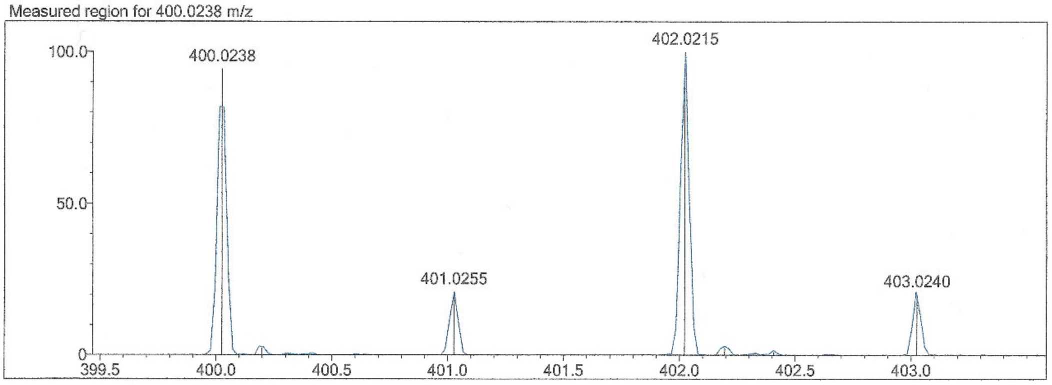

C17 H14 N5 S Br [M+H]+ : Predicted region for 400.0226 m/z

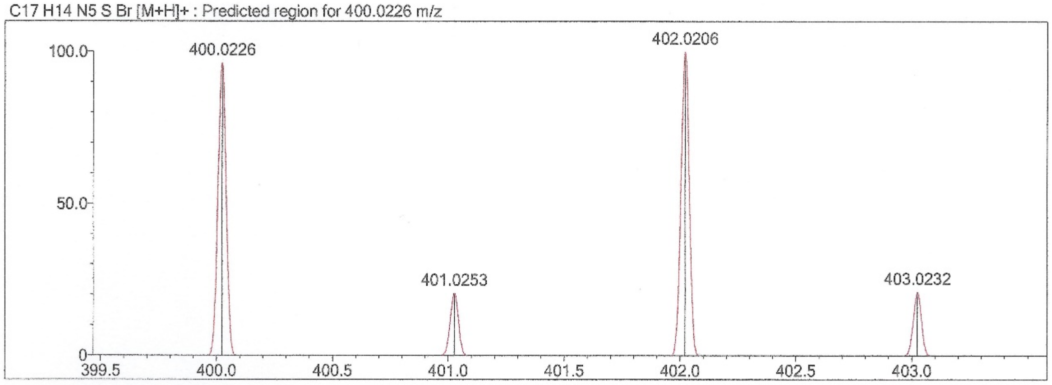

Rank	Score	Formula (M)	Ion	Meas. m/z	Pred. m/z	Df. (mDa)	Df. (ppm)	Iso	DBE
1	68.15	C17 H14 N5 S Br	[M+H]+	400.0238	400.0226	1.2	3.00	71.74	13.0

Figure S38: IR spectrum of compound **2g**

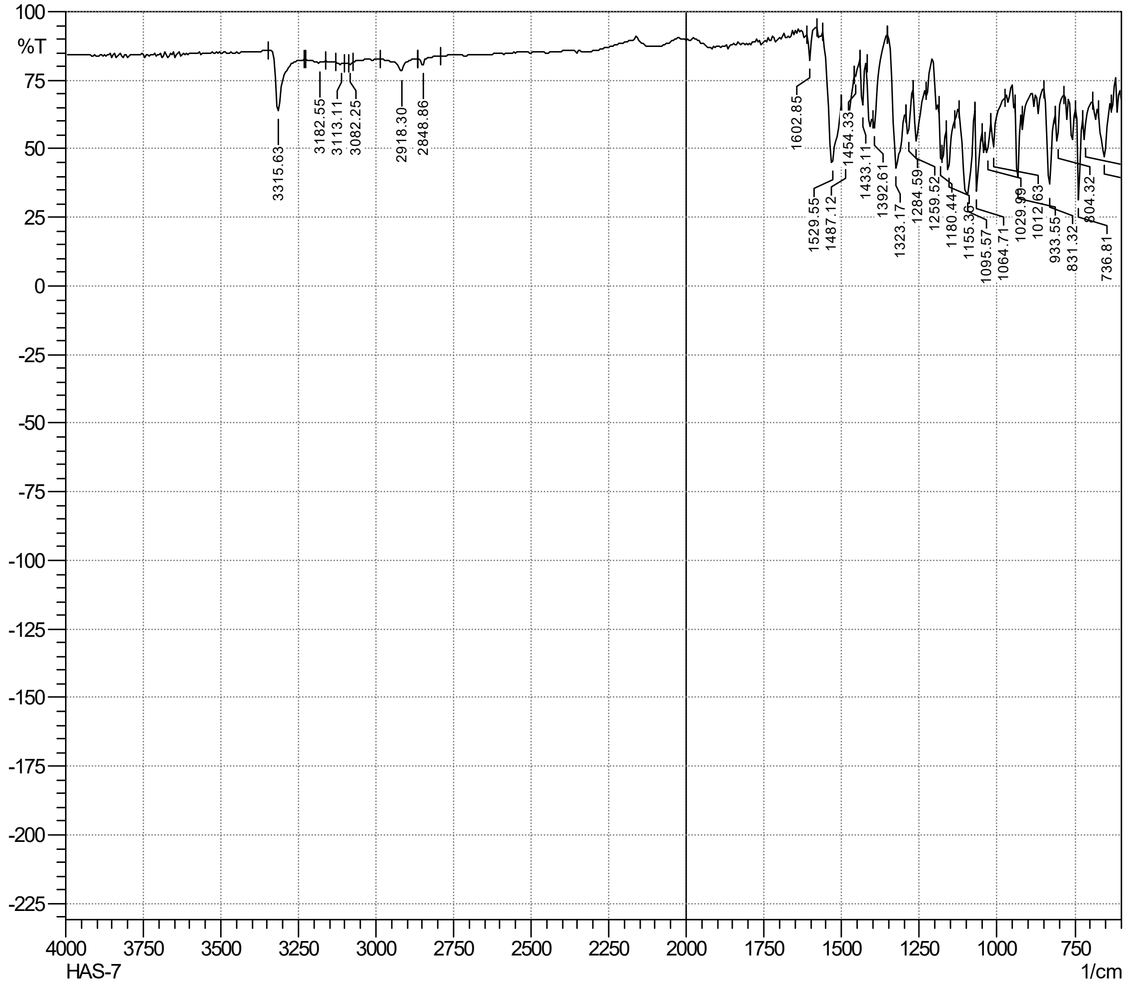

Figure S39: ^{1}H NMR spectrum of compound **2g**

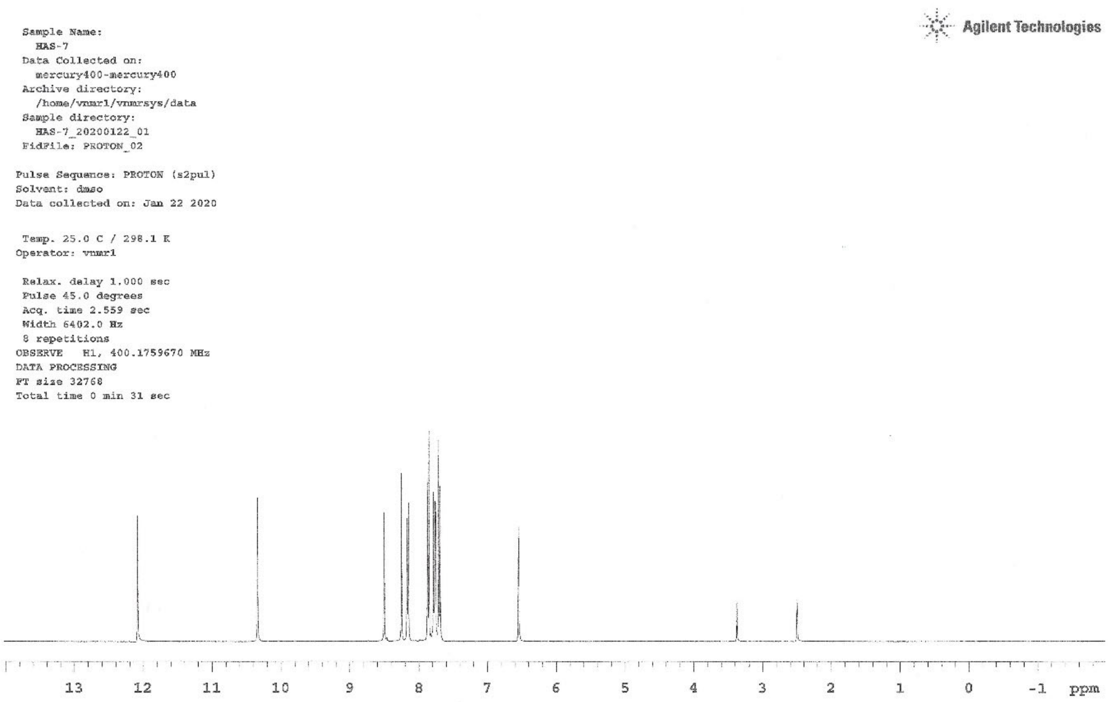

Figure S40: ^{1}H NMR spectrum of compound **2g** with integral values

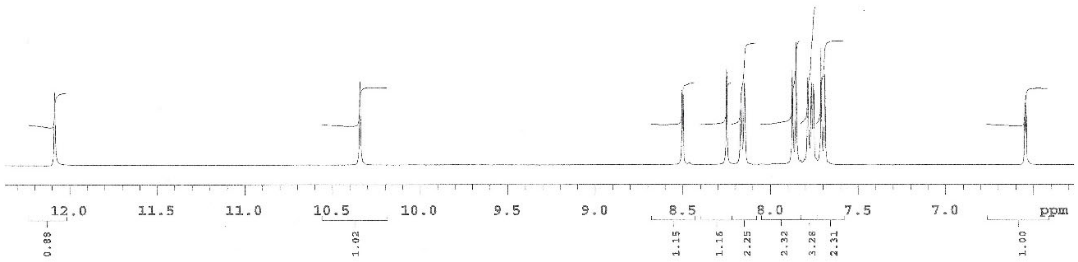

Figure S41: ^{1}H NMR spectrum of compound **2g** (6-12 ppm)

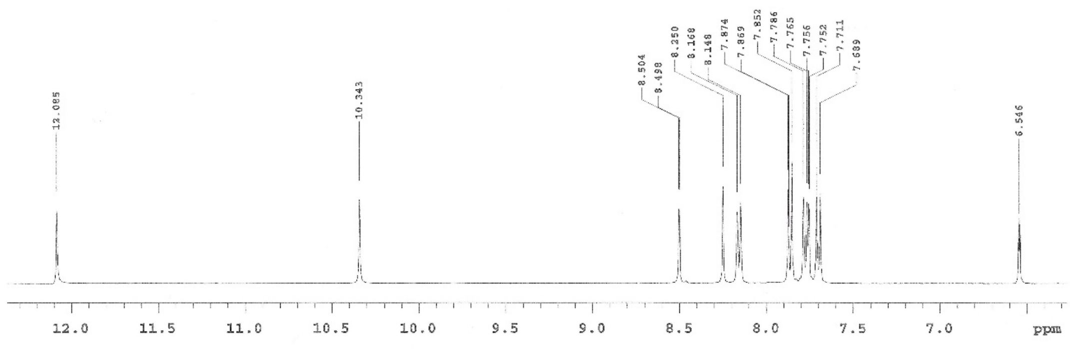

Figure S42: ^{13}C NMR spectrum of compound **2g**

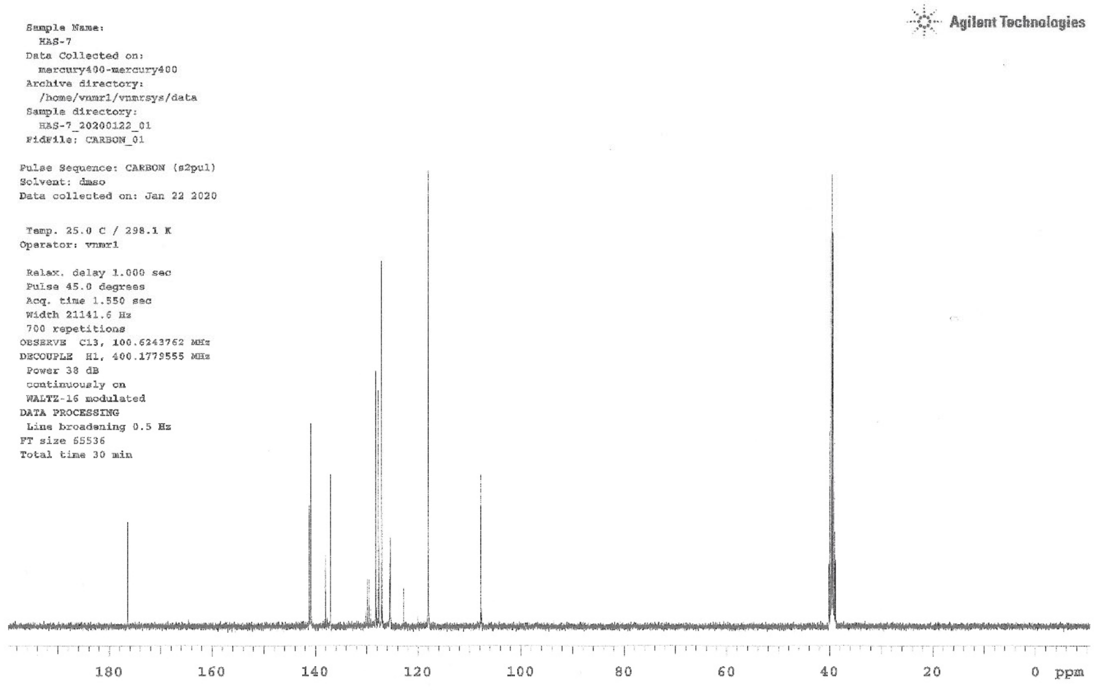

Figure S43: HRMS spectrum of compound **2g**

Formula Predictor Report - has-7_47.lcd

Data File: C:\LabSolutions\Data\Analiz\mdaltintop\has-7_47.lcd

Elmt	Val.	Min	Max	Elmt	Val.	Min	Max	Elmt	Val.	Min	Max	Elmt	Val.	Min	Max	Use Adduct
H	1	6	40	O	2	0	0	S	2	0	4	Ru	2	0	0	H
C	4	7	33	F	1	3	3	Cl	1	0	0	Pd	2	0	0	
N	3	3	7	P	3	0	0	Br	1	0	0	I	3	0	0	

Error Margin (ppm):	5	DBE Range:	5.0 - 20.0	Electron Ions:	both
HC Ratio:	unlimited	Apply N Rule:	yes	Use MSn Info:	yes
Max Isotopes:	3	Isotope RI (%):	1.00	Isotope Res:	9000
MSn Iso RI (%):	10.00	MSn Logic Mode:	AND	Max Results:	100

Event#: 1 MS(E+) Ret. Time : 4.733 -> 5.133 Scan# : 711 -> 771

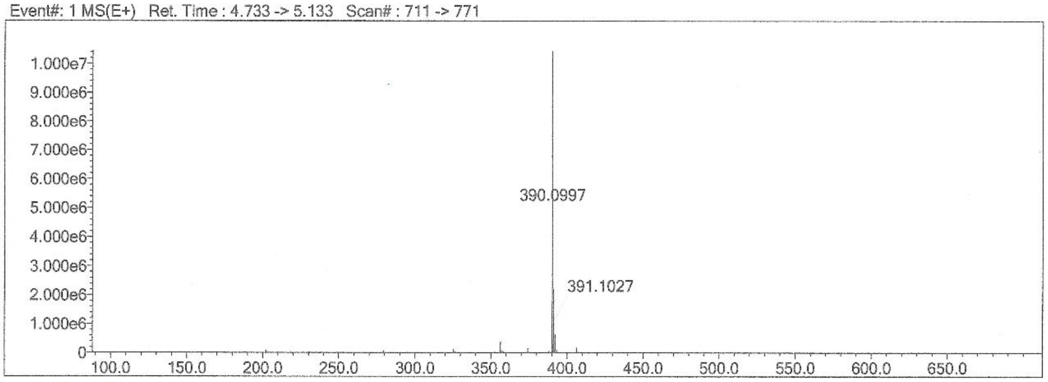

Measured region for 390.0997 m/z

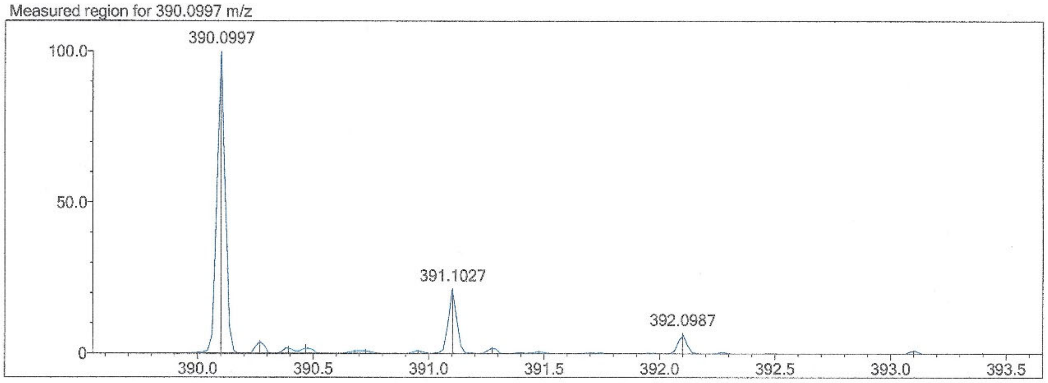

C18 H14 N5 F3 S [M+H]+ : Predicted region for 390.0995 m/z

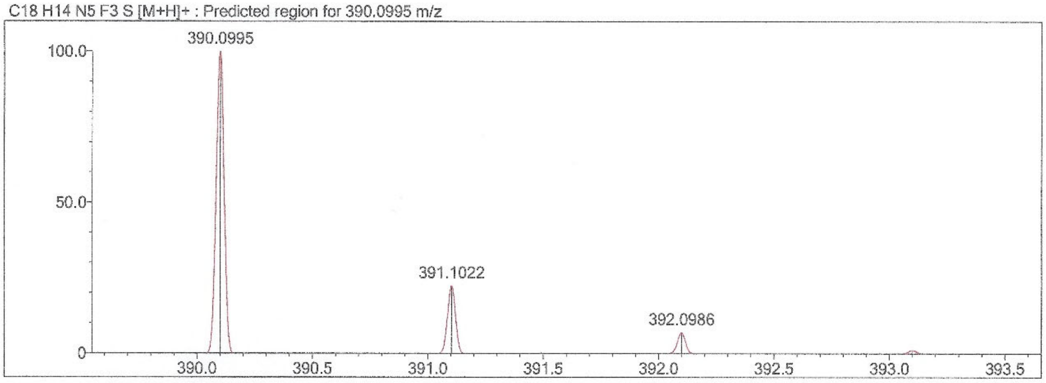

Rank	Score	Formula (M)	Ion	Meas. m/z	Pred. m/z	Df. (mDa)	Df. (ppm)	Iso	DBE
1	100.00	C18 H14 N5 F3 S	[M+H]+	390.0997	390.0995	0.2	0.51	100.00	13.0

Figure S44: IR spectrum of compound **2h**

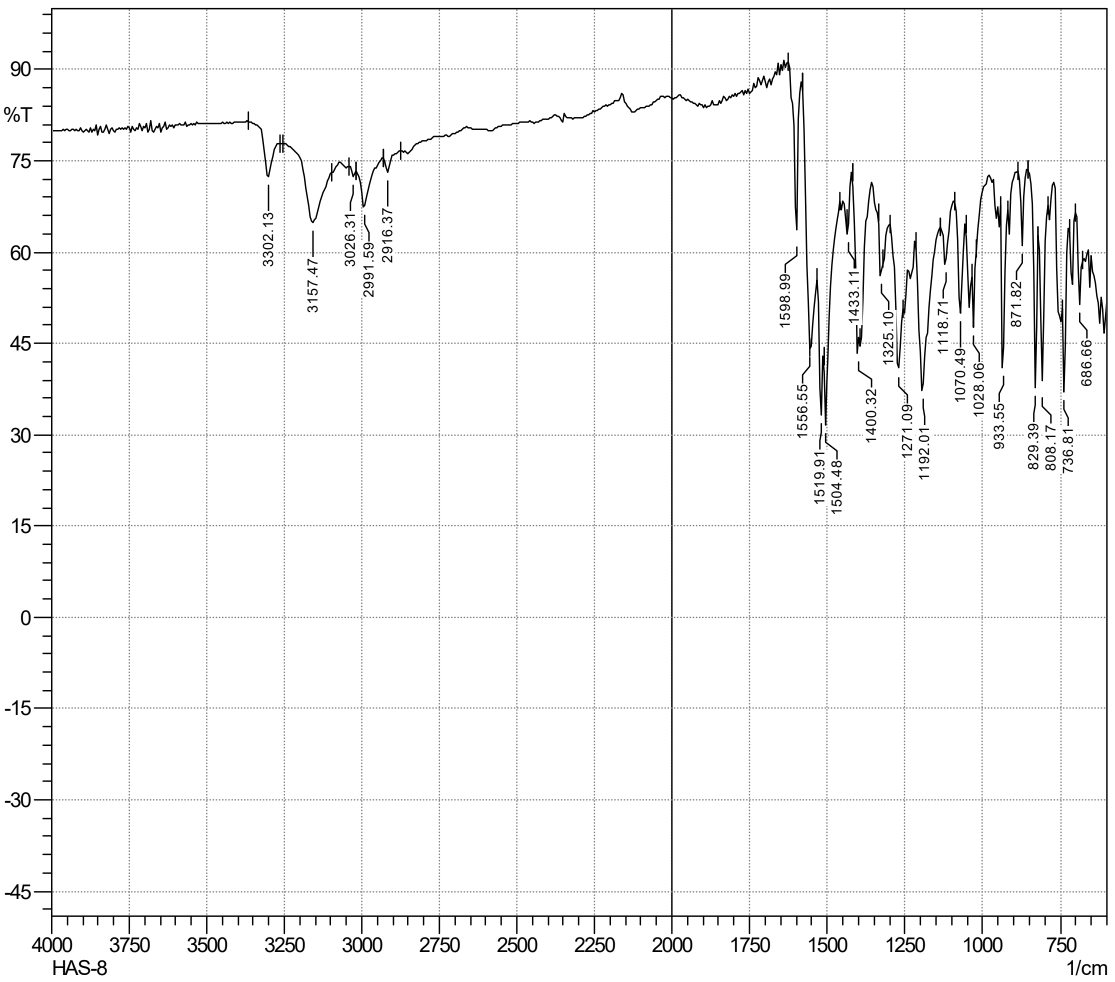

Figure S45: ^{1}H NMR spectrum of compound **2h**

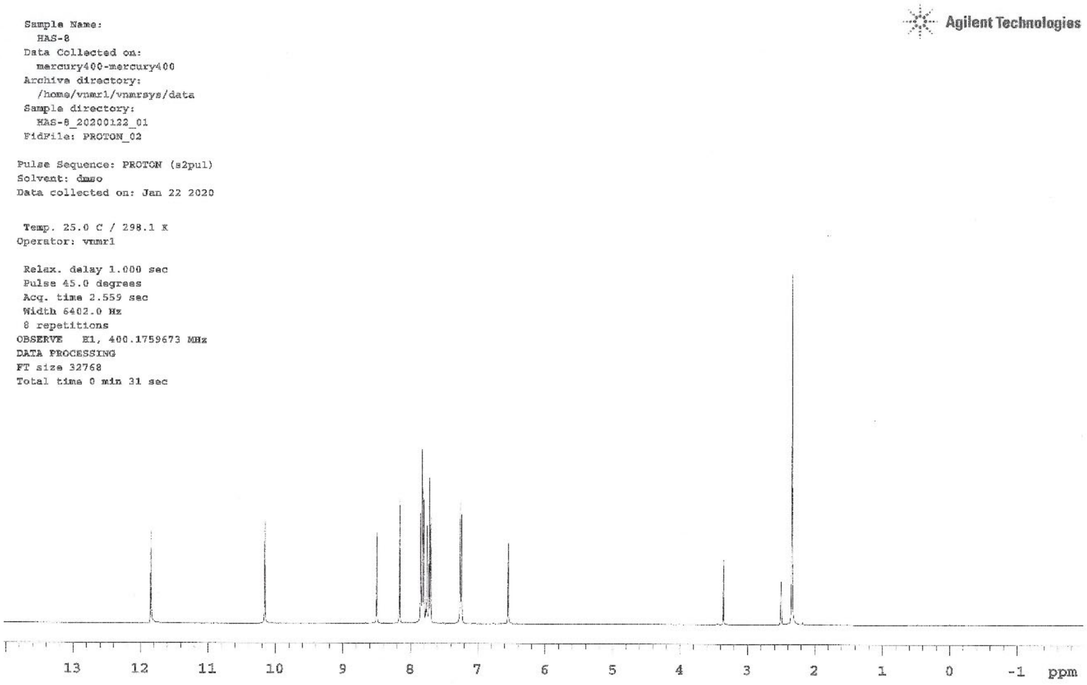

Figure S46: ^{1}H NMR spectrum of compound **2h** with integral values

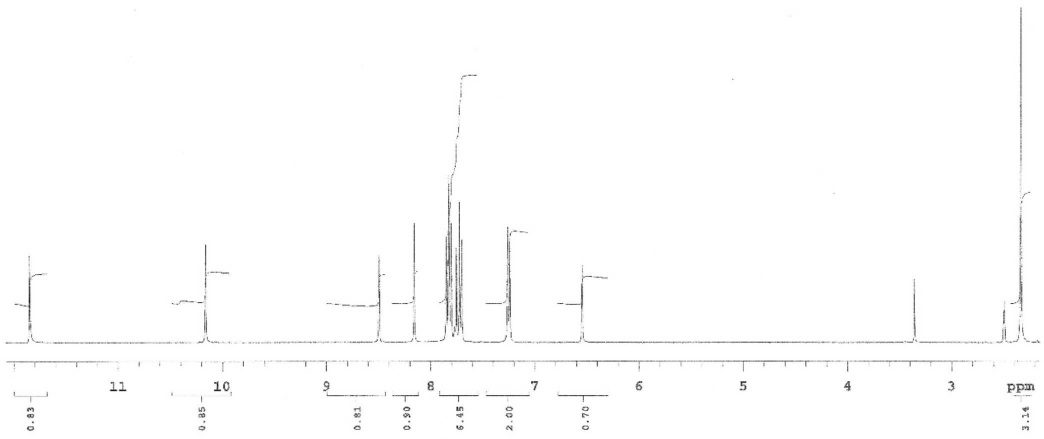

Figure S47: ^{1}H NMR spectrum of compound **2h** (2.3-12 ppm)

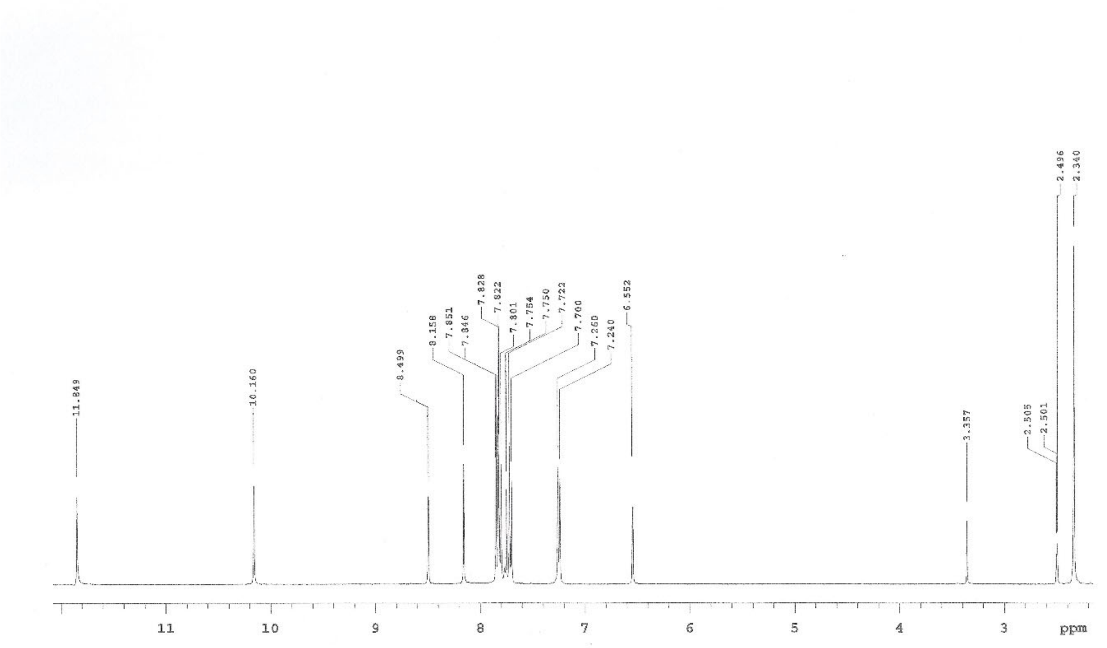

Figure S48: ^{13}C NMR spectrum of compound **2h**

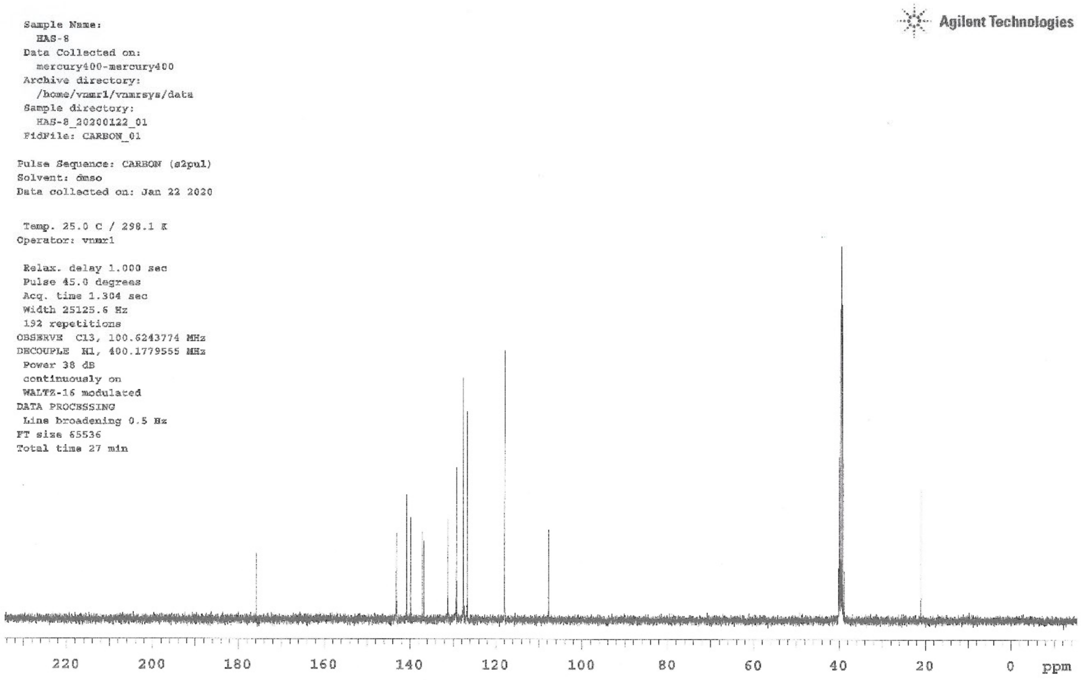

Figure S49: HRMS spectrum of compound **2h**

Formula Predictor Report - has-8_48.lcd

Page 1 of 1

Data File: C:\LabSolutions\Data\Analiz\mdaltintop\has-8_48.lcd

Elmt	Val.	Min	Max	Elmt	Val.	Min	Max	Elmt	Val.	Min	Max	Elmt	Val.	Min	Max	Use Adduct
H	1	6	40	O	2	0	0	S	2	0	4	Ru	2	0	0	H
C	4	7	33	F	1	0	3	Cl	1	0	0	Pd	2	0	0	
N	3	3	7	P	3	0	0	Br	1	0	0	I	3	0	0	

Error Margin (ppm): 5
HC Ratio: unlimited
Max Isotopes: 3
MSn Iso RI (%): 10.00

DBE Range: 5.0 - 20.0
Apply N Rule: yes
Isotope RI (%): 1.00
MSn Logic Mode: AND

Electron Ions: both
Use MSn Info: yes
Isotope Res: 9000
Max Results: 100

Event#: 1 MS(E+) Ret. Time : 4.587 Scan# : 689

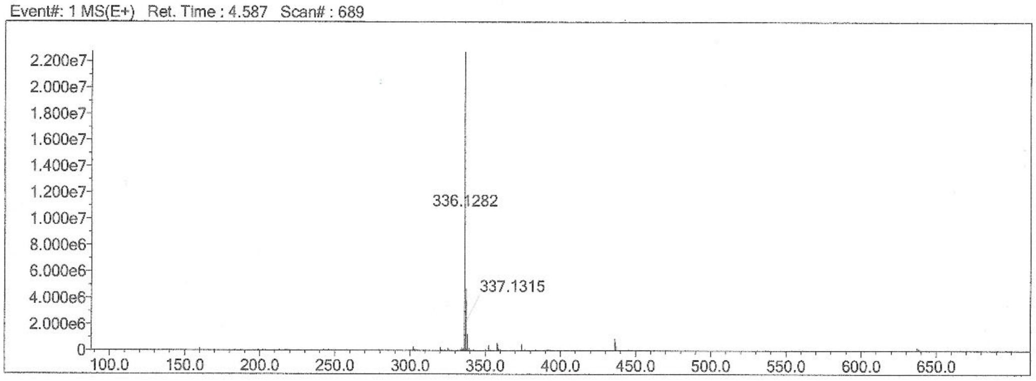

Measured region for 336.1282 m/z

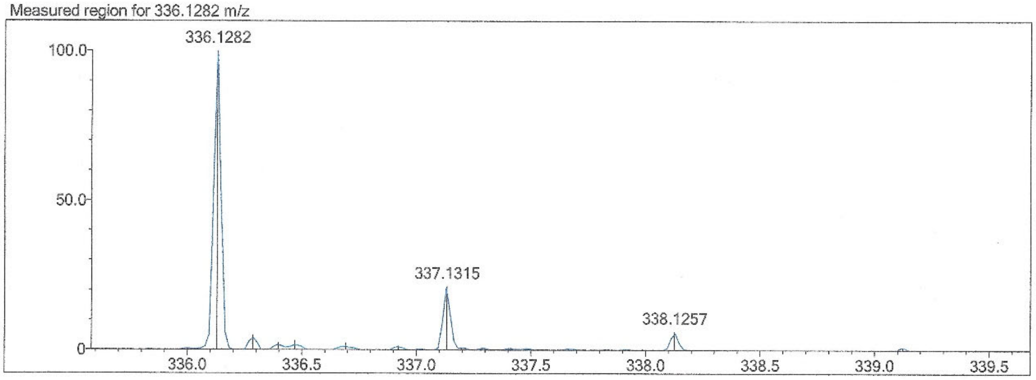

C18 H17 N5 S [M+H]+ : Predicted region for 336.1277 m/z

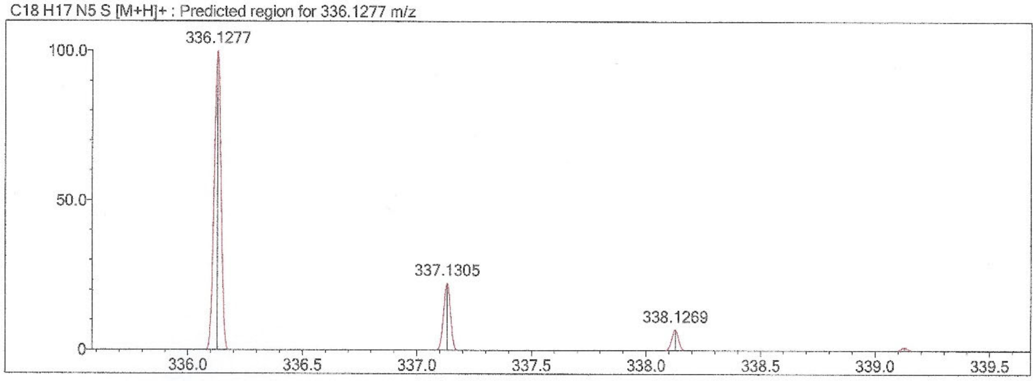

Rank	Score	Formula (M)	Ion	Meas. m/z	Pred. m/z	Df. (mDa)	Df. (ppm)	Iso	DBE
1	84.38	C18 H17 N5 S	[M+H]+	336.1282	336.1277	0.5	1.49	85.43	13.0

Figure S50: IR spectrum of compound **2i**

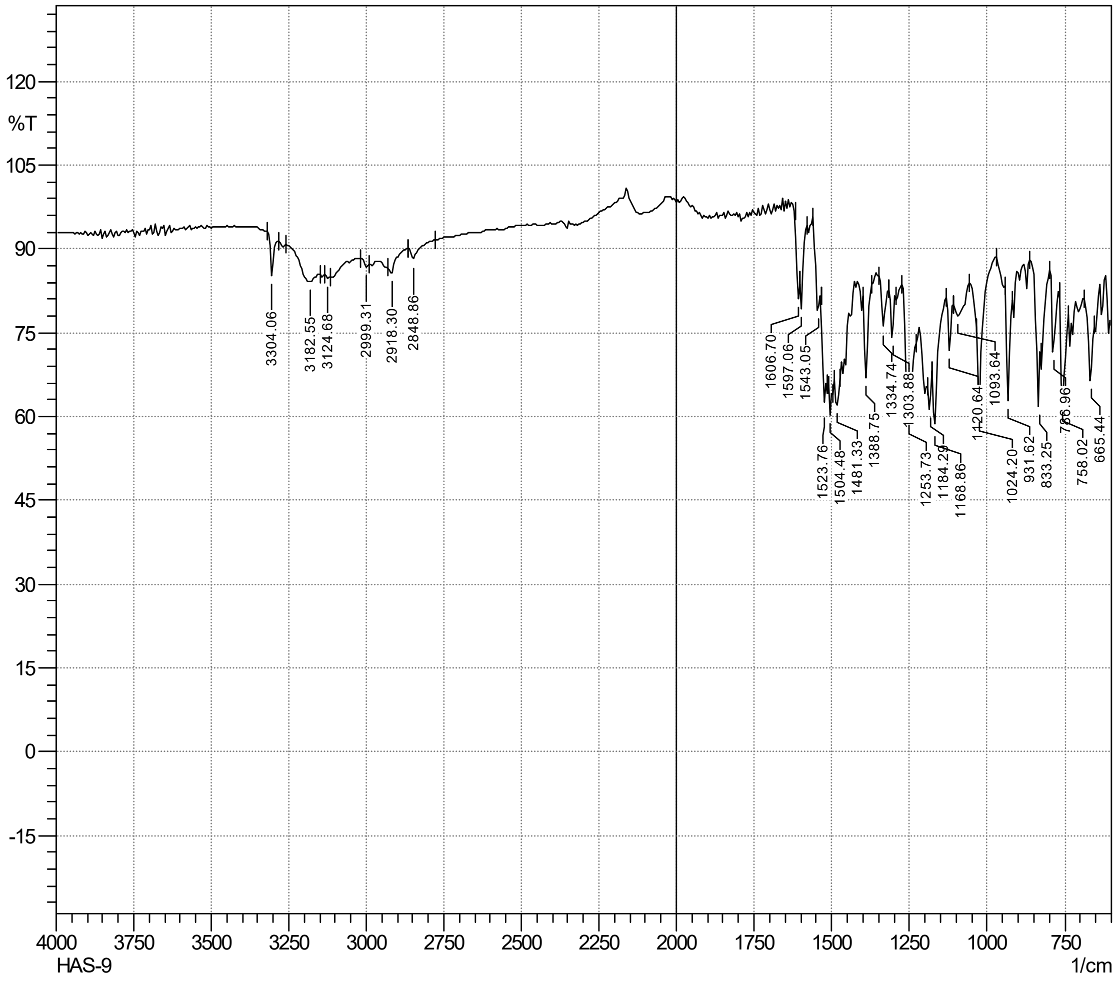

Figure S51: ^{1}H NMR spectrum of compound **2i**

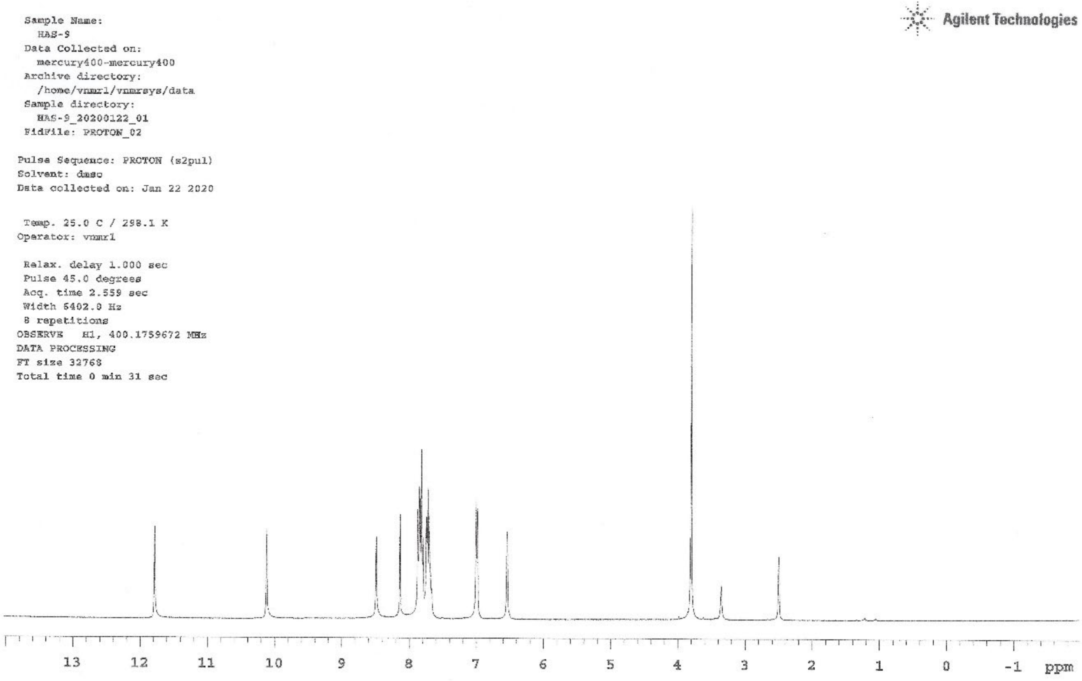

Figure S52: ^{1}H NMR spectrum of compound **2i** with integral values

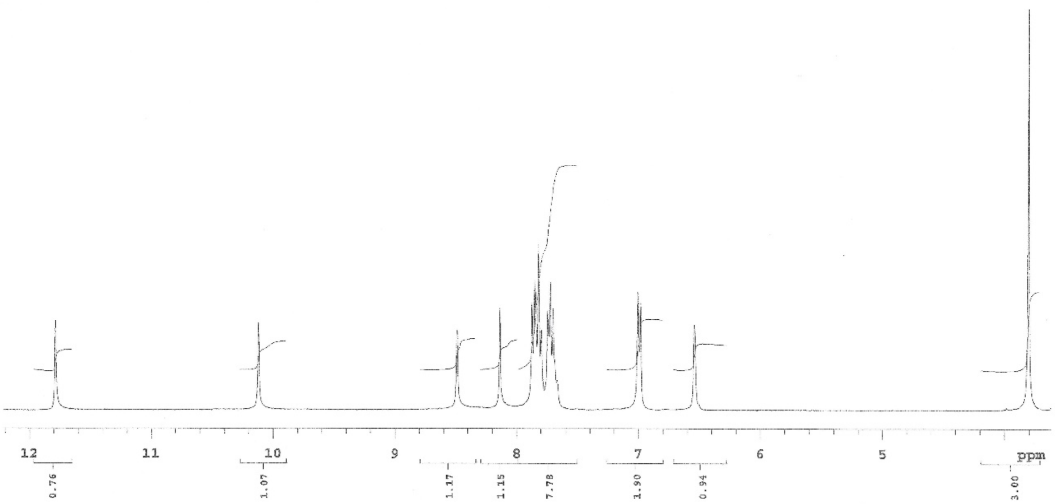

Figure S53: ^{1}H NMR spectrum of compound **2i** (3.8-12 ppm)

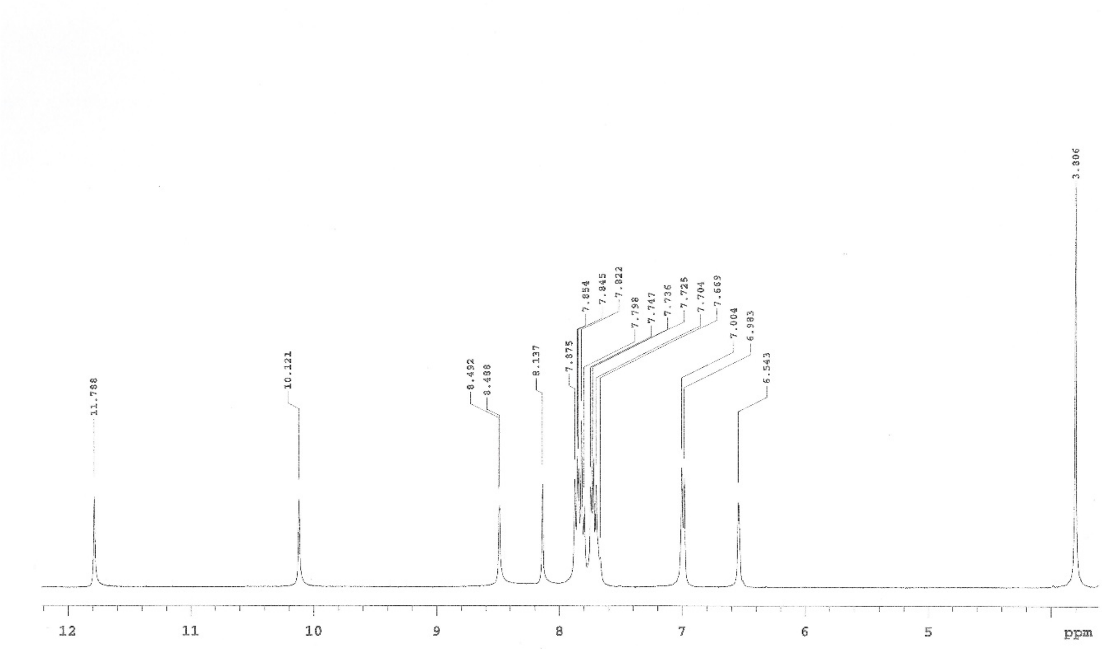

Figure S54: ^{13}C NMR spectrum of compound **2i**

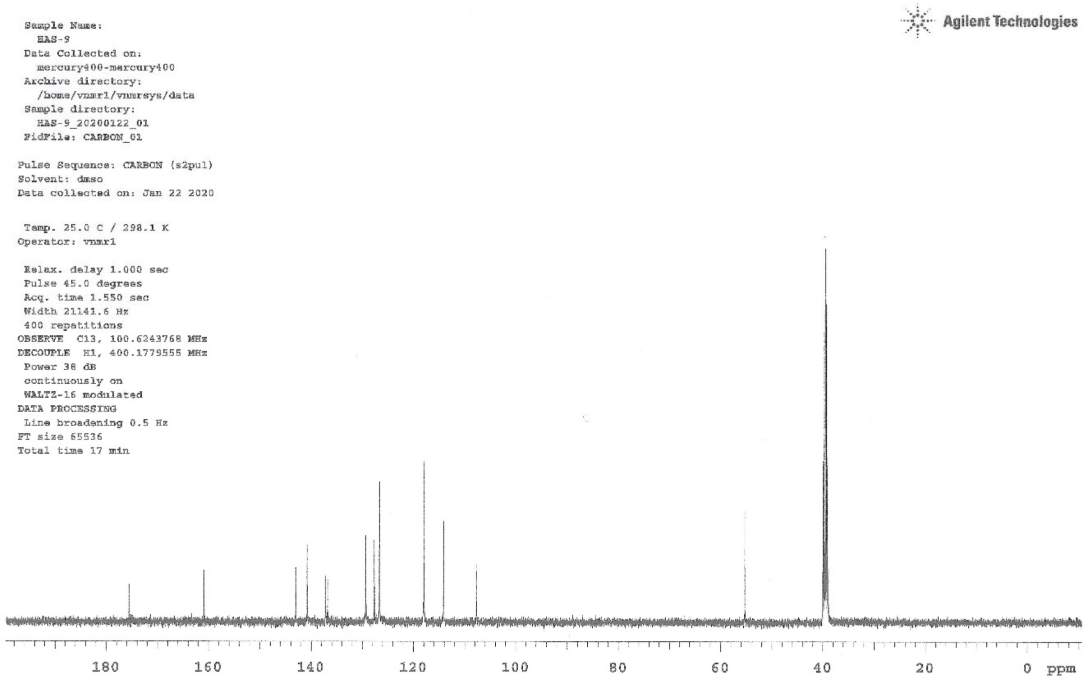

Figure S55: HRMS spectrum of compound **2i**

Formula Predictor Report - has-9_50.lcd

Data File: C:\LabSolutions\Data\Analiz\mdaltintop\has-9_50.lcd

Elmt	Val.	Min	Max	Elmt	Val.	Min	Max	Elmt	Val.	Min	Max	Elmt	Val.	Min	Max	Use Adduct
H	1	6	40	O	2	0	2	S	2	0	2	Ru	2	0	0	H
C	4	7	33	F	1	0	0	Cl	1	0	0	Pd	2	0	0	
N	3	3	7	P	3	0	0	Br	1	0	0	I	3	0	0	

Error Margin (ppm): 5
HC Ratio: unlimited
Max Isotopes: 3
MSn Iso RI (%): 10.00

DBE Range: 5.0 - 20.0
Apply N Rule: yes
Isotope RI (%): 1.00
MSn Logic Mode: AND

Electron Ions: both
Use MSn Info: yes
Isotope Res: 9000
Max Results: 100

Event#: 1 MS(E+) Ret. Time : 3.987 -> 4.293 Scan# : 599 -> 645

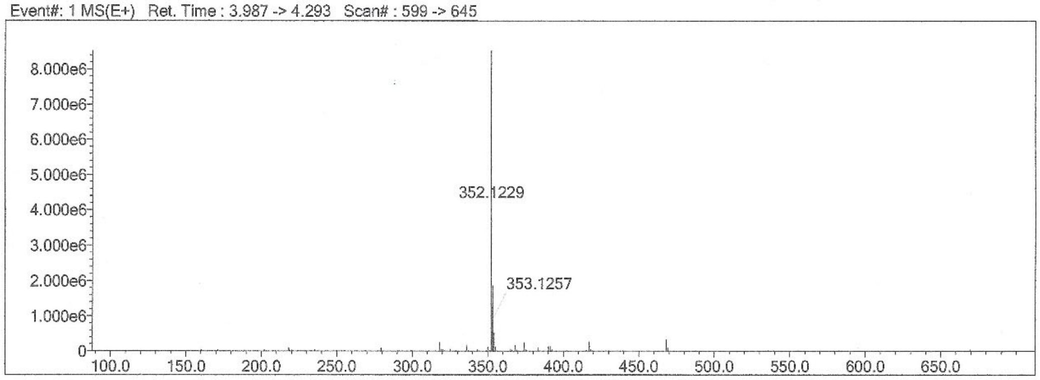

Measured region for 352.1229 m/z

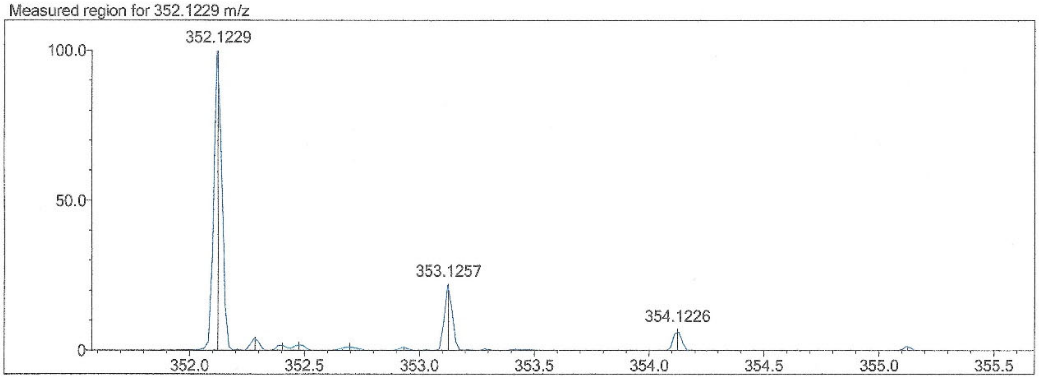

C18 H17 N5 O S [M+H]+ : Predicted region for 352.1227 m/z

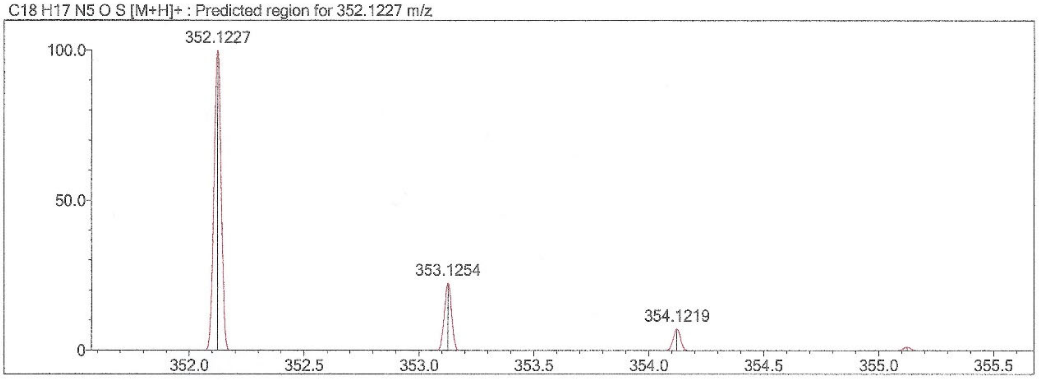

Rank	Score	Formula (M)	Ion	Meas. m/z	Pred. m/z	Df. (mDa)	Df. (ppm)	Iso	DBE
1	83.85	C18 H17 N5 O S	[M+H]+	352.1229	352.1227	0.2	0.57	83.85	13.0

Figure S56: IR spectrum of compound **2j**

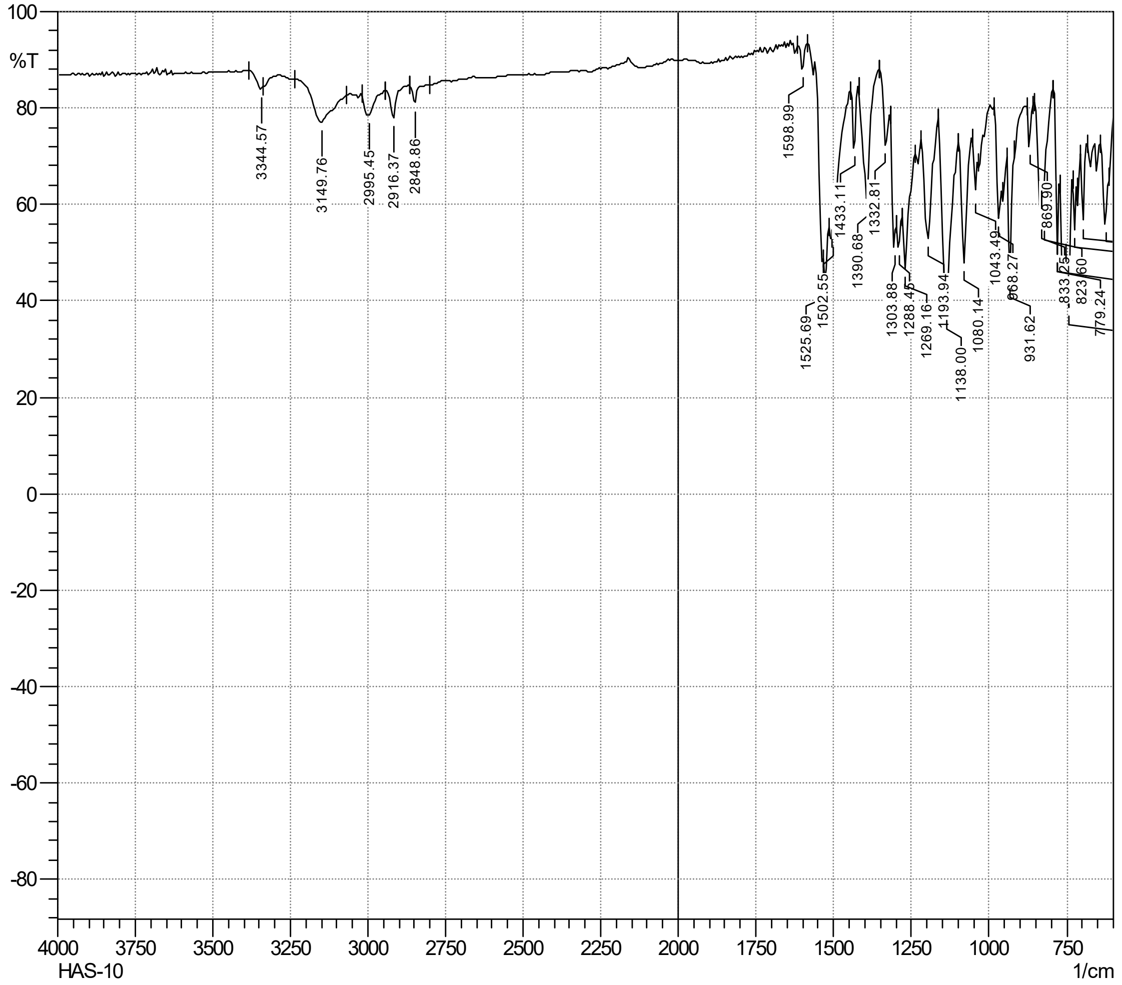

Figure S57: ^{1}H NMR spectrum of compound **2j**

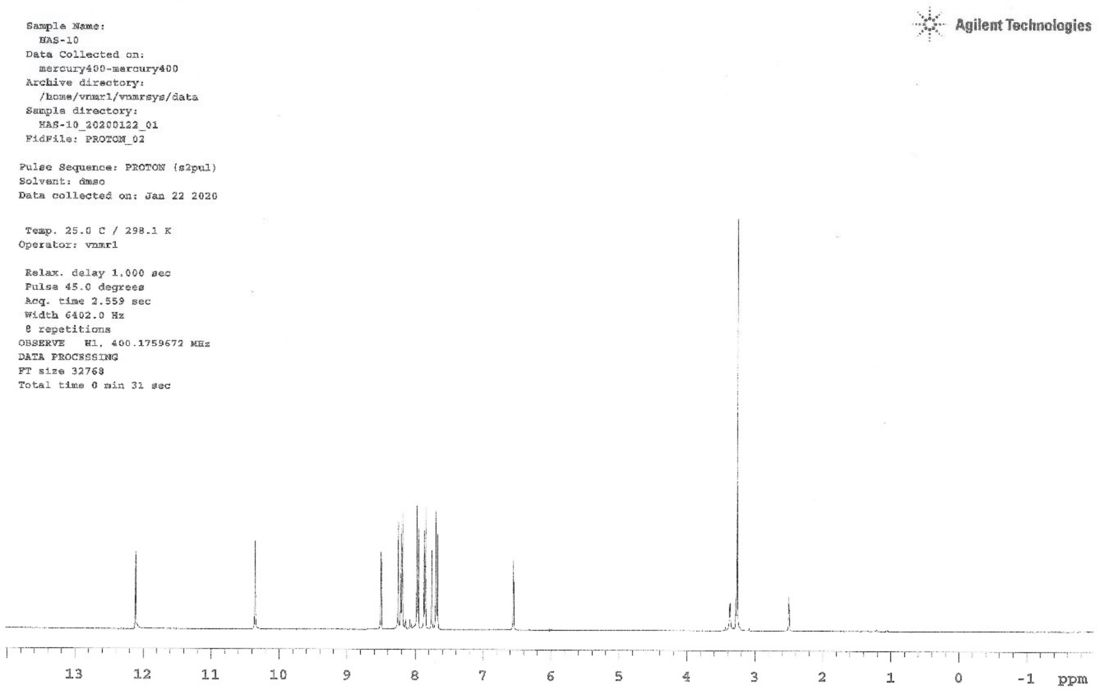

Figure S58: ^{1}H NMR spectrum of compound **2j** with integral values

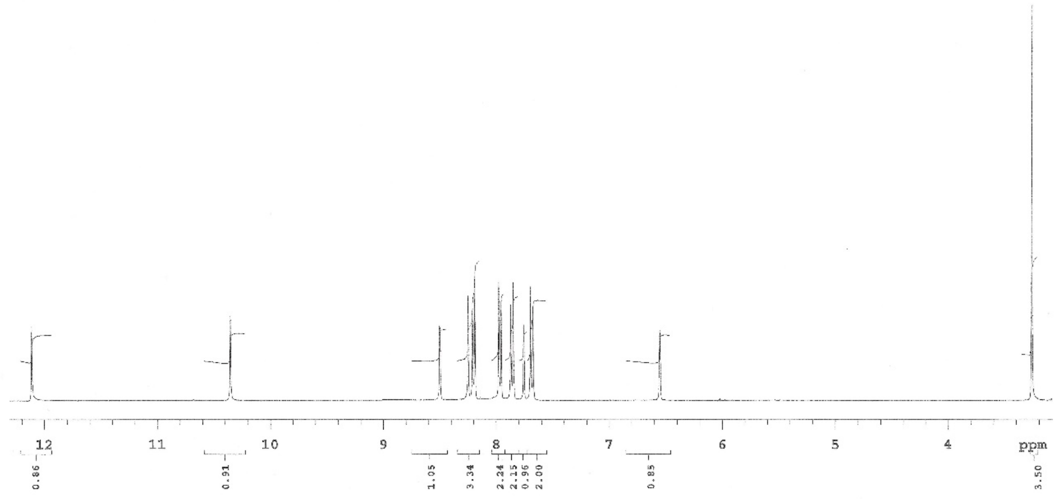

Figure S59: ^{1}H NMR spectrum of compound **2j** (3.2-12 ppm)

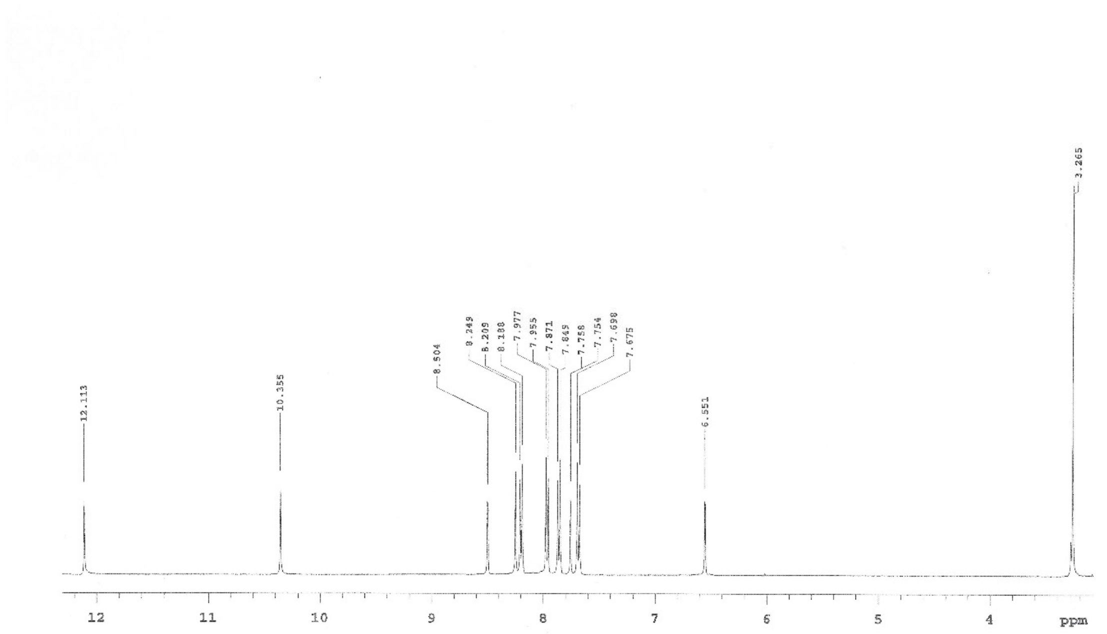

Figure S60: ^{13}C NMR spectrum of compound **2j**

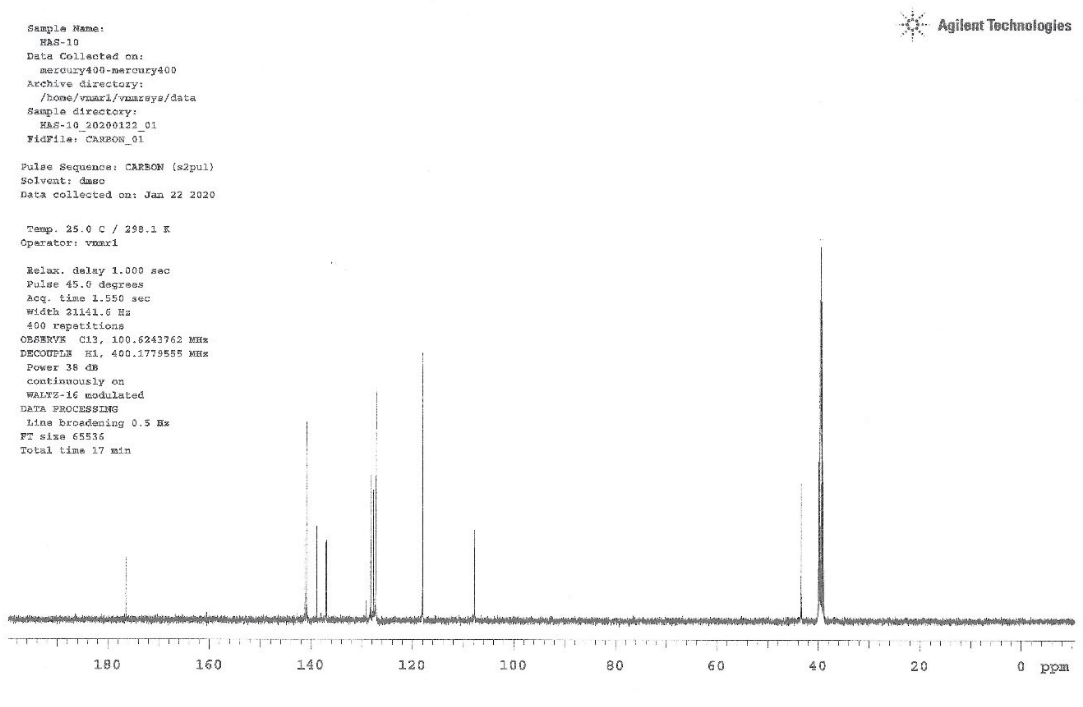

Figure S61: HRMS spectrum of compound **2j**

Formula Predictor Report - has-10_51.lcd

Page 1 of 1

Data File: C:\LabSolutions\Data\Analiz\mdaltintop\has-10_51.lcd

Elmt	Val.	Min	Max	Elmt	Val.	Min	Max	Elmt	Val.	Min	Max	Elmt	Val.	Min	Max	Use Adduct
H	1	6	40	O	2	0	2	S	2	0	2	Ru	2	0	0	H
C	4	7	33	F	1	0	0	Cl	1	0	0	Pd	2	0	0	
N	3	3	7	P	3	0	0	Br	1	0	0	I	3	0	0	

Error Margin (ppm): 5
HC Ratio: unlimited
Max Isotopes: 3
MSn Iso RI (%): 10.00

DBE Range: 5.0 - 20.0
Apply N Rule: yes
Isotope RI (%): 1.00
MSn Logic Mode: AND

Electron Ions: both
Use MSn Info: yes
Isotope Res: 9000
Max Results: 100

Event#: 1 MS(E+) Ret. Time : 2.280 -> 2.627 Scan# : 343 -> 395

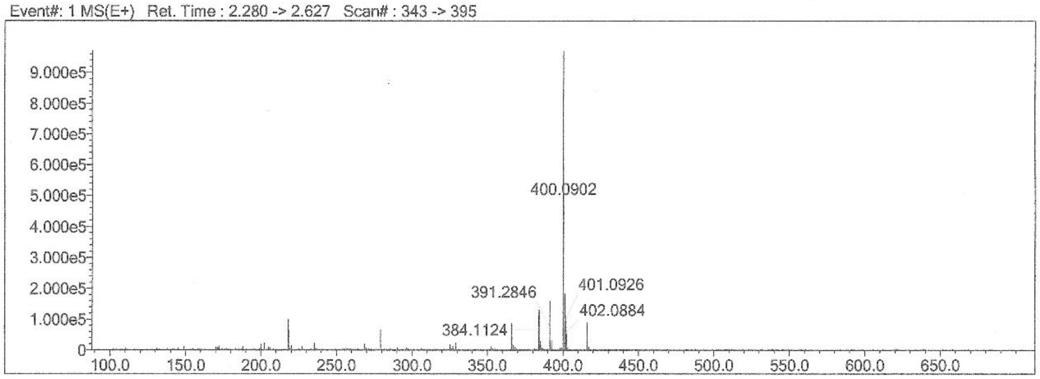

Measured region for 400.0902 m/z

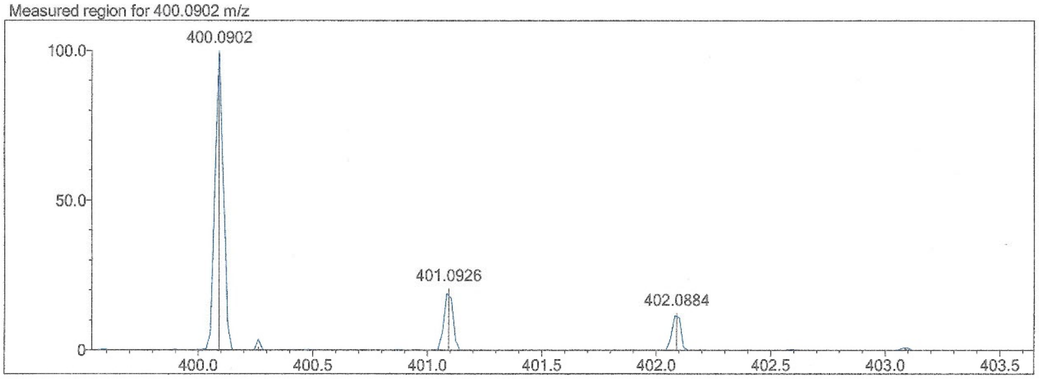

C18 H17 N5 O2 S2 [M+H]+ : Predicted region for 400.0896 m/z

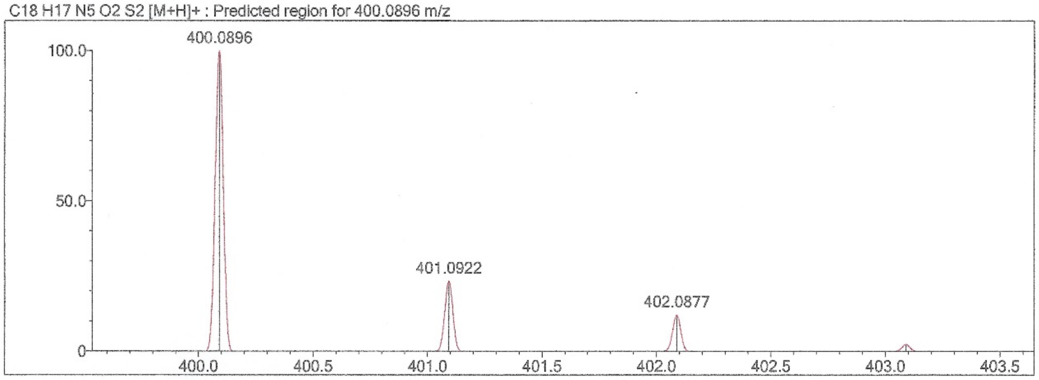

Rank	Score	Formula (M)	Ion	Meas. m/z	Pred. m/z	Df. (mDa)	Df. (ppm)	Iso	DBE
1	86.03	C18 H17 N5 O2 S2	[M+H]+	400.0902	400.0896	0.6	1.50	87.12	13.0

Figure S62: IR spectrum of compound **2k**

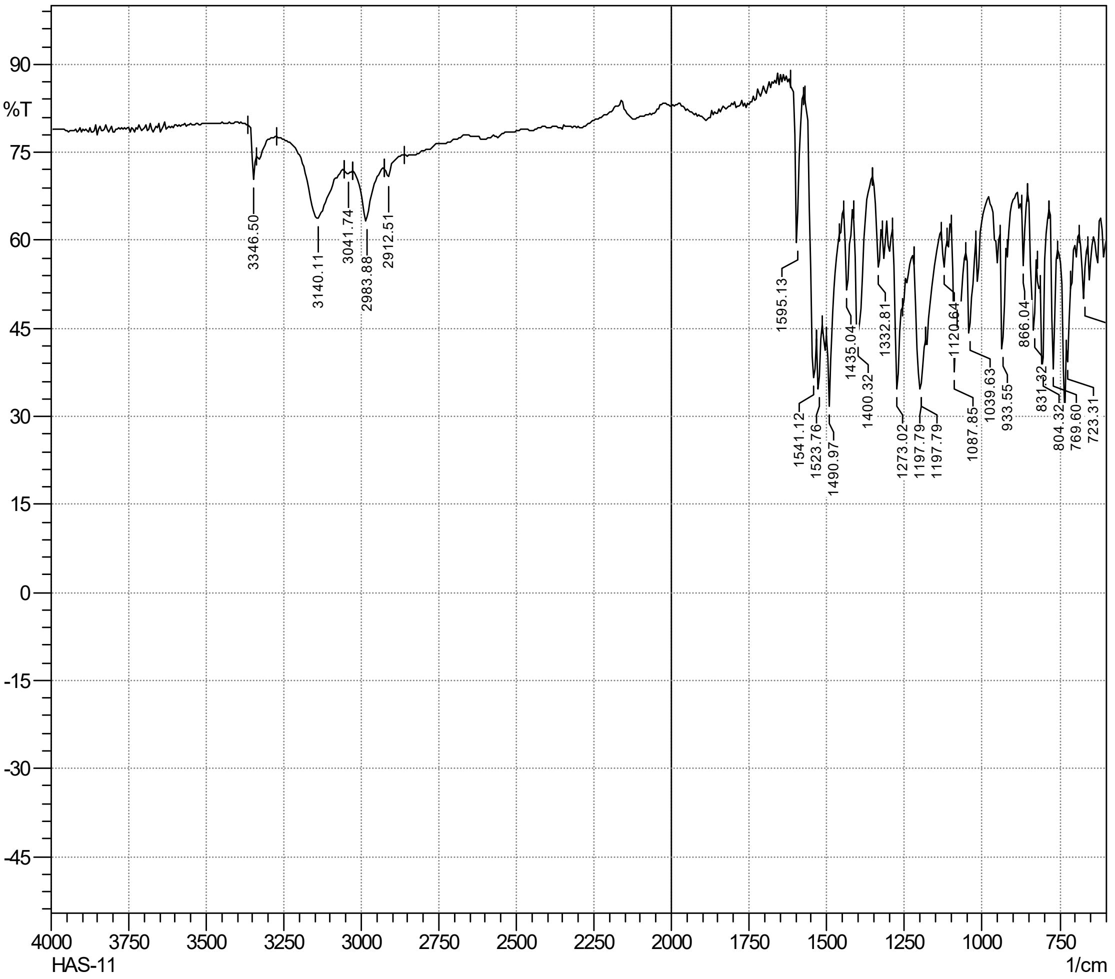

Figure S63: ^{1}H NMR spectrum of compound **2k**

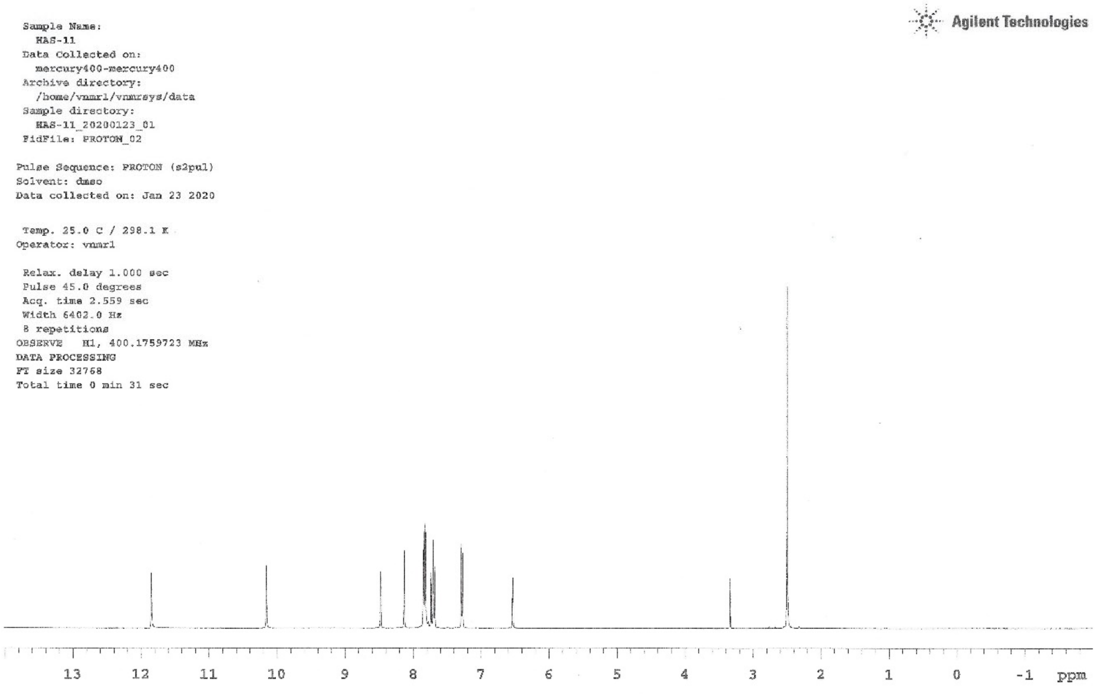

Figure S64: ^{1}H NMR spectrum of compound **2k** with integral values

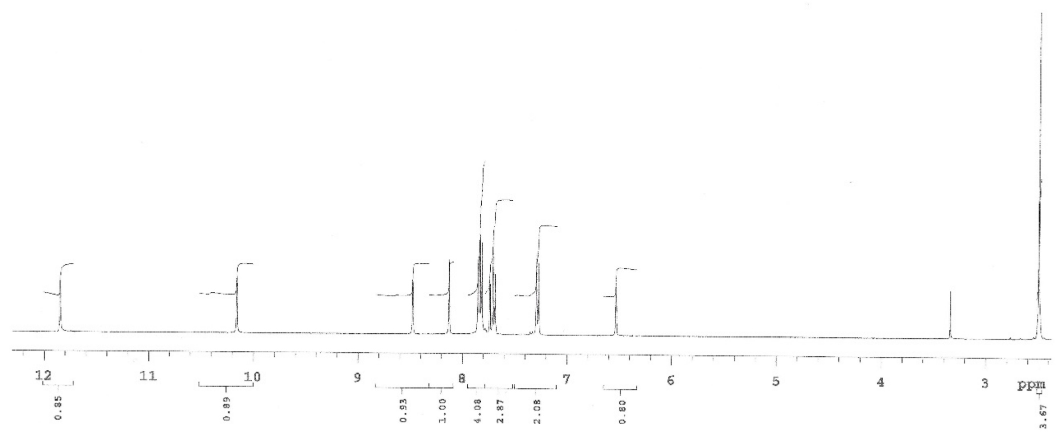

Figure S65: ^{1}H NMR spectrum of compound **2k** (2.5-12 ppm)

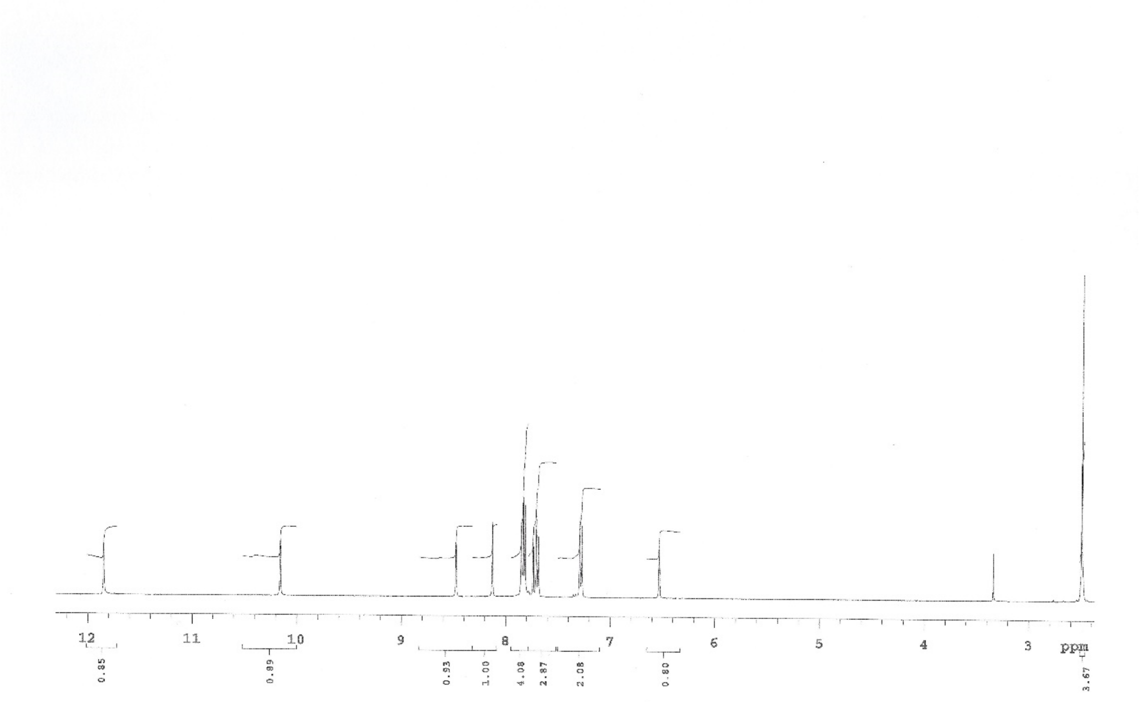

Figure S66: ^{13}C NMR spectrum of compound **2k**

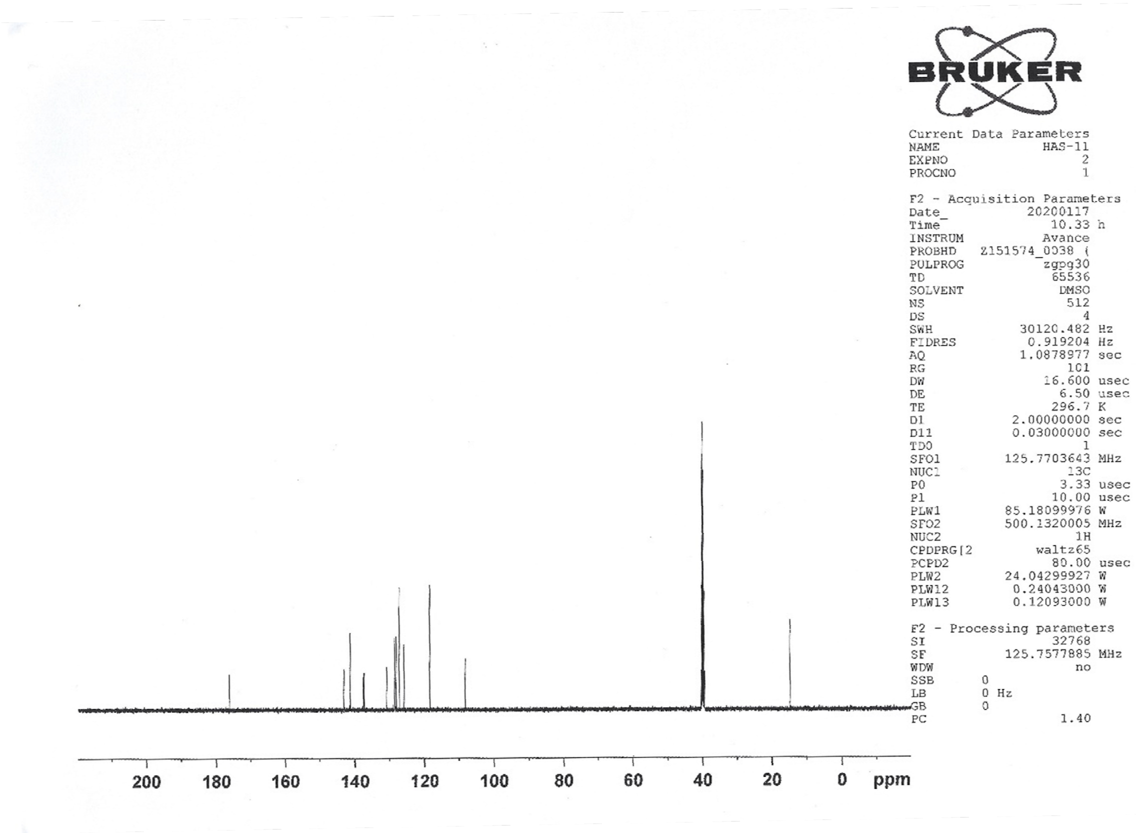

Figure S67: HRMS spectrum of compound **2k**

Formula Predictor Report - has-11_52.lcd

Page 1 of 1

Data File: C:\LabSolutions\Data\Analiz\mdaltintop\has-11_52.lcd

Elmt	Val.	Min	Max	Elmt	Val.	Min	Max	Elmt	Val.	Min	Max	Elmt	Val.	Min	Max	Use Adduct
H	1	6	40	O	2	0	2	S	2	0	2	Ru	2	0	0	H
C	4	7	33	F	1	0	0	Cl	1	0	0	Pd	2	0	0	
N	3	3	7	P	3	0	0	Br	1	0	0	I	3	0	0	

Error Margin (ppm):	5	DBE Range:	5.0 - 20.0	Electron Ions:	both
HC Ratio:	unlimited	Apply N Rule:	yes	Use MSn Info:	yes
Max Isotopes:	3	Isotope RI (%):	1.00	Isotope Res:	9000
MSn Iso RI (%):	10.00	MSn Logic Mode:	AND	Max Results:	100

Event#: 1 MS(E+) Ret. Time : 4.413 -> 4.760 Scan# : 663 -> 715

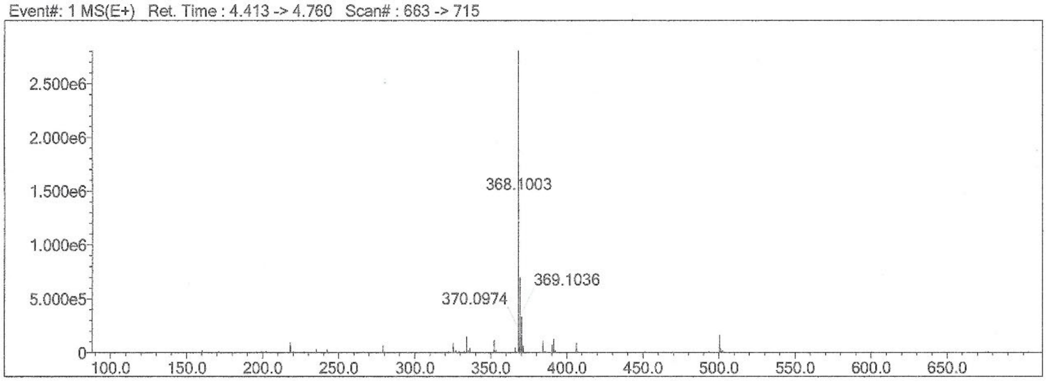

Measured region for 368.1003 m/z

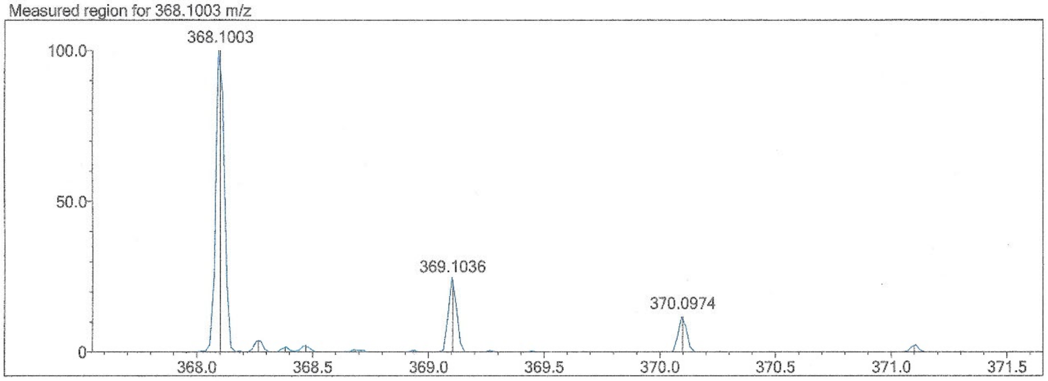

C18 H17 N5 S2 [M+H]+ : Predicted region for 368.0998 m/z

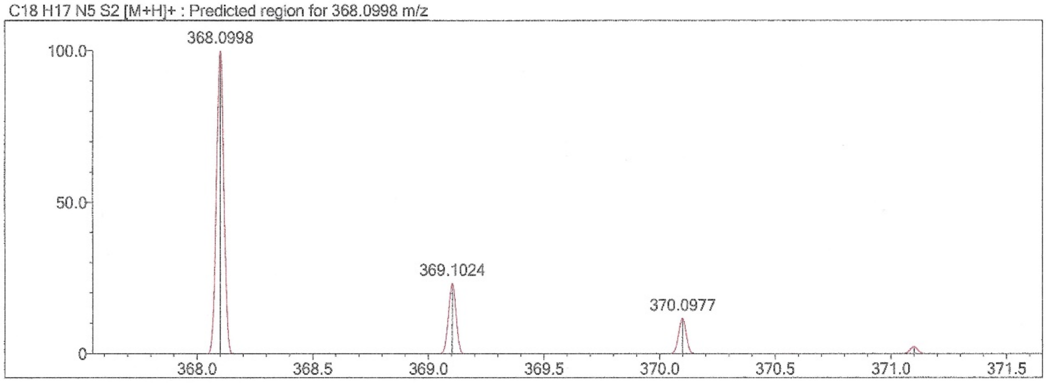

Rank	Score	Formula (M)	Ion	Meas. m/z	Pred. m/z	Df. (mDa)	Df. (ppm)	Iso	DBE
1	90.24	C18 H17 N5 S2	[M+H]+	368.1003	368.0998	0.5	1.36	91.06	13.0

Figure S68: IR spectrum of compound **2l**

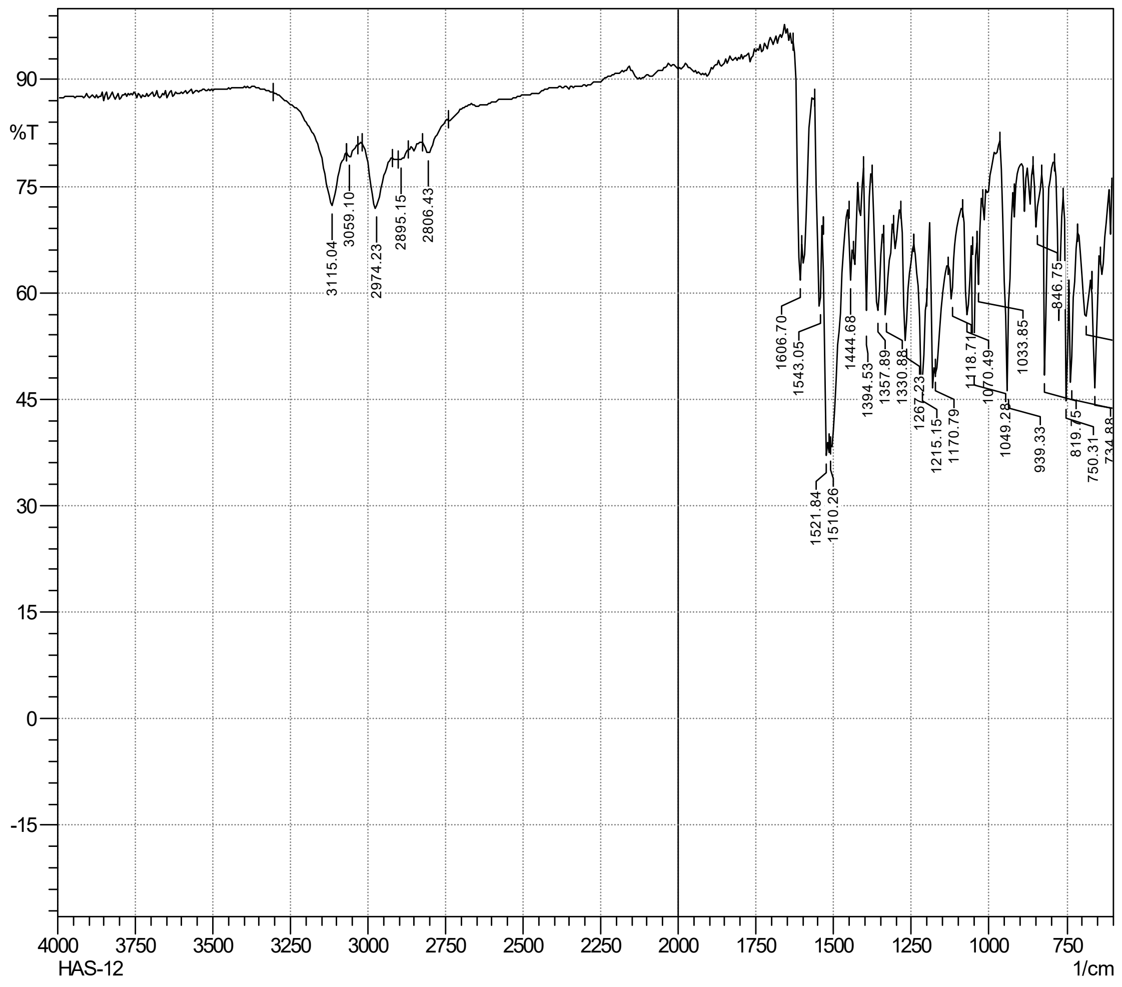

Figure S69: ^{1}H NMR spectrum of compound **2l**

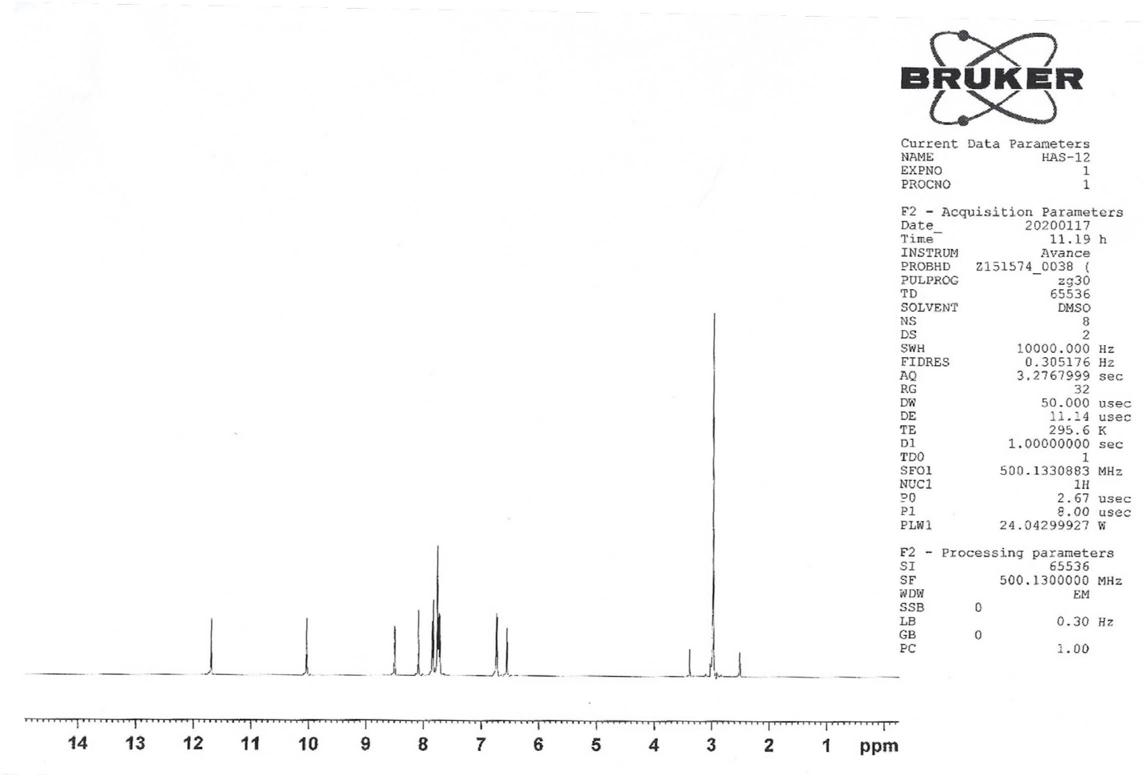

Figure S70: ^{1}H NMR spectrum of compound **2l** with integral values

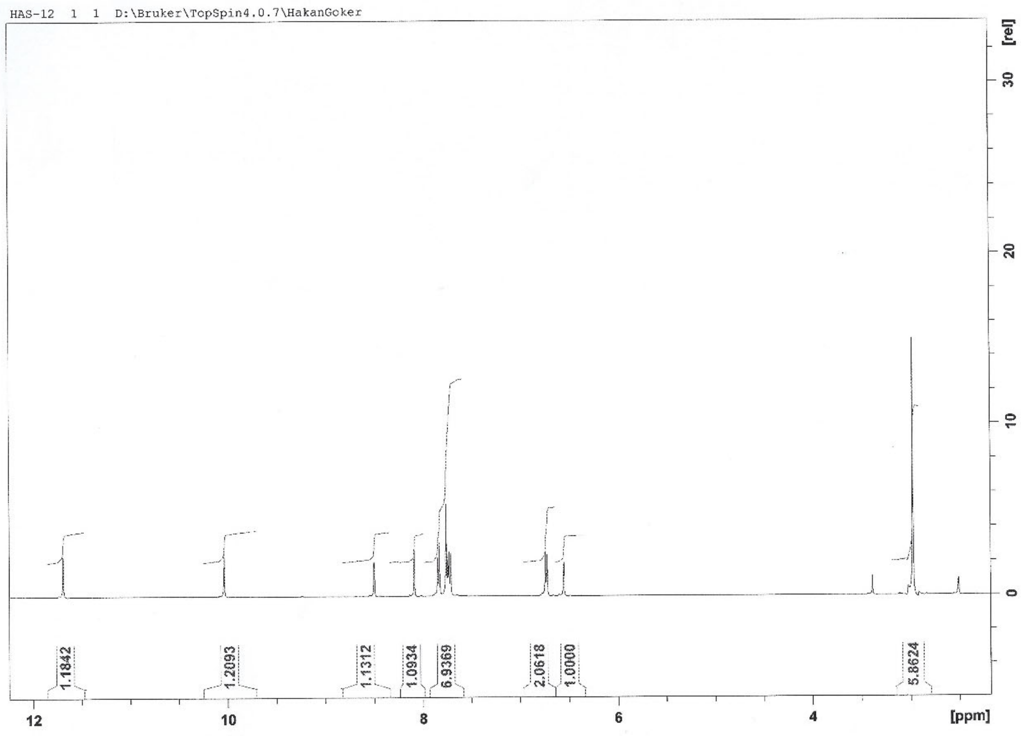

Figure S71: ^{1}H NMR spectrum of compound **2l** (2.5-3.5 ppm)

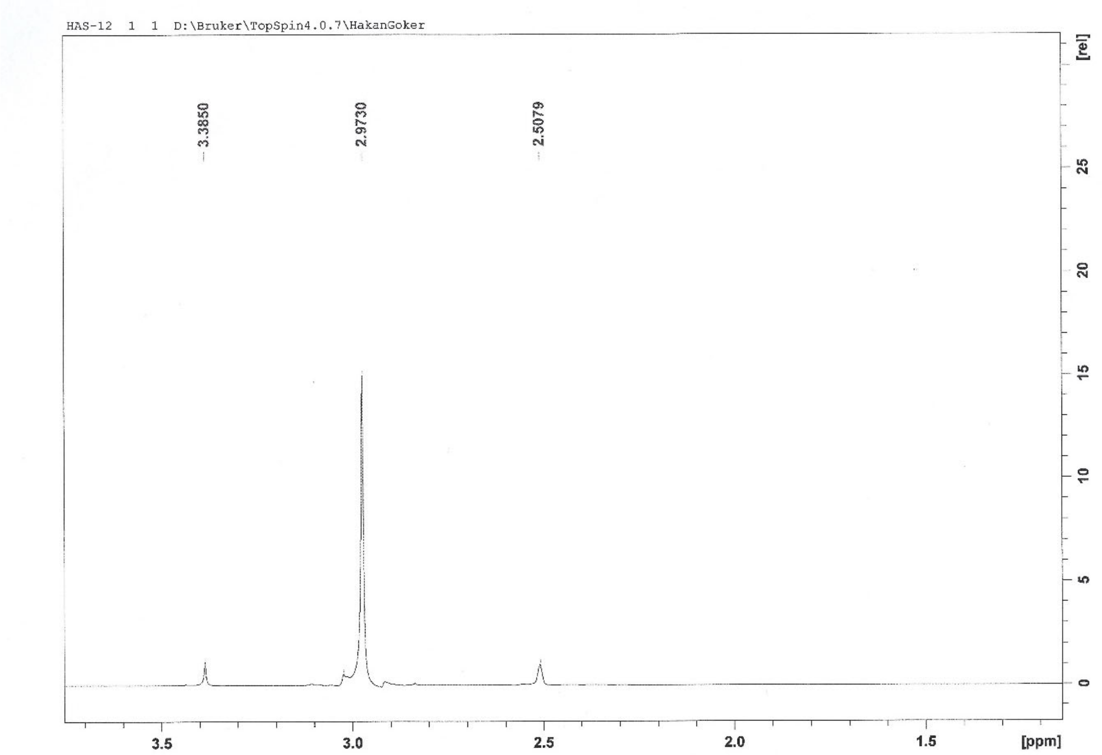

Figure S72: ^{1}H NMR spectrum of compound **2l** (6.5-12 ppm)

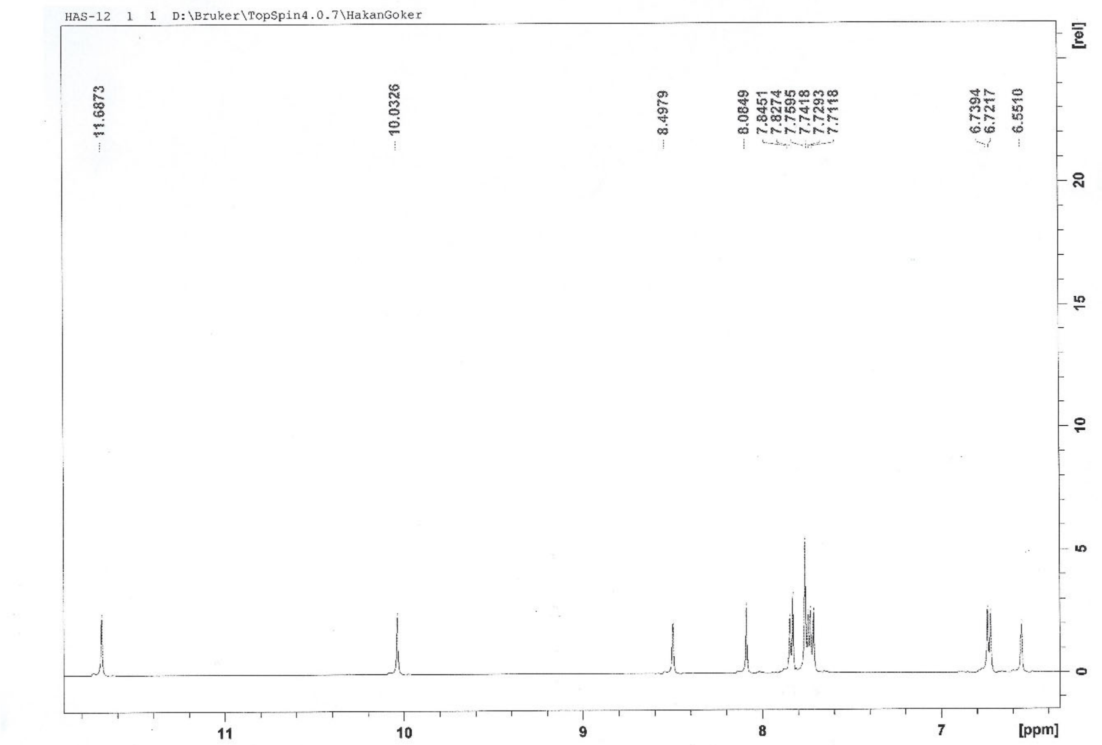

Figure S73: ^{13}C NMR spectrum of compound **2l**

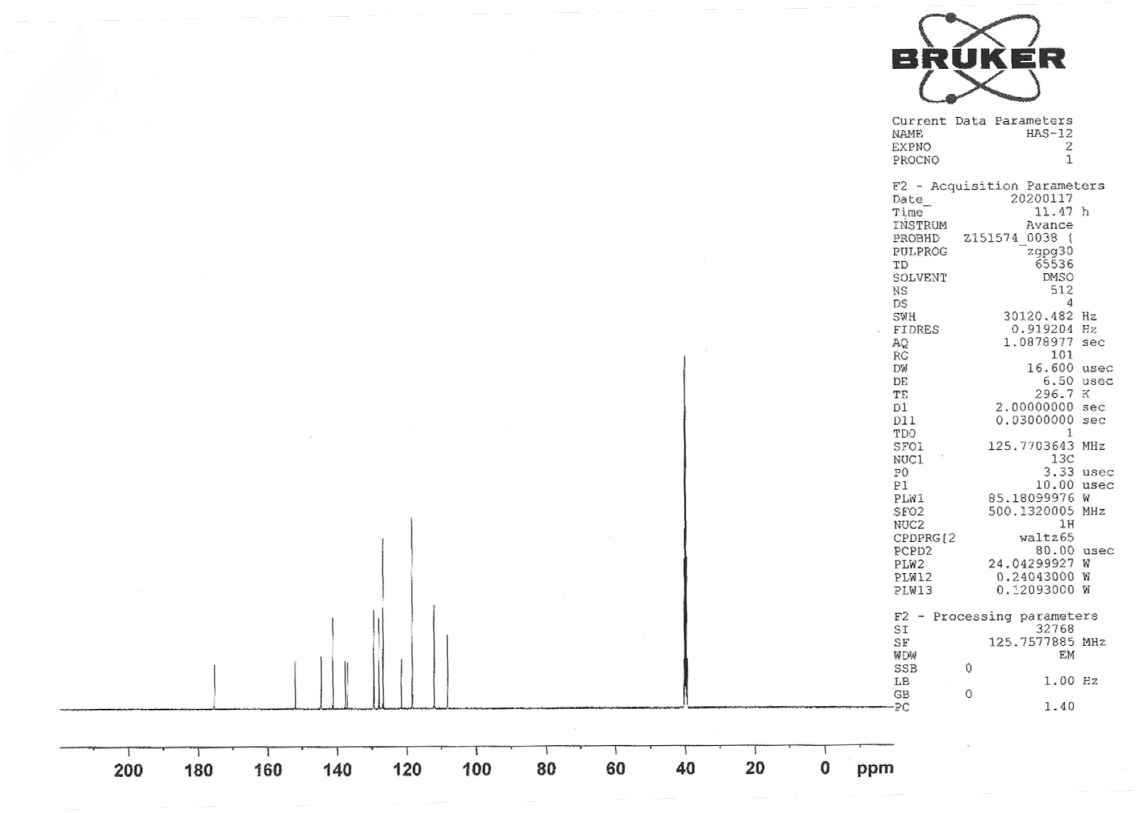

Figure S74: HRMS spectrum of compound **2l**

Formula Predictor Report - has-12_53.lcd

Data File: C:\LabSolutions\Data\Analiz\mdaltintop\has-12_53.lcd

Elmt	Val.	Min	Max	Elmt	Val.	Min	Max	Elmt	Val.	Min	Max	Elmt	Val.	Min	Max	Use Adduct
H	1	6	40	O	2	0	2	S	2	0	2	Ru	2	0	0	H
C	4	7	33	F	1	0	0	Cl	1	0	0	Pd	2	0	0	
N	3	3	7	P	3	0	0	Br	1	0	0	I	3	0	0	

Error Margin (ppm): 5
HC Ratio: unlimited
Max Isotopes: 3
MSn Iso RI (%): 10.00

DBE Range: 5.0 - 20.0
Apply N Rule: yes
Isotope RI (%): 1.00
MSn Logic Mode: AND

Electron Ions: both
Use MSn Info: yes
Isotope Res: 9000
Max Results: 100

Event#: 1 MS(E+) Ret. Time : 4.280 -> 4.587 Scan# : 643 -> 689

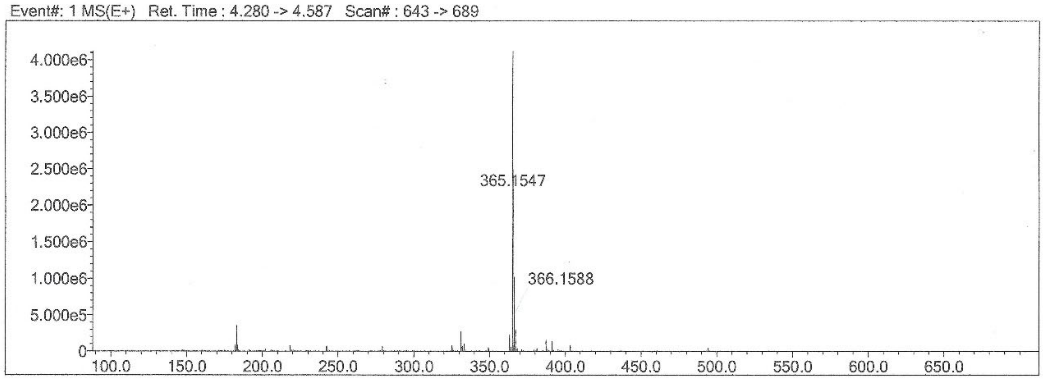

Measured region for 365.1547 m/z

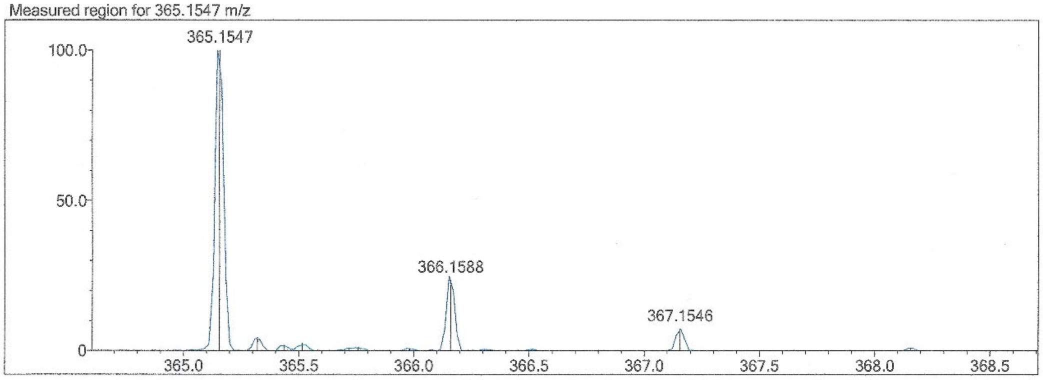

C19 H20 N6 S [M+H]+ : Predicted region for 365.1543 m/z

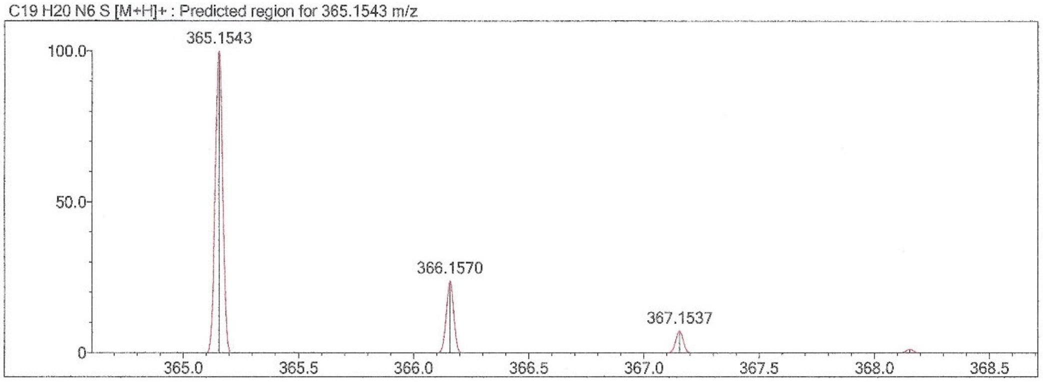

Rank	Score	Formula (M)	Ion	Meas. m/z	Pred. m/z	Df. (mDa)	Df. (ppm)	Iso	DBE
1	94.74	C19 H20 N6 S	[M+H]+	365.1547	365.1543	0.4	1.10	94.98	13.0

Figure S75: IR spectrum of compound **2m**

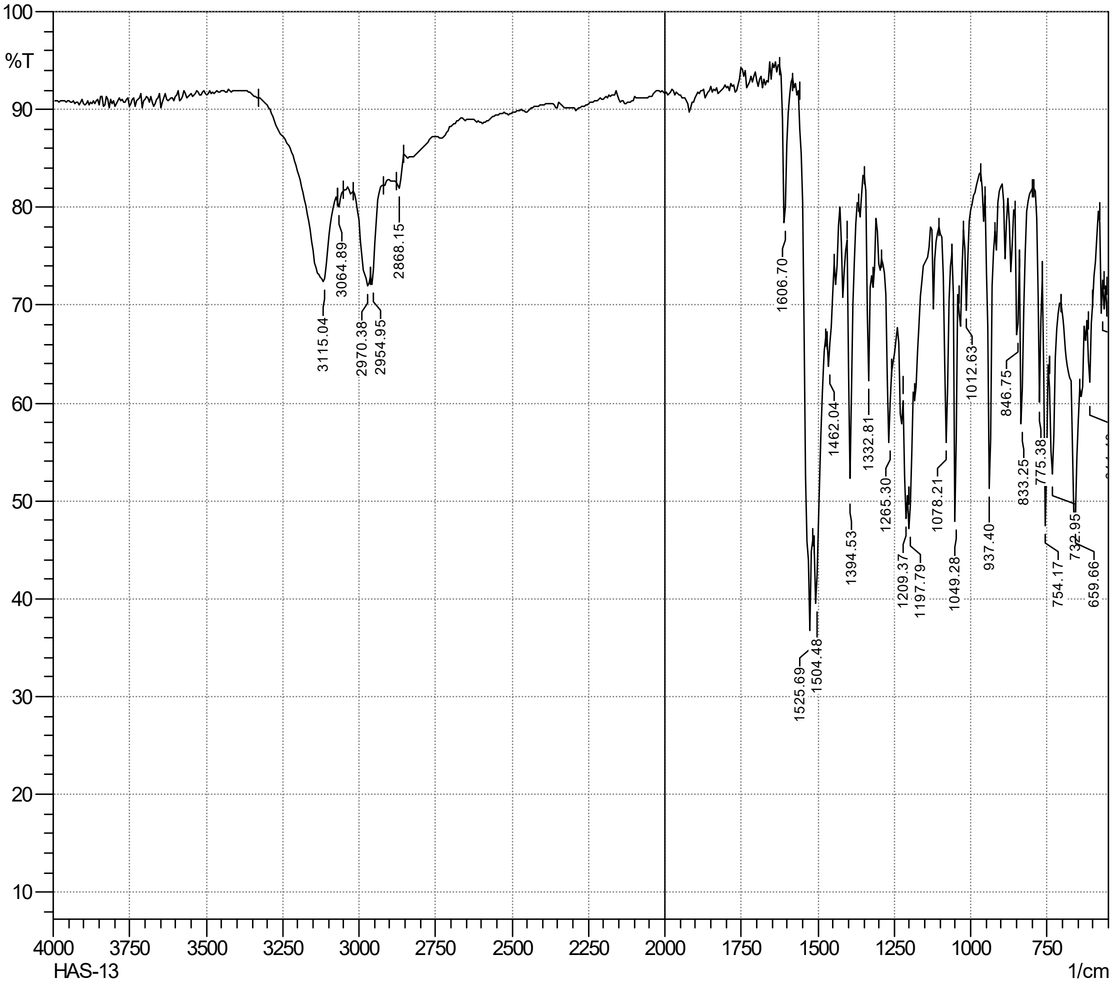

Figure S76: ^{1}H NMR spectrum of compound **2m**

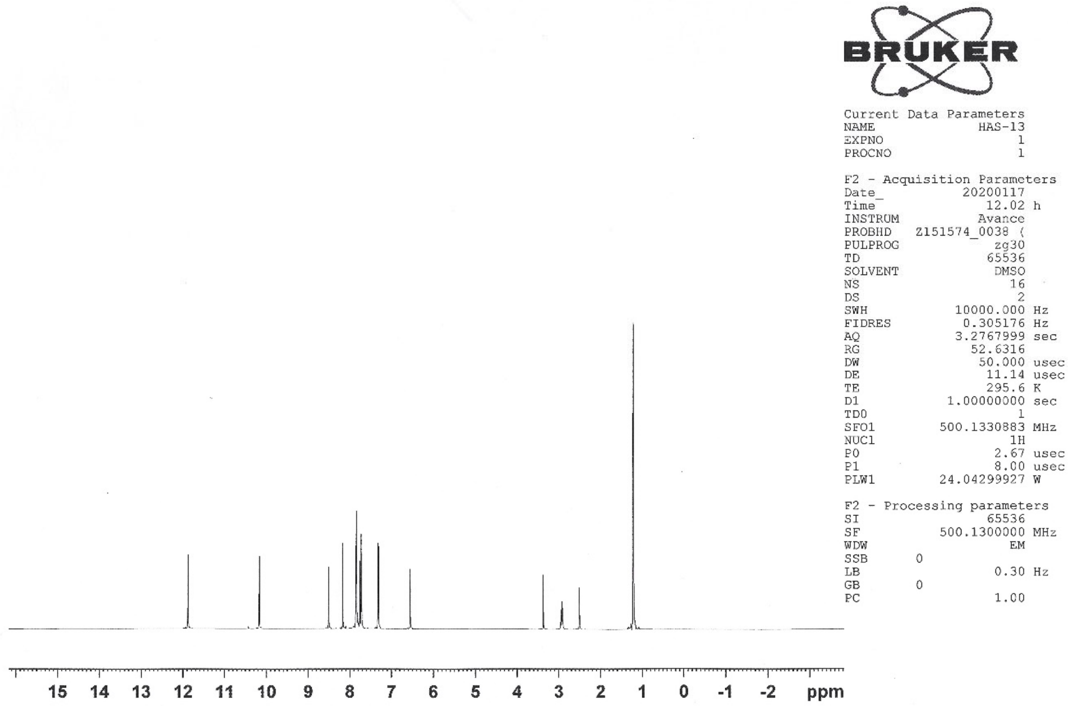

Figure S77: ^{1}H NMR spectrum of compound **2m** with integral values

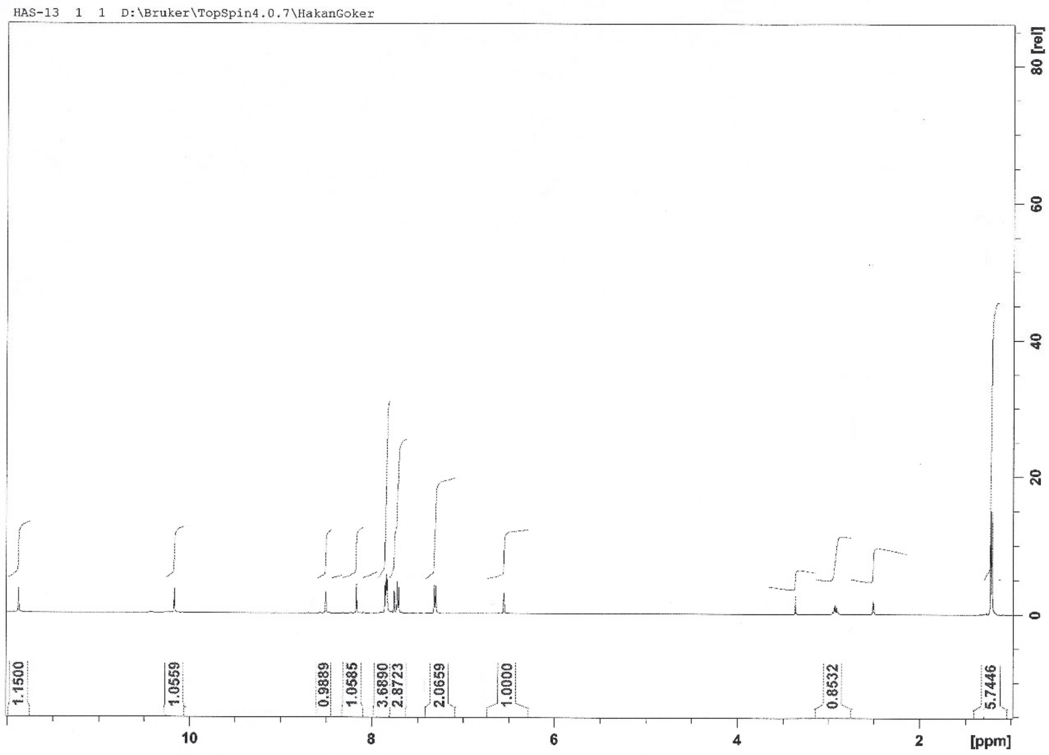

Figure S78: ^{1}H NMR spectrum of compound **2m** (1-3.3 ppm)

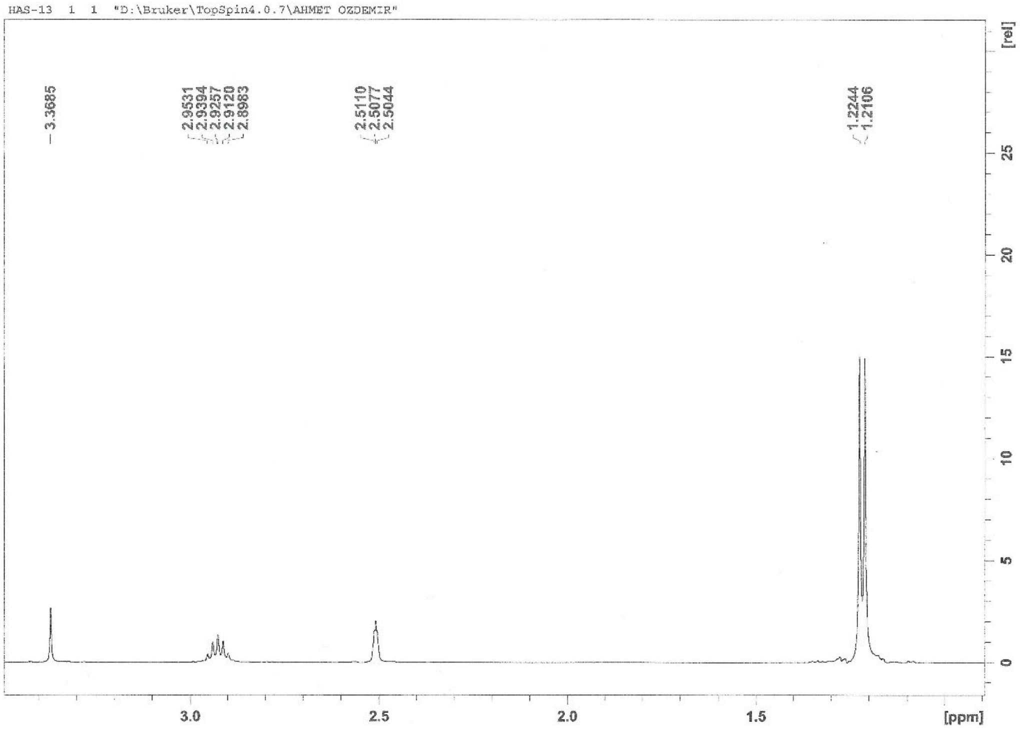

Figure S79: ^{1}H NMR spectrum of compound **2m** (6.5-12 ppm)

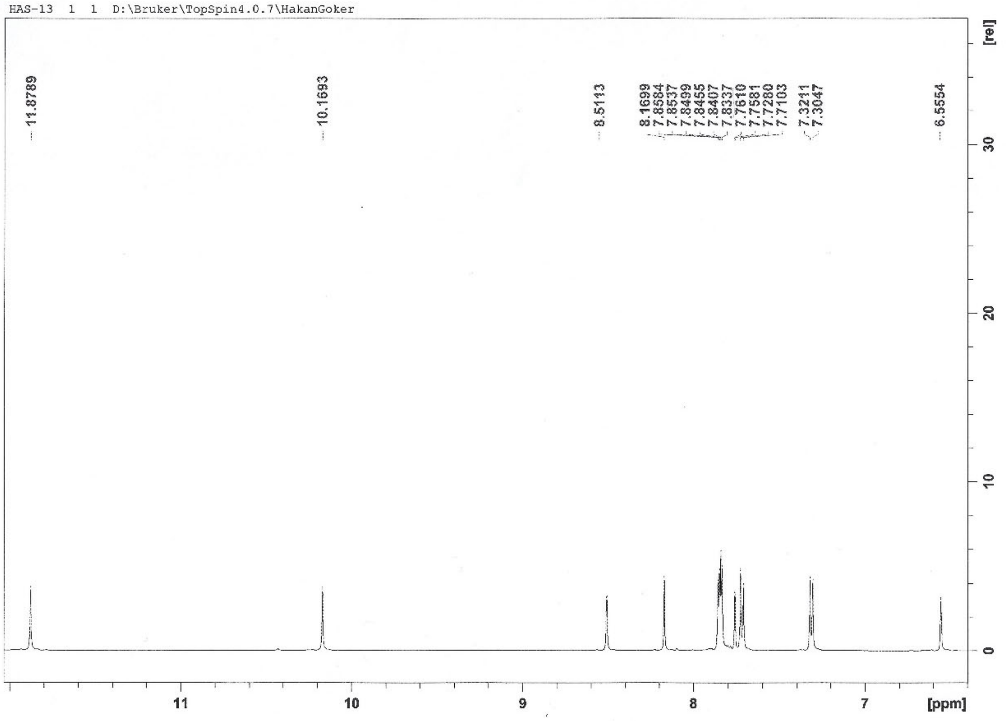

Figure S80: ^{13}C NMR spectrum of compound **2m**

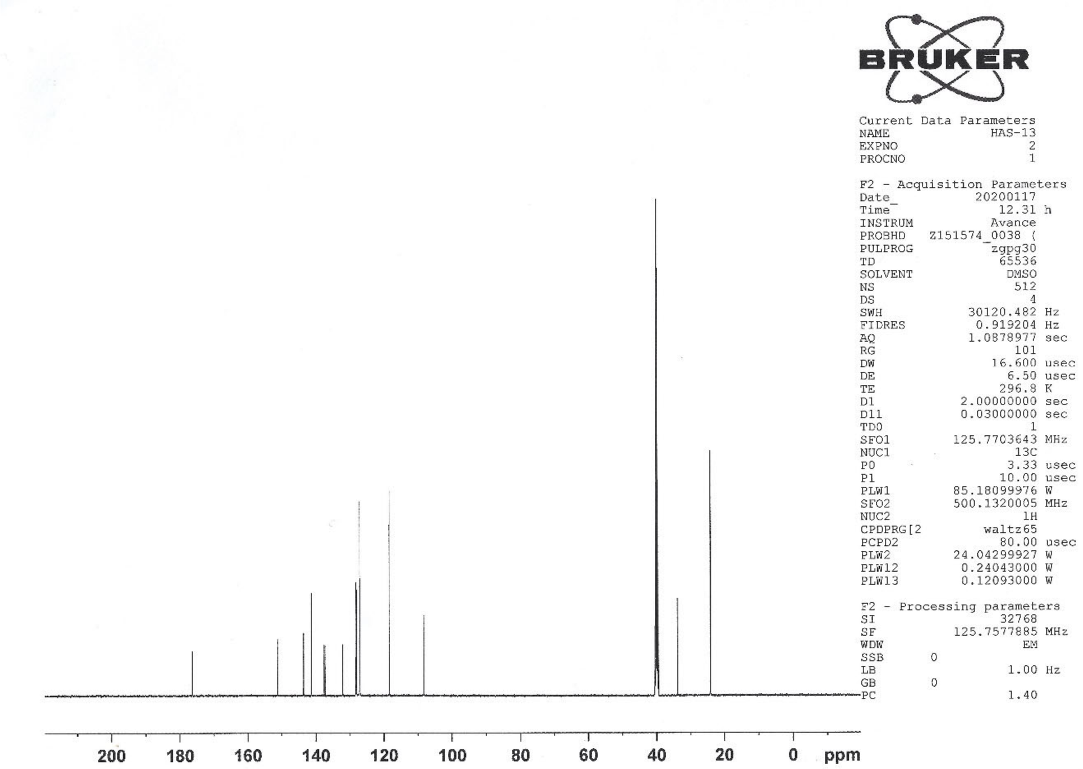

Figure S81: HRMS spectrum of compound **2m**

Data File: C:\LabSolutions\Data\Analiz\mdaltintop\has-13_54.lcd

Elmt	Val.	Min	Max	Elmt	Val.	Min	Max	Elmt	Val.	Min	Max	Elmt	Val.	Min	Max	Use Adduct
H	1	6	40	O	2	0	2	S	2	0	2	Ru	2	0	0	H
C	4	7	33	F	1	0	0	Cl	1	0	0	Pd	2	0	0	
N	3	3	7	P	3	0	0	Br	1	0	0	I	3	0	0	

Error Margin (ppm): 5
HC Ratio: unlimited
Max Isotopes: 3
MSn Iso RI (%): 10.00

DBE Range: 5.0 - 20.0
Apply N Rule: yes
Isotope RI (%): 1.00
MSn Logic Mode: AND

Electron Ions: both
Use MSn Info: yes
Isotope Res: 9000
Max Results: 100

Event#: 1 MS(E+) Ret. Time : 5.267 Scan# : 791

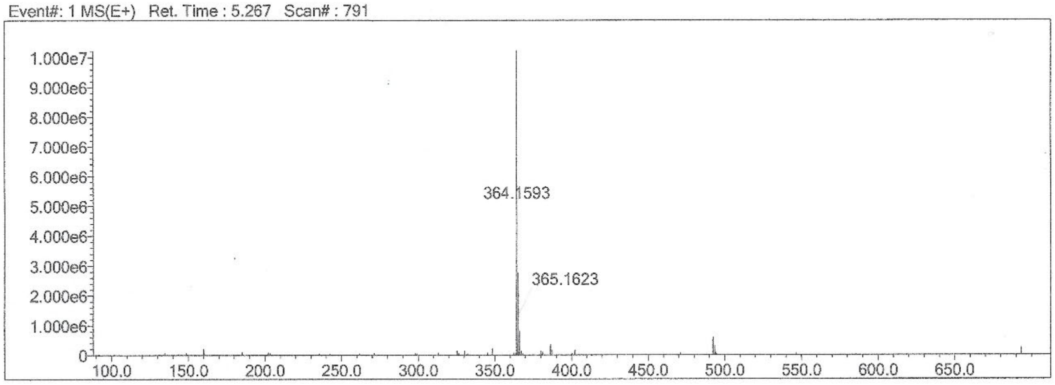

Measured region for 364.1593 m/z

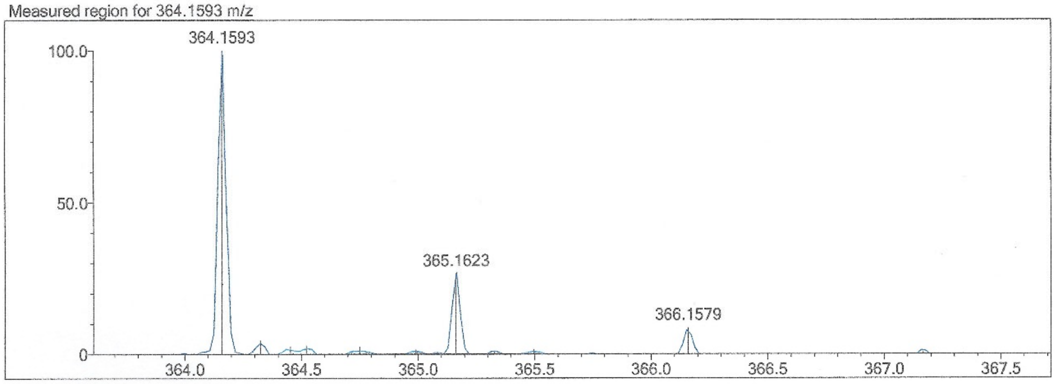

C20 H21 N5 S [M+H]+ : Predicted region for 364.1590 m/z

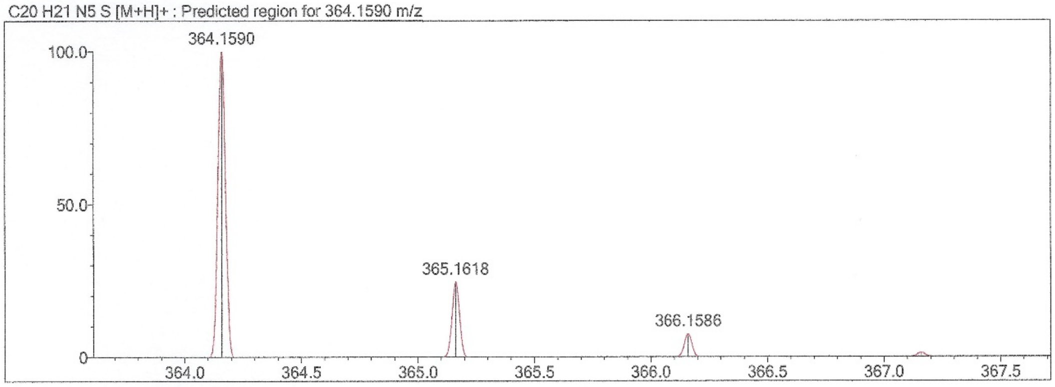

Rank	Score	Formula (M)	Ion	Meas. m/z	Pred. m/z	Df. (mDa)	Df. (ppm)	Iso	DBE
1	88.14	C20 H21 N5 S	[M+H]+	364.1593	364.1590	0.3	0.82	88.14	13.0

Figure S82: IR spectrum of compound **2n**

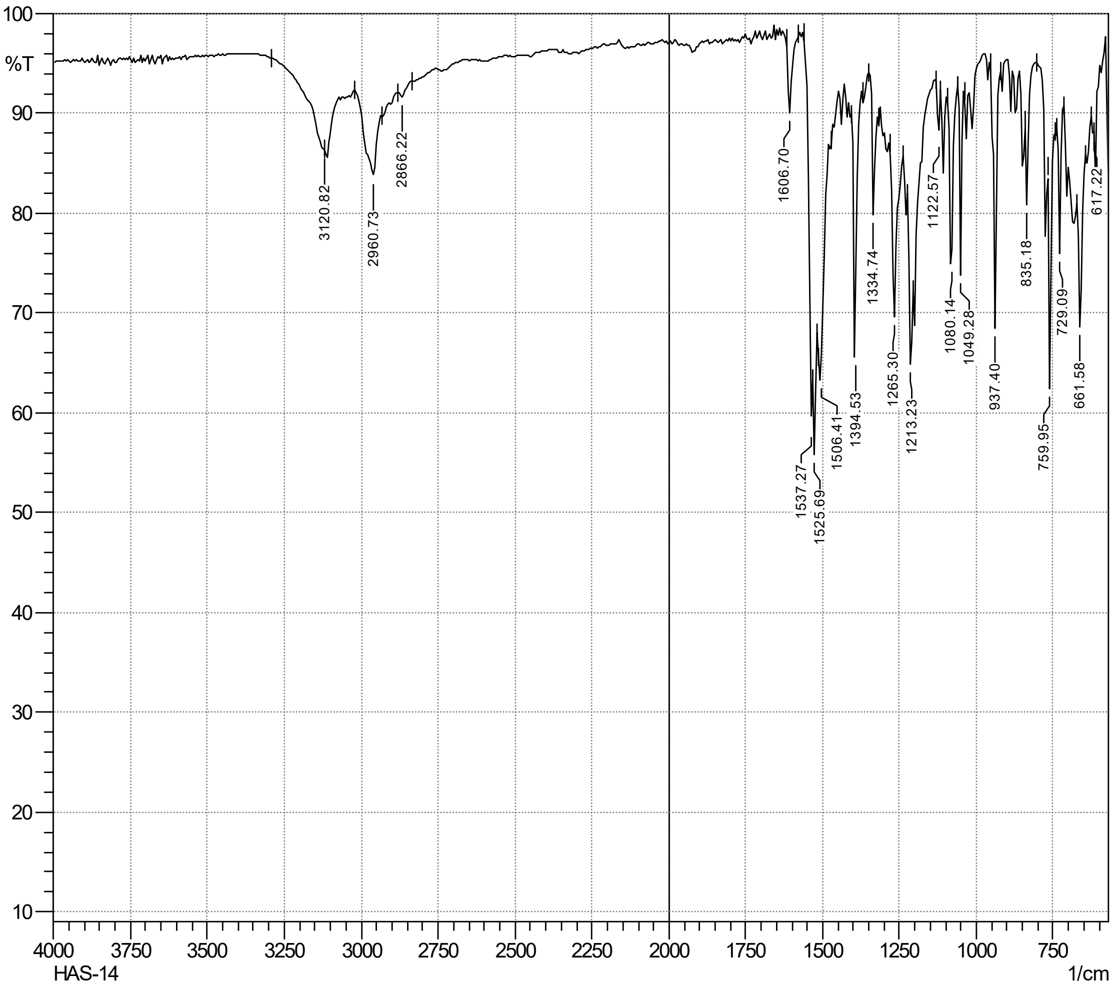

Figure S83: ^{1}H NMR spectrum of compound **2n**

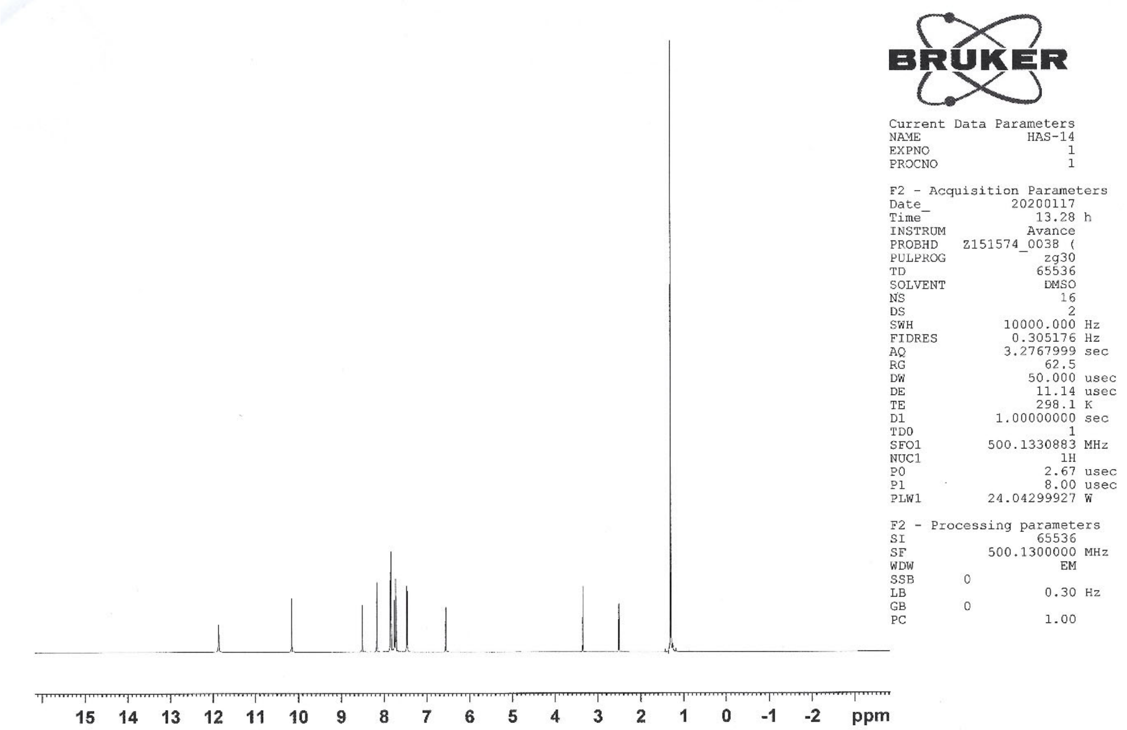

Figure S84: ^{1}H NMR spectrum of compound **2n** with integral values

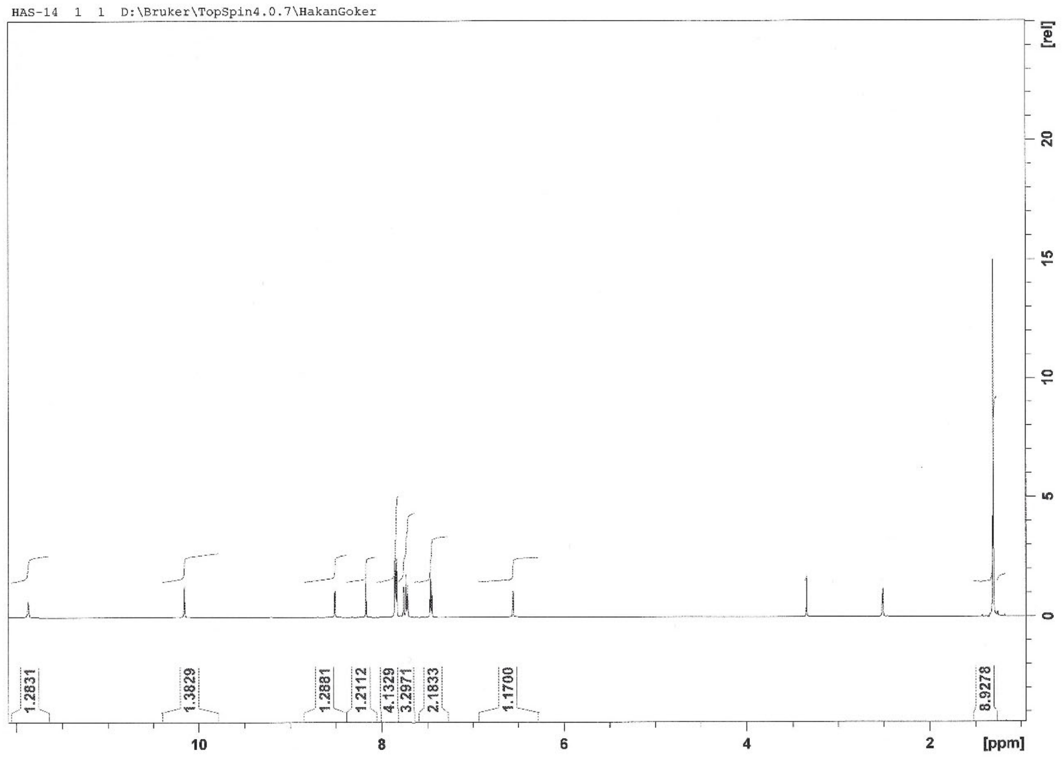

Figure S85: ^{1}H NMR spectrum of compound **2n** (1-3.3 ppm)

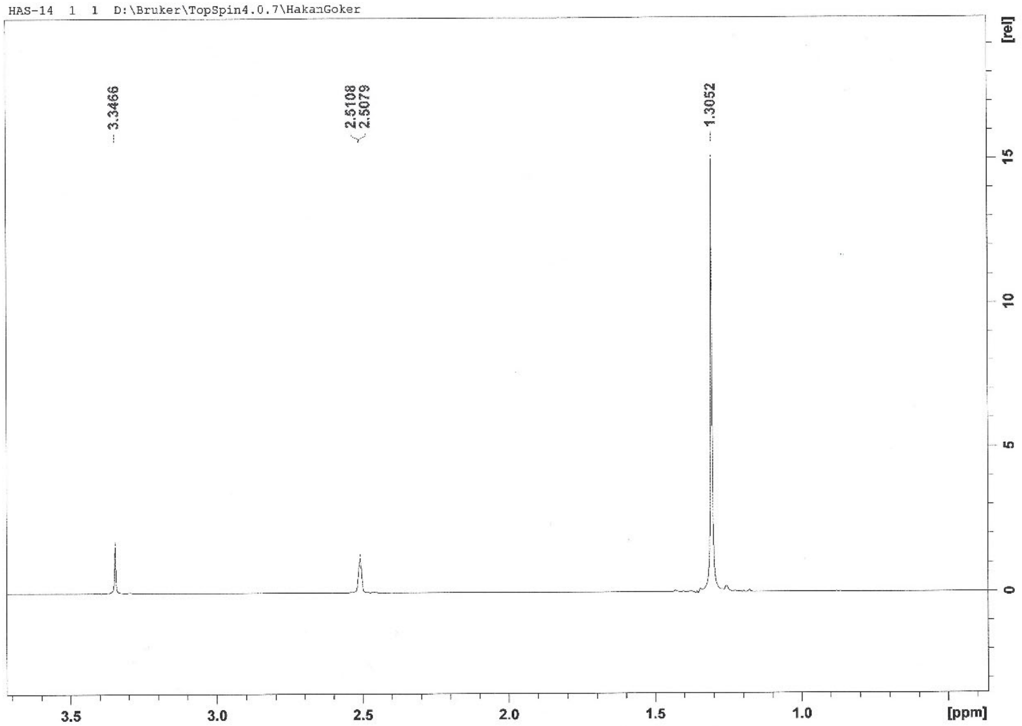

Figure S86: ^{1}H NMR spectrum of compound **2n** (6.5-12 ppm)

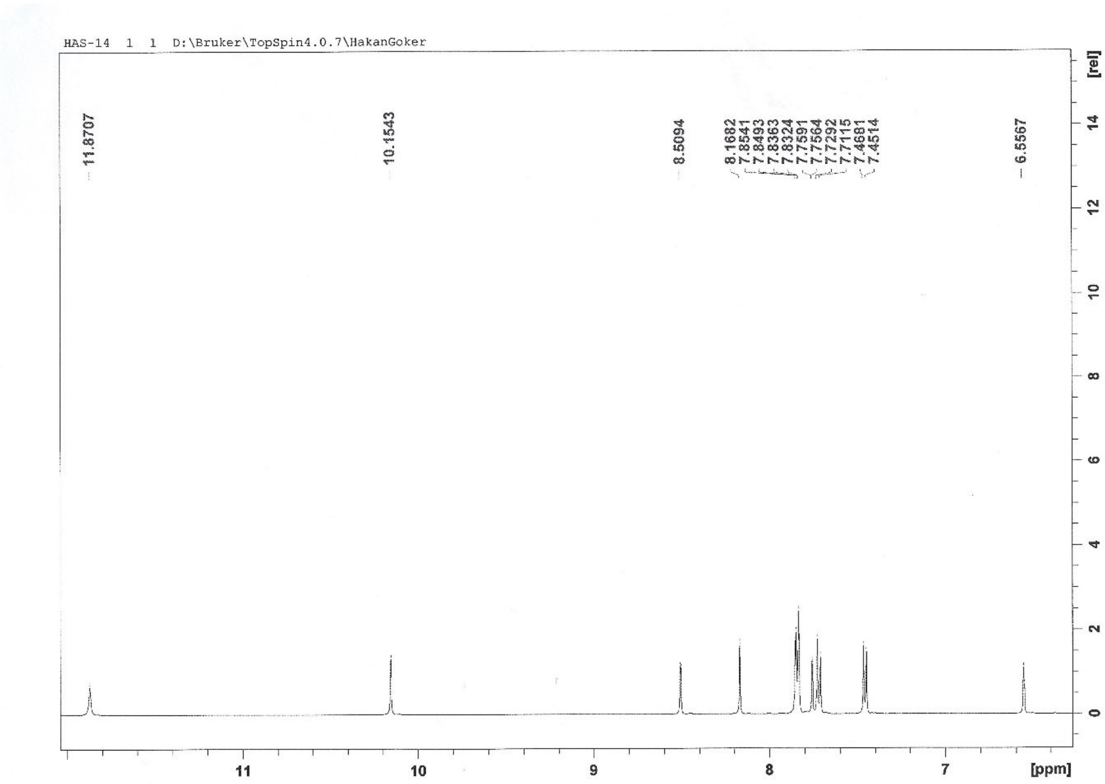

Figure S87: ^{13}C NMR spectrum of compound **2n**

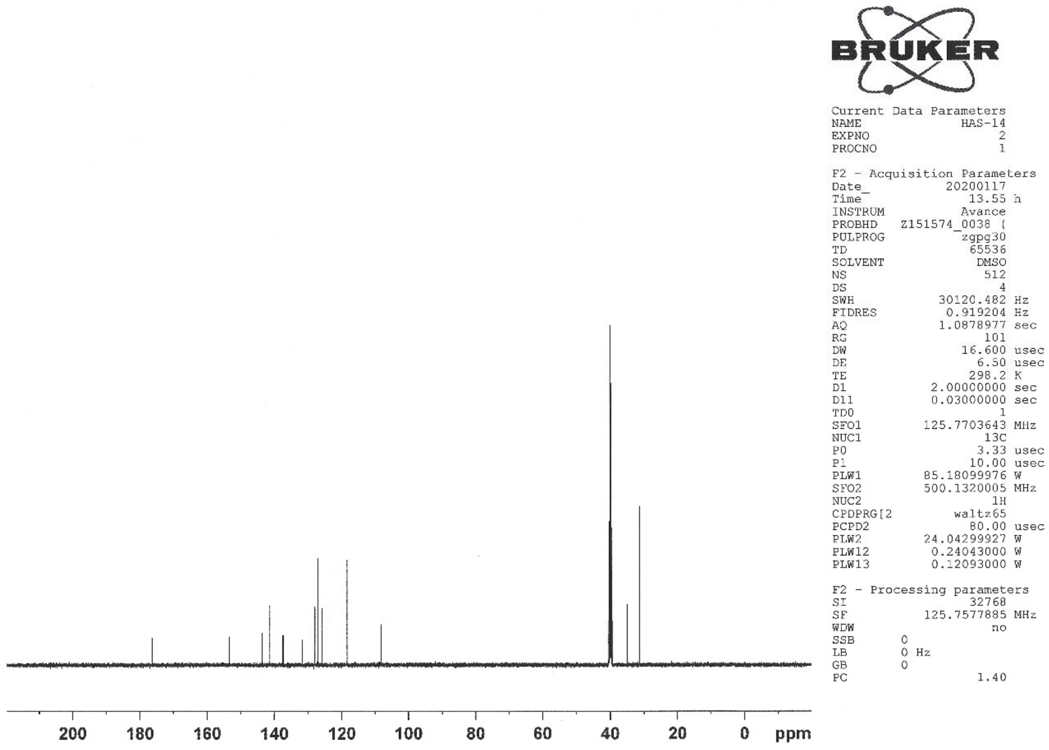

Figure S88: HRMS spectrum of compound **2n**

Formula Predictor Report - has-14_55.lcd

Page 1 of 1

Data File: C:\LabSolutions\Data\Analiz\mdaltintop\has-14_55.lcd

Elmt	Val.	Min	Max	Elmt	Val.	Min	Max	Elmt	Val.	Min	Max	Elmt	Val.	Min	Max	Use Adduct
H	1	6	40	O	2	0	2	S	2	0	2	Ru	2	0	0	H
C	4	7	33	F	1	0	0	Cl	1	0	0	Pd	2	0	0	
N	3	3	7	P	3	0	0	Br	1	0	0	I	3	0	0	

Error Margin (ppm): 5
HC Ratio: unlimited
Max Isotopes: 3
MSn Iso RI (%): 10.00

DBE Range: 5.0 - 20.0
Apply N Rule: yes
Isotope RI (%): 1.00
MSn Logic Mode: AND

Electron Ions: both
Use MSn Info: yes
Isotope Res: 9000
Max Results: 100

Event#: 1 MS(E+) Ret. Time : 5.360 -> 5.680 Scan# : 805 -> 853

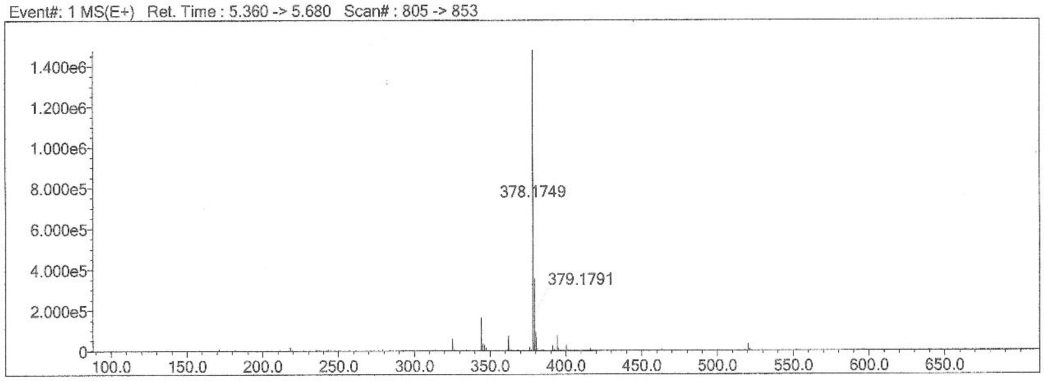

Measured region for 378.1749 m/z

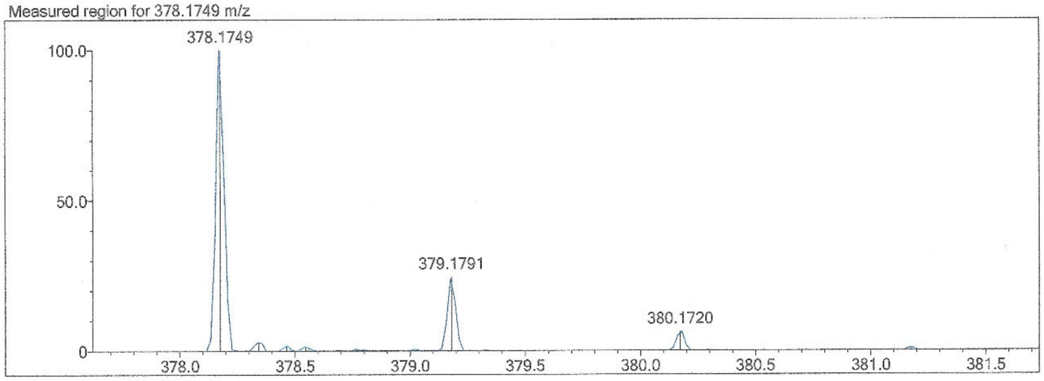

C21 H23 N5 S [M+H]+ : Predicted region for 378.1747 m/z

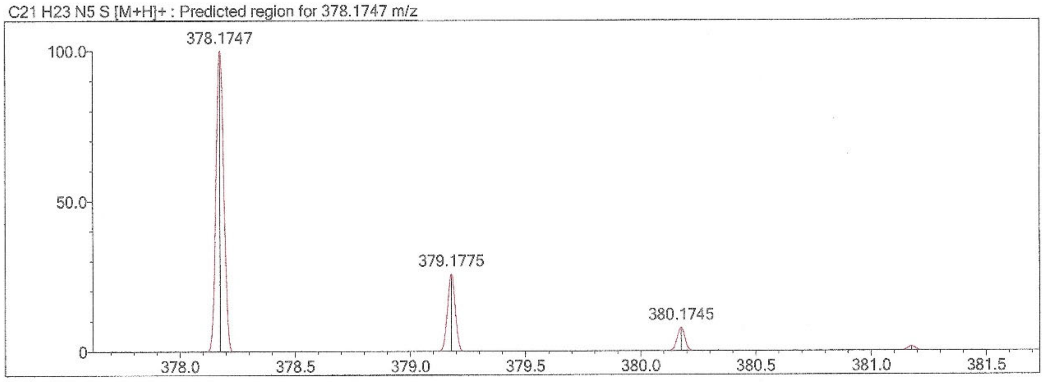

Rank	Score	Formula (M)	Ion	Meas. m/z	Pred. m/z	Df. (mDa)	Df. (ppm)	Iso	DBE
1	77.93	C21 H23 N5 S	[M+H]+	378.1749	378.1747	0.2	0.53	77.93	13.0

Figure S89: IR spectrum of compound **2o**

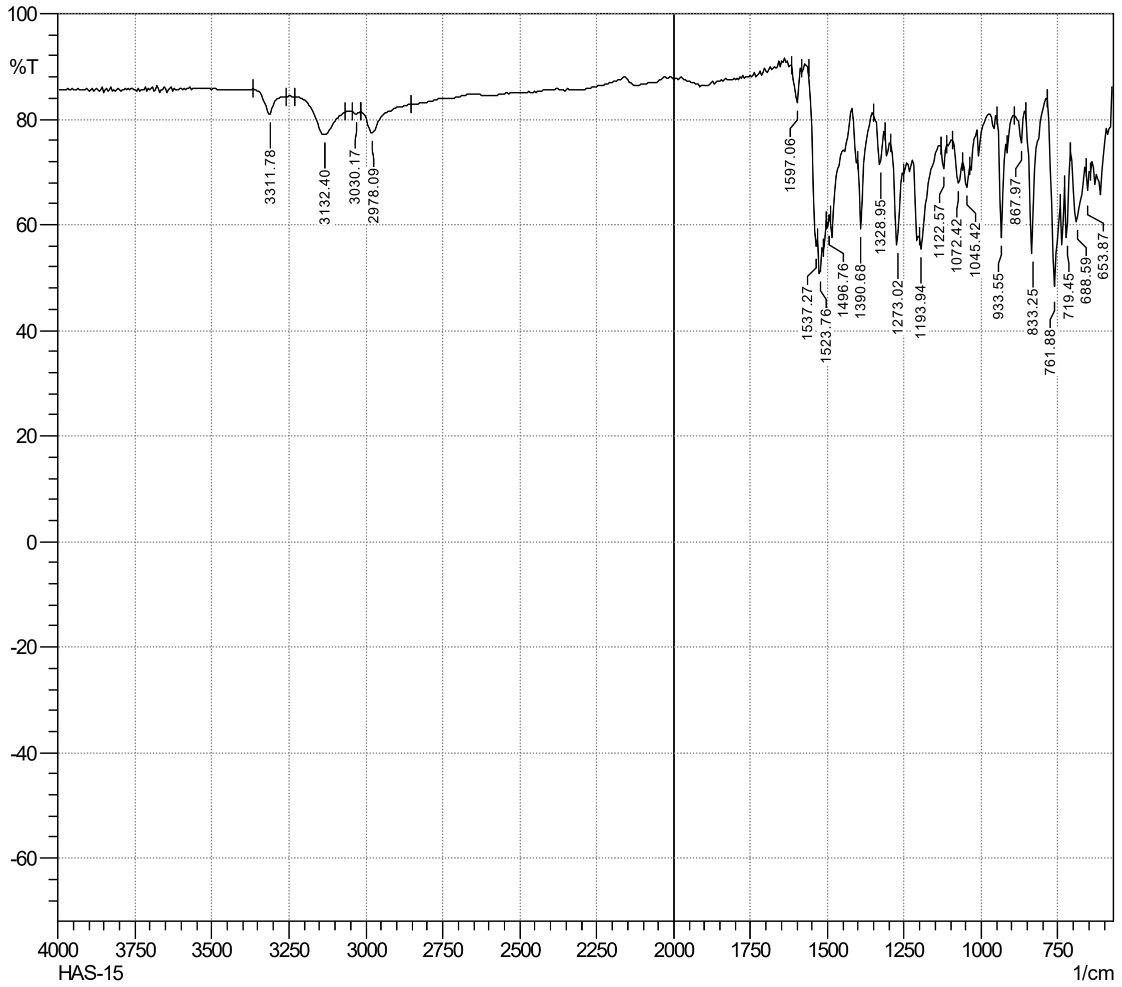

Figure S90: ^{1}H NMR spectrum of compound **2o**

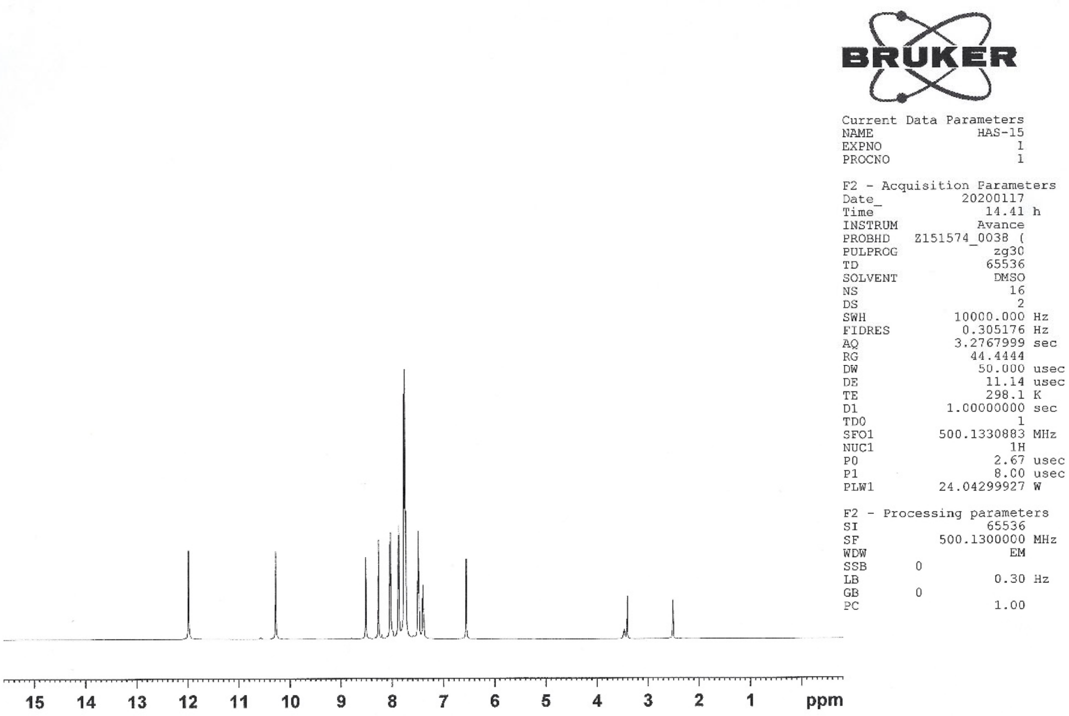

Figure S91: ^{1}H NMR spectrum of compound **2o** with integral values

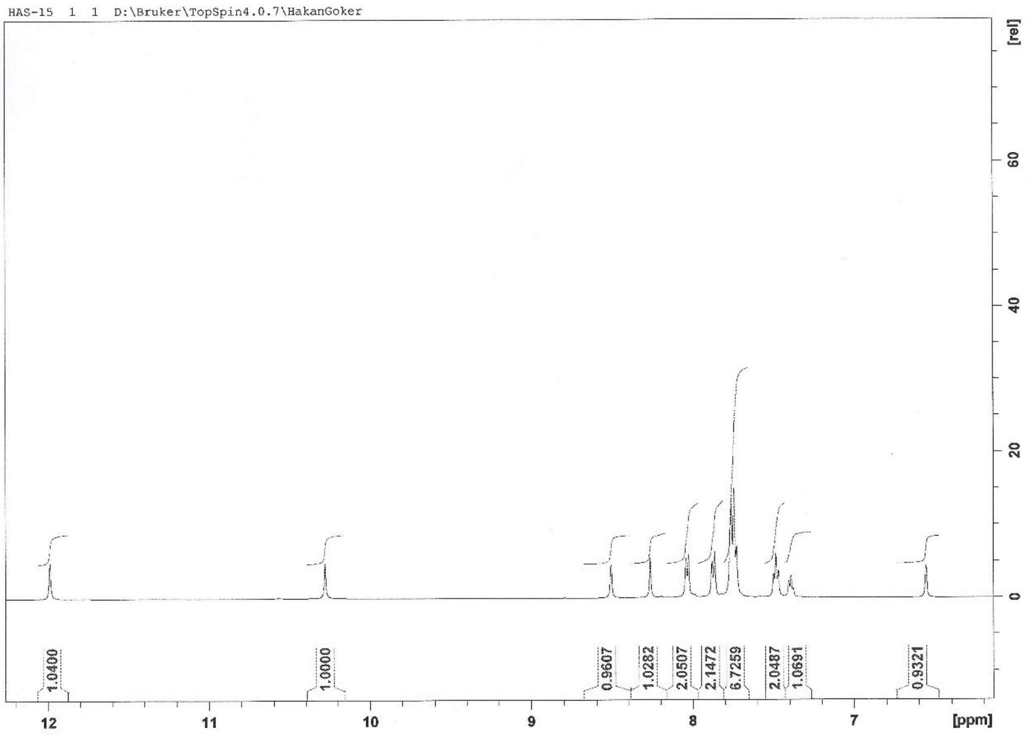

Figure S92: ^{1}H NMR spectrum of compound **2o** (6.5-12 ppm)

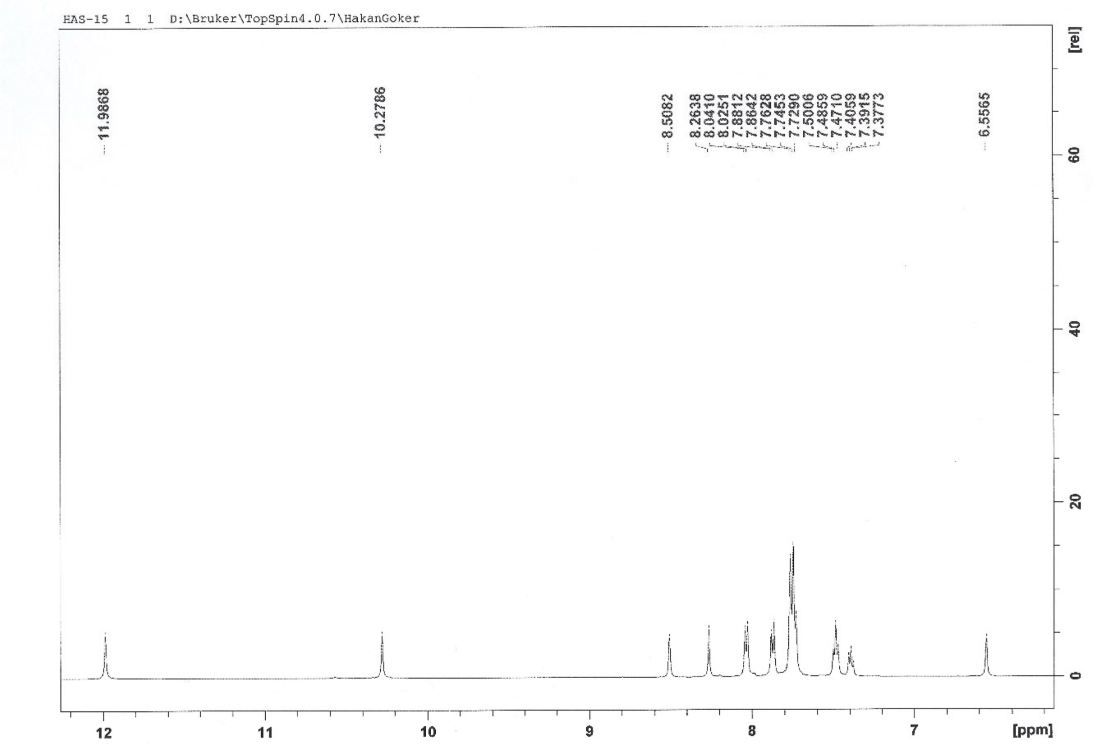

Figure S93: ^{13}C NMR spectrum of compound **2o**

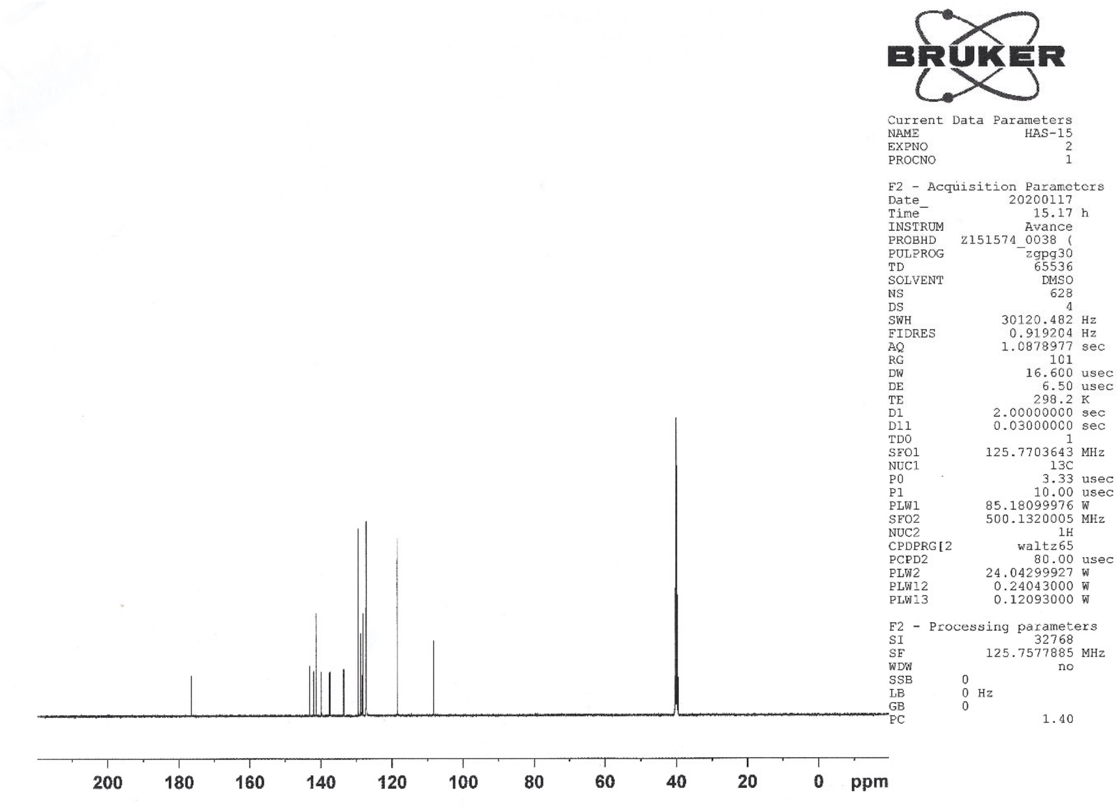

Figure S94: HRMS spectrum of compound **2o**

Formula Predictor Report - has-15_56.lcd

Data File: C:\LabSolutions\Data\Analiz\mdaltintop\has-15_56.lcd

Elmt	Val.	Min	Max	Elmt	Val.	Min	Max	Elmt	Val.	Min	Max	Elmt	Val.	Min	Max	Use Adduct
H	1	6	40	O	2	0	0	S	2	0	2	Ru	2	0	0	H
C	4	7	33	F	1	0	0	Cl	1	0	0	Pd	2	0	0	
N	3	3	7	P	3	0	0	Br	1	0	0	I	3	0	0	

Error Margin (ppm):	5	DBE Range:	5.0 - 20.0	Electron Ions:	both
HC Ratio:	unlimited	Apply N Rule:	yes	Use MSn Info:	yes
Max Isotopes:	3	Isotope RI (%):	1.00	Isotope Res:	9000
MSn Iso RI (%):	10.00	MSn Logic Mode:	AND	Max Results:	100

Event#: 1 MS(E+) Ret. Time : 5.133 -> 5.453 Scan# : 771 -> 819

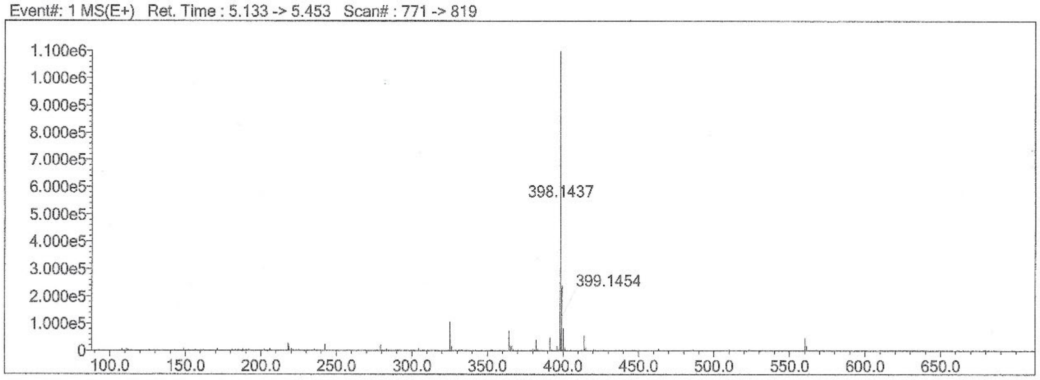

Measured region for 398.1437 m/z

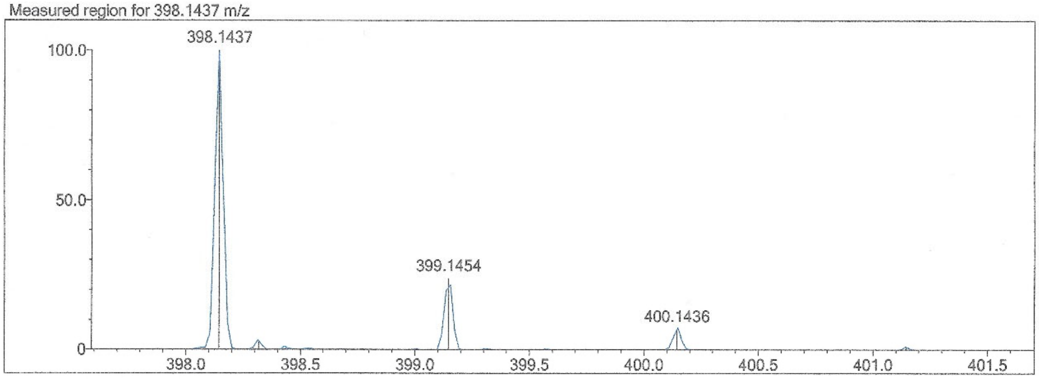

C23 H19 N5 S [M+H]+ : Predicted region for 398.1434 m/z

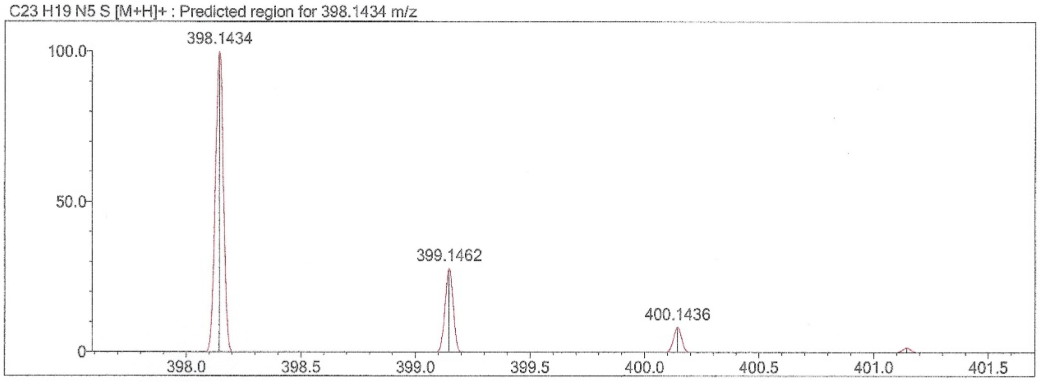

Rank	Score	Formula (M)	Ion	Meas. m/z	Pred. m/z	Df. (mDa)	Df. (ppm)	Iso	DBE
1	88.42	C23 H19 N5 S	[M+H]+	398.1437	398.1434	0.3	0.75	88.42	17.0
